# Of Humans and Gerbils— Independent Diversification of Neuroligin-4 Into X- and Y-Specific Genes in Primates and Rodents

**DOI:** 10.3389/fnmol.2022.838262

**Published:** 2022-03-30

**Authors:** Stephan Maxeiner, Fritz Benseler, Nils Brose, Gabriela Krasteva-Christ

**Affiliations:** ^1^Anatomy and Cell Biology, Saarland University, Homburg, Germany; ^2^Department of Molecular Neurobiology, Max Planck Institute for Multidisciplinary Sciences, Göttingen, Germany

**Keywords:** gametologous genes, pseudoautosomal region (PAR), euarchontoglires, gerbils, neuroligin-4, mammal evolution

## Abstract

The neural cell adhesion protein neuroligin-4 has puzzled neuroscientists and geneticist alike for almost two decades. Its clinical association with autism spectrum disorders (ASD) is well established, however, its diversification into sex chromosome-specific copies, *NLGN4X* and *NLGN4Y*, remains uncharted territory. Just recently, the presence of substantial neuroligin-4 sequence differences between humans and laboratory mice, in which *Nlgn4* is a pseudoautosomal gene, could be explained as a consequence of dramatic changes affecting the pseudoautosomal region on both sex chromosomes in a subset of rodents, the clade eumuroida. In this study, we describe the presence of sex chromosome-specific copies of neuroligin-4 genes in the Mongolian gerbil (*Meriones unguiculatus*) marking the first encounter of its kind in rodents. Gerbils are members of the family Muridae and are closely related to mice and rats. Our results have been incorporated into an extended evolutionary analysis covering primates, rodents, lagomorphs, treeshrews and culogos comprising together the mammalian superorder euarchontoglires. We gathered evidence that substantial changes in neuroligin-4 genes have also occurred outside eumuroida in other rodent species as well as in lagomorphs. These changes feature, e.g., a general reduction of its gene size, an increase in its average GC-content as well as in the third position (GC3) of synonymous codons, and the accumulation of repetitive sequences in line with previous observations. We further show conclusively that the diversification of neuroligin-4 in sex chromosome-specific copies has happened multiple times independently during mammal evolution proving that Y-chromosomal *NLGN4Y* genes do not originate from a single common *NLGN4Y* ancestor.

## Introduction

Neuroligin-4 is a member of the neuroligin family of synaptic cell adhesion proteins that regulate the development, maturation, and specification of synapses (Krueger-Burg et al., 2017; Südhof, 2017). They are localized to the postsynaptic side of nerve cell synapses and interact with presynaptic adhesion proteins, such as neurexins (Craig and Kang, 2007). The neuroligin gene family comprises four members, some of which exhibit synapse-type specificity (Song et al., 1999; Graf et al., 2004; Varoqueaux et al., 2004; Budreck and Scheiffele, 2007; Chubykin et al., 2007; Tabuchi et al., 2007; Poulopoulos et al., 2009). All neuroligins are subject to function-altering alternative splicing (Boucard et al., 2005; Lee et al., 2010; Wahlin et al., 2010; Oku et al., 2020).

The *NLGN4X* and *NLGN4Y* genes, X- and Y-chromosome specific copies of an ancestral pseudoautosomal gene (Raudsepp and Chowdhary, 2015), have so far only been identified in two mammalian suborders, i.e., in primates and even-toed ungulates (e.g., horses) (Raudsepp and Chowdhary, 2015). Sex chromosome-specific diversification of ancestral pseudoautosomal genes can emerge as a positional consequence of sex chromosome evolution in eutheria, i.e., mammals excluding monotremes and marsupials (Raudsepp and Chowdhary, 2015). Evolutionary, both sex chromosomes derived from a pair of autosomes, developed into proto-sex chromosomes, and eventually separated into the tightly regulated, gene dosage dependent X-chromosome and the Y-chromosome, bearing the inductor of maleness, SRY, *sex-determining region on Y* gene (Wilson Sayres and Makova, 2013; Graves, 2016). However, both sex chromosomes retained a small subtelomeric region with an identical gene cluster that enables autosome-like pairing during male meiosis and is referred to as the pseudoautosomal region, PAR (Burgoyne, 1982; Flaquer et al., 2008). During mammal evolution and separation into different orders, the boundary between the PARs (PAB) and the specific X-and Y-chromosome regions progressively shifted toward the telomeres. In this process, former PAR genes remained on the X-chromosome, whereas only few homologs survived as “gametologs,” trapped on the male-specific part of the Y-chromosome due to a lack of recombination during male meiosis. *AMELX/Y* and *NLGN4X/Y* are examples of such gametologs (Maxeiner et al., 2021). They retain sequence similarity based on which nucleotide or length polymorphisms can be identified by PCR (Mannucci et al., 1994; Maxeiner et al., 2019; Zaffalon et al., 2019).

Beyond the peculiarities of neuroligin-4 gene evolution (Maxeiner et al., 2020), mutations in human neuroligin genes, particularly in *NLGN3* and *NLGN4*, have been linked to monogenic autism spectrum disorders (ASD) and other neurodevelopmental conditions (Jamain et al., 2003; Avdjieva-Tzavella et al., 2012; Steinberg et al., 2012; Xu et al., 2014; Bourgeron, 2015; Kilaru et al., 2016; Nakanishi et al., 2017; Parente et al., 2017). Whether the NLGN4Y and NLGN4X proteins differ functionally is currently under investigation (Nguyen et al., 2020). This is partly due to the fact that the sequence of Nlgn4 protein in the most widely used genetic mammalian animal model, the mouse, differs considerably from its closest human homolog, NLGN4X (Bolliger et al., 2008; Jamain et al., 2008), and that it is pseudoautosomal (Maxeiner et al., 2020). We demonstrated recently that this sequence deviation of the mouse *Nlgn4* gene, which includes an accumulation of GC bases in the third codon position and repetitive elements are the result of general changes in the PAR. Indeed, many PAR genes in mice were either lost or translocated to X-specific regions on the X-chromosomes or to autosomes (Maxeiner et al., 2020). Strikingly, though, these changes have emerged in a very specific subset of rodents, the clade eumuroida (Maxeiner et al., 2020), which is a part of the suborder supermyomorpha (D’Elía et al., 2019). Eumuroida species comprise two thirds of all rodent species (Burgin et al., 2018), resembling about a quarter of all mammals that are affected by substantial PAR erosion (Maxeiner et al., 2020, 2021). However, curated sequence information of rodent genomes has mostly derived from female specimens, making it impossible to judge whether gametolog diversification of neuroligin-4, as observed in humans and horses, has also developed independently in rodents.

Building on available but incomplete sequence information on the male gerbil genome (GenBank, NCBI), we determined the complete sequence of the neuroligin-4 genes of a female and a male gerbil of the species *Meriones unguiculatus* (Mongolian gerbil), identified two hitherto uncharted exons upstream of the exon bearing the designated start codon, and fully annotated the respective *NLGN4X/Y* genes. Our data identify the gametolog neuroligin-4 gene pair in gerbils as the first example of an X- and Y-chromosomal diversification of *NLGN4* in rodents. In the context of a comprehensive evolutionary tree incorporating many more euarchontoglires species, our data document multiple independent diversification events of neuroligin-4 into gametologs as well as an accelerated evolution of its coding region.

## Materials and Methods

### Nucleic Acid Extraction

A pair of 4 week old male and female gerbils (*Meriones unguiculatus*; RjTub:MON) was purchased from Janvier Labs, France. Upon arrival animals were euthanized and brains were dissected and collected in TRIzol^®^ reagent (Invitrogen, Carlsbad, CA, United States) for RNA extraction. Liver biopsies were collected in tissue lysis buffer for DNA extraction, and subsequent DNA isolation was performed using the phenol-chloroform extraction method (Maxeiner et al., 2019). All animal handling followed standards set by and protocols approved by local authorities as well as Saarland University, Homburg, Germany.

### 5′ RACE-Experiment and PCR Cloning

To determine the transcriptional start site of neuroligin-4 in gerbils, RNA from the female gerbil was analyzed by 5′ RACE (5′ rapid amplification of cDNA ends; SMARTer^®^ RACE 5′/3′ Kit, Takara Bio Europe SAS, Saint-Germain-en-Laye, France) following the supplier’s instructions for the amplification of 5′ cDNA ends. RNA was transcribed into cDNA and an artificial DNA sequence was added. The cDNA then served as template for gene specific primers and a universal primer provided by the kit. Amplification of gene products was performed using SeqAmp DNA Polymerase from the kit or Q5 High-Fidelity 2x Master Mix (New England Biolabs GmbH, Frankfurt, Germany). Amplification products of the 5′ RACE experiment and other PCR amplicons used for genomic sequencing were separated on agarose gels and relevant bands were extracted using the Zymo Gel extraction kit (Zymo Research Europe GmbH, Freiburg, Germany). Amplicons were cloned using the In-Fusion cloning kit (Takara Bio Europe SAS). Amplification of plasmid DNA and bacterial culture was performed according to standard procedures, and plasmid DNA for subsequent applications was prepared using resin-based DNA purification systems (Zymo Research). Nucleic acid quantification was performed using the NanoDrop One spectrophotometer (VWR International). Gene specific oligonucleotides were purchased from IDT and are listed in Supplementary Data Sheet 1. Sequencing reactions were performed at the AGCT-Lab sequencing core facility of the Max Planck Institute for Multidisciplinary Sciences, Göttingen, Germany.

### Sex-Typing and PCR Analysis

Oligonucleotides were designed based on sequence homology between gerbil *NLGN4X* and *NLGN4Y* (MX18723/MX18724; cf. Supplementary Data Sheet 1) to amplify DNA in PCR reactions from the gene pair in a single run. PCR reactions were set up using 5 ng of genomic template DNA and Q5 High-Fidelity 2x Master Mix (New England Biolabs) with the following cycle conditions: 98°C for 30 s, 35x cycle (98°C for 10 s, 65°C for 20 s, 72°C for 30 s), and final extension at 72°C for 1 min. All PCR reactions were performed on a T100 Thermal Cycler (Bio-Rad Laboratories, Feldkirchen, Germany). Amplification products were separated on an agarose gel as published previously (Maxeiner et al., 2019).

### Bioinformatic Analysis

All bioinformatic analyses were performed essentially as described previously (Maxeiner et al., 2020). Searches for sequence similarities were done using the BlastN suite (NCBI),^1^ with default settings for “blast” and “megablast” within specified nucleotide collections and/or WGS for certain species or families. Sequences were aligned using Multalin (Corpet, 1988).^2^ Alternatively, Clustal Omega^3^ was used in conjunction with the use of MEGA X to infer phylogenetic relationships (Kumar et al., 2018). The following web-based interfaces were used to assess additional features of proteins or nucleic acid sequences: determining the cleavage site of signal peptides (Almagro Armenteros et al., 2019),^4^ the GC-content of any given DNA sequence,^5^ the GC3-content and relative codon usage,^6^ a visual representation of the GC-profile,^7^ and the translation of a DNA sequence into a protein sequence.^8^

### Evolutionary Classification

English and Latin names of different species were taken from the classification system used along the deposited sequences in GenBank (NCBI), which follows the systematics and classification of Mammal Species of the World (Wilson and Reeder, 2005). Statements on the relative abundance of mammal species derive from the list of different species presented by Burgin et al. (2018). The number of rodent families and their evolutionary relationship was taken from D’Elía et al. (2019), who combined the formerly separate suborders myomorpha, castorimorpha, and anomaluromorpha under the umbrella term supermyomorpha.

### Data Deposition

All sequences were deposited with GenBank (NCBI) under the following accession numbers: MN045396 (NLGN4Y, closes gap in NHTI01010755.1), MN045397 (NLGN4X, connects NHTI0102956.1, and NHTI01041465.1), MN045398 (NLGN4X, connects NHTI01041465.1 with NHTI01023488.1), and MN045399 (NLGN4X, 5′RACE amplicon covering exon1a, 1b and most of exon 1c).

## Results

### Neuroligin-4 Gene Gametologs in the Gerbil

We previously reported changes in neuroligin-4 genes in a subset of rodents, the clade eumuroida, that were caused by an erosion of the pseudoautosomal region (Maxeiner et al., 2020). In parallel to this work, a draft of the genome of the Mongolian gerbil (*Meriones unguiculatus*) was released, which belongs with rats and mice to the Muridae family (Wilson and Reeder, 2005). This genome release contained several fragments of incompletely annotated “neuroligin-4-like” genes (Supplementary Data Sheet 2), reminiscent of the diversification of neuroligin-4 into X- and Y-chromosome-specific genes seen in humans and horses (Raudsepp and Chowdhary, 2015). The number of sequences and the fact that the genome of a male gerbil had served as the source of genomic DNA led to the hypothesis that both genes might reflect a yet unprecedented case of neuroligin-4 diversification into *NLGN4X* and *NLGN4Y* in a rodent species.

**FIGURE 1:**
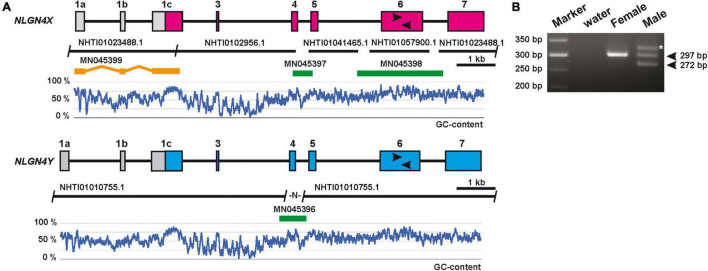
Schematic of the genomic organization of the gerbil *NLGN4X/Y* genes. **(A)** The information of five nucleotide sequences (“NHTI” accession numbers on black bars) were used to assemble a draft of two independent *NLGN4*-like genes in gerbils. Three sequence gaps in *NLGN4X* and one gap in *NLGN4Y* were closed by PCR amplification using gerbil genomic DNA (PCR fragments are depicted in green and labeled with respective accession numbers). A reverse oligo was placed at the 3′ prime end of exon 1c to allow for the amplification of a 5′RACE fragment from brain cDNA of a female gerbil (MN045399). The 5′RACE fragment is displayed as three separate orange bars to appreciate the exon/exon junctions framing two additional upstream exons, 1a and 1b, from the genomic sequence information. A set of black arrows represent the location of oligonucleotides that allow the amplification of *NLGN4X*- and *NLGN4Y*-specific PCR amplicons. Below each gene the relative GC-content is depicted as a moving average of a 30-base frame. **(B)** Representative PCR result of amplicons based on a length polymorphism within exon 6 in the respective *NLGN4X* and *NLGN4Y* genes. The lower molecular weight band reflects the presence of *NLGN4Y* (272 bp), the higher one in both samples *NLGN4X* (297 bp). An asterisk marks an additional band present in the sample of a male gerbil.

To assign potential X- and Y-chromosome-specific copies of neuroligin-4 genes, we isolated genomic DNA from a male and female Mongolian gerbil. We identified a length polymorphism on “exon 6” (second to last coding exon in all neuroligin genes, Bolliger et al., 2008) that was identified applying a comparable PCR approach adopted from previous sex-typing strategies employing neuroligin-4 genes in horses and humans (Supplementary Data Sheet 3; Maxeiner et al., 2019; Zaffalon et al., 2019; Figure 1B). This attempt resulted in a single band for the neuroligin-4 gene information covered by multiple sequence fragments, thus suggesting that this must represent the gerbil *NLGN4X* gene. Consequently, we used genomic DNA from the female gerbil to fill in the *NLGN4X* sequence gaps, and the genomic DNA from the male gerbil to complete the *NLGN4Y* sequence. Sequencing gaps were closed by PCR cloning and subsequent sequencing of the PCR fragments (Figure 1A).

To determine the transcriptional start site, we performed a 5′RACE experiment (5′ rapid amplification of cDNA ends) with RNA from the brain of the female gerbil, resulting in the identification of two additional exons upstream of the exon with the start codon (Figure 1A). Based on this sequence information, we annotated the corresponding *NLGN4Y* gene by sequence comparison. Both genes, *NLGN4X* and *NLGN4Y*, consist of eight exons, respectively (two exons coding for 5′UTR region, six exons coding for the neuroligin-4 ORF). In line with the previously applied nomenclature for neuroligin genes (Bolliger et al., 2008; Maxeiner et al., 2020), we define the ATG-bearing (start codon-bearing) exon as exon 1 and the final exon encoding the transmembrane and C-terminal region as exon 7. Compared to other neuroligin isoforms, a homolog of exon 2 is absent from neuroligin-2 and -4 genes (Bolliger et al., 2008), which needs to be distinguished from a secondary loss of exon 3 absent in neuroligin-4 genes in certain hamster species (Maxeiner et al., 2020). To acknowledge the presence of any non-coding exons upstream of the ATG-carrying exon 1, we propose to further add alphabetical lettering (i.e., exon 1a, 1b, 1c). The exon base counts for gerbil *NLGN4X/Y* are as follows: exon 1a (5′UTR coding), 219/227; exon 1b (5′UTR coding), 114; exon 1c (5′UTR/protein coding), 799/792; exon 3 (protein coding), 60; exon 4 (protein coding), 153; exon 5 (protein coding), 186; exon 6 (protein coding), 1,063/1,030; exon 7 (protein coding/3′UTR), 925/937. Whereas the overall GC-content of both neuroligin-4 genes is slightly above 50% (*NLGN4X*, 56.12%; *NLGN4Y*, 54.11%), the GC-content is substantially increased in all protein-coding exons judged by the GC-profile along all exons and introns (Figure 1A).

### NLGN4X and NLGN4Y Protein Expression in the Gerbil

**FIGURE 2:**
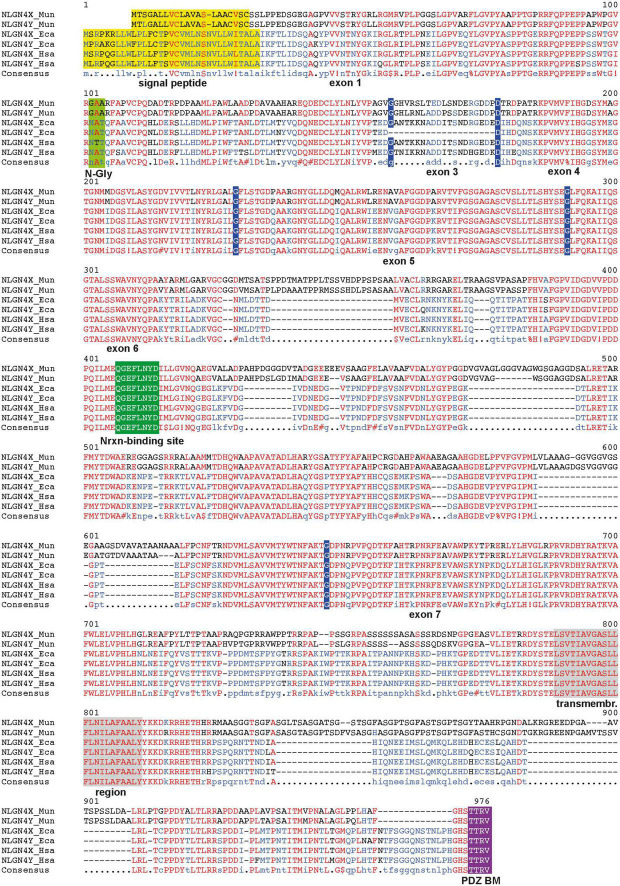
Alignment of NLGN4X and NLGN4Y protein sequences from different species. Key features of the three pairs of neuroligin-4 protein sequences are depicted: exon/exon junctions, indigo; signal peptide, yellow; critical N-glycosylation site (N-Gly) (Cast et al., 2021), lighter green; critical neurexin (Nrxn) binding site (Araç et al., 2007), green; PDZ binding motif (PDZ BM), purple; transmembrane region, gray. The respective name and number of each encoding exon is displayed below the protein sequence. The alignment highlights several insertions exclusively present in the Mongolian gerbil (Mun), that are absent in horse (Eca) and human sequences (Hsa). The alignment was performed using MultAlin (Corpet, 1988). Special characters in the line of the consensus sequence are placeholders for the following amino acids:!, isoleucine or valine; $, leucin or methionine;%, phenylalanine or tyrosine; #, aspartic acid, asparagine, glutamic acid or glutamine; red represents identical amino acids, blue the predominant amino acids within the total alignment, and black the relatively underrepresented amino acids within the total alignment.

The organization of the gerbil neuroligin-4 genes is characterized by a series of features that are also seen in neuroligin-4 genes of the clade eumuroida, a subset of the suborder myomorpha (Maxeiner et al., 2020). These include the accumulation of repetitive sequences in introns and exons, and a general increase of the total GC and GC3 content, i.e., the increase of GC in the third position of synonymously coding codons. Given the presence of *bona fide* splice donor and acceptor sites as well as the lack of preliminary stop codons or any frameshifts resulting in non-sense mutations, we conclude that both gerbil neuroligin-4 genes are likely transcribed and translated into proteins. Alignment of NLGN4X and NLGN4Y amino acid sequences (Supplementary Figure 1) showed none or rare amino acid differences in exons 1, 4, and 5, and more substantial differences in exons 3, 6, and 7 comparable to our previous findings (Maxeiner et al., 2020). Functionally important regions, such as the neurexin-binding site and the PDZ-domain binding site are identical. Alignment with the respective human and horse NLGN4X and NLGN4Y protein sequences indicates that the gametolog pair in *M. unguiculatus* likely arose independently (Figure 2).

### Neuroligin-4 Evolution in the Superorder Euarchontoglires

**FIGURE 3:**
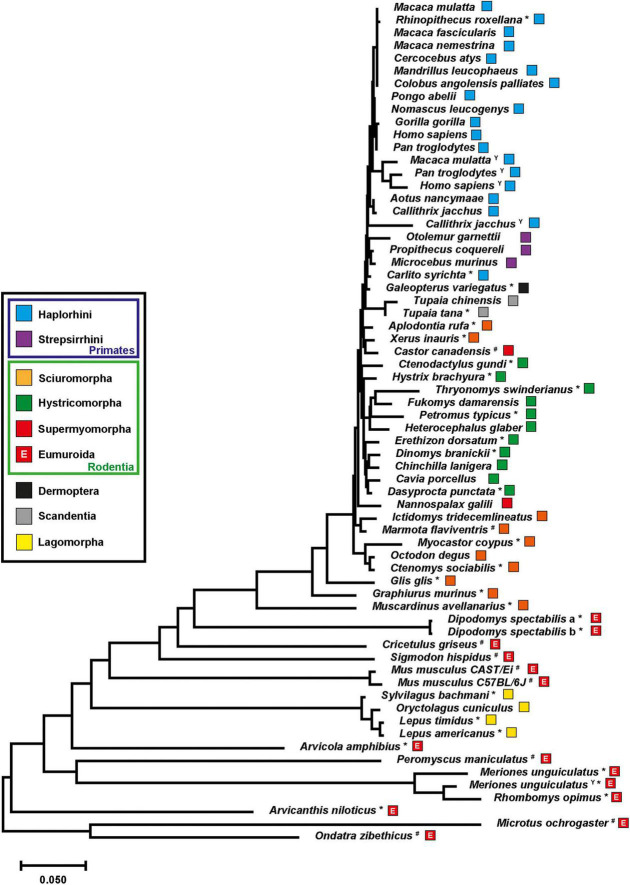
Evolution of neuroligin-4 within the superorder euarchontoglires. The evolutionary history of neuroligin-4 was inferred using the Neighbor-Joining method (Saitou and Nei, 1987). For this tree 66 neuroligin-4 protein sequences representing all five orders classified under the superorder euarchontoglires (Murphy et al., 2001), i.e., dermoptera, lagomorpha, primates, rodentia and scandentia, were aligned and the resulting optimal tree (branch length ≅ 3.29) is shown. Numbers of bootstrap replication (1,000) test are depicted in Supplementary Figure 3; Felsenstein, 1985). The tree is drawn to scale, with branch lengths in the same units as those of the evolutionary distances used to infer the phylogenetic tree. The evolutionary distances were computed using the Poisson correction method (Zuckerkandl and Pauling, 1965) and are in the units of the number of amino acid substitutions per site. The rate variation among sites was modeled with a gamma distribution (shape parameter = 1). All ambiguous positions were removed for each sequence pair (pairwise deletion option). Correction of the very C-terminal amino acid sequence was performed manually resulting in a total of 1,549 positions in the final dataset. Evolutionary analyses were conducted in MEGA X (Kumar et al., 2018; Stecher et al., 2020). Each species has been annotated with a color code facilitating the assignment to the main five orders. Primates have been further divided into two clades, i.e., haplorhini and strepsirrhini. Rodents were further separated into the three major suborders, hystricomorpha, sciuromorpha, and supermyomorpha (D’Elía et al., 2019). The addition of an upper-case “E” into the box representing the suborder supermyomorpha indicates that these species are part of the clade eumuroida (Steppan and Schenk, 2017). Asterisks are marking sequences that have been identified and added to this tree compared to our previous study (Maxeiner et al., 2020). A superscripted upper-case “Y” identifies sequences that have been annotated as NLGN4Y proteins. A formal assignment of the two neuroligin-4 protein sequences (“a” and “b”) in *Dipodomys spectabilis* as X- or Y-chromosomal copies was not possible.

The observation of neuroligin-4 divergence into gametologs in gerbils prompted us to revisit our previous assessment of the neuroligin-4 phylogeny (Maxeiner et al., 2020). Including new genome releases of rodent, primate, and lagomorph species as well as those of treeshrews and the colugo, we extended our analysis to the entire mammal superorder euarchontoglires (Murphy et al., 2001) and assessed the phylogeny of neuroligin-4 using a total of 66 sequences (Figure 3). Many of these were not fully annotated in the databases and needed to be inferred by sequence comparison (Supplementary Data Sheets 2, 4).

Further, we aimed at including at least one representative of each of the 35 rodent families. Rodents separate into three major branches, the “porcupine-like” hystricomorpha (17 families), the “squirrel-like” sciuromorpha (3 families), and the “mouse-like” supermyomorpha (15 families) (D’Elía et al., 2019), with the latter one being the largest as regards the number of different species. Supermyomorpha represent approximately 75% of all rodents (Burgin et al., 2018; Maxeiner et al., 2021). Primates, treeshrews (*Tupaia* spec.), the colugo (*G. variegatus*), all families within the suborder hystricomorpha and sciuromorpha, as well as *Nannospalax galili* and *Castor canadensis* from the suborder supermyomorpha display a very close evolutionary relationship as judged by exon sizes and phylogenetic analysis (Figure 3 and Supplementary Data Sheets 2, 4).

The sequences of *Arvicola amphibius*, *Arvicanthis niloticus* and gerbils (*M. unguiculatus* and *Rhombomys opimus*) confirmed the neuroligin-4 gene changes within the clade eumuroida described above, i.e., an increase in GC and GC3 content, an incorporation of repetitive sequences, and a reduction of the total gene size. Interestingly, though, we confirmed similar changes now also outside of the eumuroida clade (Supplementary Data Sheets 2, 5). This was, for instance, the case for the kangaroo rat, *Dipodomys spectabilis*, a member of the Heteromyidae family, which is closely related to beavers (Castoridae). The male kangaroo rat genome data also indicate the presence of two “neuroligin-4-like” genes (Supplementary Data Sheet 2). Both are remarkably similar, with only one amino acid difference (Supplementary Figure 2). Thus, the two isoforms are depicted as “a” and “b” variants as the sequences do not provide conclusive information about neighboring genes that would indicate an X- or Y-chromosomal location. In fact, both genes are identical in their 5 prime regions up to the intron following the exon carrying the presumptive start codon, indicating that *D. spectabilis* neuroligin-4 genes might be in the process of transitioning from pseudoautosomal to gametologous genes. Remarkably, exon 3 is also absent from both neuroligin-4 copies in *D. spectabilis*, reminiscent of some hamster species (Maxeiner et al., 2020).

**FIGURE 4:**
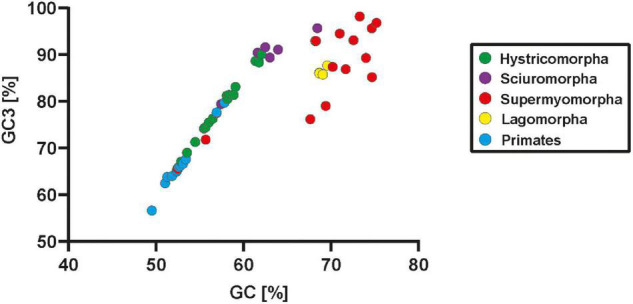
Changes in GC-content are a characteristic of supermyomorpha and lagomorpha. The general percentage of GC-content (X-axis) of the coding region as well as the percentage of the GC3 content (Y-axis), i.e., codons with GC in the third position, are displayed in an XY scatter blot. Each data point represents a single primate, rodent or lagomorph species that had already been used to infer the evolutionary tree in Figure 3. Primates and lagomorphs are represented in a single color, rodents have been further subdivided into their three suborders with individual colors. All primate and hystricomorpha species, most sciuromorpha species as well as the supermyomorpha species *C. canadensis* and *N. galili* follow a tight GC/GC3 relationship with a similar slope whether they encode *NLGN4X* or *NLGN4Y* genes. Some sciuromorpha, all lagomorphs, and most supermyomorpha species form a loose cloud with exceptionally high GC and GC3 content.

Lagomorphs represent an order that is most closely related to rodents. Analysis of their neuroligin-4 sequences revealed changes in the neuroligin-4 coding sequence that appear to have happened independently to those observed within rodents, or more specifically within supermyomorpha. Our analysis of four representatives from three different genera (*Oryctolagus*, *Sylvilagus* and *Lepus*) of lagomorphs reveal that they cluster tightly, but clearly apart from the group of highly similar neuroligin-4 orthologs present in primates and hystricomorpha (Figure 3, for branching statistics see Supplementary Figure 3). Gene and protein sequence analyses indicate that apart from their phylogenetic distance to rodents likewise changes as observed in supermyomorpha have emerged. These include an increase of the GC- and GC3-content, a reduction of the overall gene size, and an accumulation of repetitive sequences. These changes within the coding region are immediately evident by plotting the GC-and GC3-content for each species (Figure 4 and Supplementary Data Sheet 5). All investigated sequences from primates and hystricomorpha cluster in a linear fashion, with hystricomorpha species displaying a generally elevated GC- and GC3-content as compared to primates. The squirrel-like sciuromorpha show further an increase in their respective GC content, partially exiting the linear GC/GC3 relationship. Most of the supermyomorpha, however, excluding *C. canadensis* and *N. galili*, form a loose, non-linear cloud of data points with exceptionally high GC- and GC3-contents. This “supermyomorpha island” also harbors a tight cluster representing the four investigated lagomorph species.

### Independent NLGN4X/Y Diversification Processes During Mammalian Evolution

**FIGURE 5:**
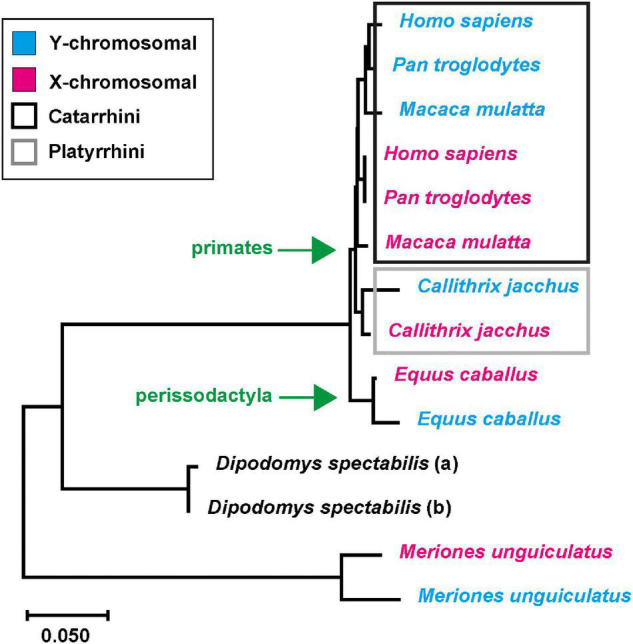
Independent neuroligin-4 diversification into gametologs. Six pairs of NLGN4X and NLGN4Y coding sequences as well as a pair of neuroligin-4 sequences from *D. spectabilis* were used to infer the evolutionary history using the Neighbor-Joining method (Saitou and Nei, 1987). Depicted is the optimal tree with the sum of branch length ≅ 0.649. Statistics of bootstrap replicates can be found in Supplementary Figure 4. The tree is drawn to scale. The evolutionary distances were computed using the Maximum Composite Likelihood method (Tamura et al., 2004) and are in the units of the number of base substitutions per site. The rate variation among sites was modeled with a gamma distribution (shape parameter = 1). The differences in the composition bias among sequences were considered in evolutionary comparisons (Tamura and Kumar, 2002). All ambiguous positions were removed for each sequence pair (pairwise deletion option). There were a total of 1,008 positions in the final dataset. Evolutionary analyses were conducted in MEGA X (Kumar et al., 2018; Stecher et al., 2020). Diversification in X- and Y-specific neuroligin-4 copies occurred at least twice and independently within primates and rodents, respectively, as well as in the evolution of horses in the order perissodactyla.

Many genome releases derive from female source DNA, which yields limited insights into the actual size of the pseudoautosomal region and its boundary to X-specific genes since a direct comparison to the sequence of the Y-chromosome is missing. However, this information is necessary to determine whether there might be a potential diversification of neuroligin-4 into gametologs. Interestingly, very few genome releases are from males, and a respective *NLGN4Y* gametolog can been identified. This is the case for some primates from the clade catarrhini, including humans, rhesus macaque (*Macaca mulatta*), and chimpanzee (*Pan troglodytes*), and the clade platyrrhini with the common marmoset (*Callithrix jacchus*). Beyond this, *NLGN4X/Y* gametologs were identified in horses (*Equus caballus*), gerbils (*M. unguiculatus*, this paper), and *D. spectabilis* (this paper) (for a list of the coding sequences, see Supplementary Data Sheet 6). Given the limited number of species’ genomes from male specimen, it appears feasible to assume that in an early stage of mammalian evolution *NLGN4X* and *NLGN4Y* developed as gametologs, like *AMELX/Y*, and that *NLGN4Y* still needs to be identified in the corresponding male samples. This, in turn, would mean that all sequenced *NLGN4Y* orthologs should have a single common ancestor. To scrutinize this notion and to increase the resolution of our analysis, we used the underlying protein coding nucleotide sequences and not the amino acid sequences to infer an evolutionary tree for given *NLGN4X* and *NLGN4Y* pairs of six different species and the gene variants in *D. spectabilis* (Figure 5 and Supplementary Data Sheet 6; for branching statistics cf. Supplementary Figure 4). Strikingly, the corresponding tree indicates five independent incidences of neuroligin-4 diversification into gametologs. Within the primate order, the *NLGN4X/Y* diversification occurred within the clades catarrhini and platyrrhini independently. A comparable scenario occurred in rodents (gerbils and *D. spectabilis*), and an additional independent separation happened during the evolution of horses in the order perissodactyla.

### Pseudoautosomal Regions in Gerbils

**FIGURE 6:**
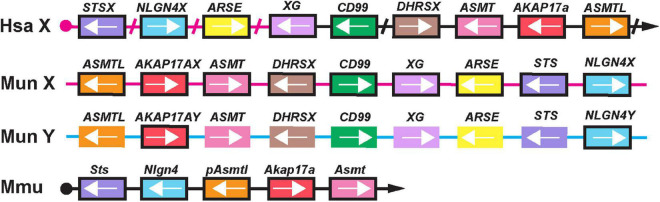
X- and Y-chromosomal sequences display loss of their pseudoautosomal character in gerbils. A schematic illustrates the presence of those genes that have been identified to comprise the genes of the pseudoautosomal region in the laboratory mouse (Mmu, Maxeiner et al., 2020), genes present in the vicinity of *NLGN4X* and *NLGN4Y* in gerbils (Mun X and Mun Y), as well as the homologs on the X-specific and pseudoautosomal region on the human X chromosome (Hsa X). The magenta line indicates that *STSX*, *NLGN4X*, *ARSE* and parts of *XG* are located on the X-specific part of the human X-chromosome, all other genes are pseudoautosomal. Arrowheads direct toward the telomeres. Slashes are marking gaps in the human sequence comprising genes irrelevant for this comparison. In laboratory mice, six genes are localized to the pseudoautosomal region. The *Erdr1* gene upstream of *Sts* is not shown since it is only present in mice. Comparison of human and mouse genes with those present on the gerbil X and Y chromosomes reveal that both neuroligin-4 gametologs share a similar vicinity. The gerbil Y-chromosomal sequence retains *NLGN4Y* and *AKAP17AY* as likely functional genes. The remaining genes display various mutations and have become subject to pseudogenization. Pseudogenes are presented in unframed boxes; framed boxes resemble genes with potential expression based on the presence of an intact reading frame. The results are based on sequence comparison between VFHZ01026354.1 (Mun X) and VFHZ01013942.1 (Mun Y).

Given the close evolutionary relationship between gerbils and mice, we hypothesized that some of the PAR genes conserved in mice (i.e., *Sts*, *Asmt* or *Akap17a*, Maxeiner et al., 2020) might still be present in the close vicinity of the neurolgin-4 gene orthologs in gerbils. Indeed, the contigs (VFHZ01026354.1[X-chr.], VFHZ01013942.1[Y-chr.]) with the sequence information of *NLGN4X* and *NLGN4Y* contained *ASMT*, *AKAP17A*, *ASMT*, *DHRSX*, *CD99*, *XG*, *ARSE*, and *STS* within a stretch of approx. 80 kilobases, respectively (Figure 6). However, these genes appeared to be conserved as potentially functional proteins only on the contig that also contained *NLGN4X*. On the corresponding contig with *NLGN4Y*, solely *AKAP17A* appears to be translatable into a functional protein (“*AKAP17AY*”). Incomplete sequence information from a sister species of the mangolian gerbil, the fat sand rat *Psammomys obesus*, indicates that a contig (NESX01053770.1) with the neuroligin-4 gene ends downstream of exon 7 with the repetition of a telomere motif. Thus, gerbils might not retain any PAR-like homology among the remaining ancestral autosomal genes, possibly explaining the observation that synaptonemal complexes do not occur during male meiosis (De la Fuente et al., 2007).

## Discussion

Our study provides new insights into the evolution of the ASD-associated gene neuroligin-4 in euarchontoglires, a superorder encompassing the mammalian orders most closely related to humans, such as rodents and lagomorphs (Murphy et al., 2001). Our study complements our recent findings regarding the evolution of neuroligin-4, which showed that remarkable differences in the neuroligin-4 genes of laboratory mice as compared to the human gametologs *NLGN4X* and *NLGN4Y* are the consequence of general changes in an eroding pseudoautosomal region of both sex chromosomes in the clade eumuroida (Maxeiner et al., 2020). These changes include, (a) a dramatic reduction of the neuroligin-4 gene size, (b) an increase of G and C bases in the coding region, (c) an increase of the abundance of G and C in the third position of codons (GC3), (d) an accumulation of tandem repeats in introns, and (e) repetitive sequences in the coding sequence (Maxeiner et al., 2020).

Previous studies demonstrated that the diversification of neuroligin-4 from an originally pseudoautosomal gene into a pair of gametologs occurred in humans and other hominid primates (Cortez et al., 2014) as well as in horses (Raudsepp and Chowdhary, 2015), due to shifts of the boundary separating the pseudoautosomal region from X-specific regions on the X-chromosome and male-specific regions on the Y-chromosome. Due to the lack of information on male rodent genomes, more general conclusions regarding the diversification of neuroligin-4 into X- and Y-specific gametologs had not been possible.

The present study was triggered by sequence information from a male gerbil genome, indicating the presence of two “neuroligin-4-like” sequences. We experimentally completed the corresponding genomic neuroligin-4 sequences and found that both genes display characteristics of true gametologs, i.e., one copy was present only in females, therefore, referred to as *NLGN4X*, and one additionally present in males, referred to as *NLGN4Y*. Both genes share all the characteristics that were identified in neuroligin-4 orthologs in eumuroida, indicating that the diversification into gametologs arose evolutionarily recently while the PAR had already started to erode.

Our initial assessment of neuroligin-4 evolution indicated events exclusive to a subset of the suborder myomorpha, the clade eumuroida. We now extended our analysis to all available rodent families (D’Elía et al., 2019), additional primate sequences, and genome releases from scandentia, dermoptera, and lagomorpha species, and can generate a much more refined description of neuroligin-4 in euarchontoglires.

The “porcupine-like” suborder hystricomorpha and the “squirrel-like” suborder sciuromorpha, together comprising a quarter of all rodent species (Burgin et al., 2018; Maxeiner et al., 2021), show a genomic organization of the neuroligin-4 gene that tightly matches that of humans (i.e., its stretch over the genome, its consistent exon sizes, and its moderate GC/GC3 ratio). This is different in the suborder supermyomorpha, with exception of the beavers (*C. canadensis*) or moles of the family Spalacidae (e.g., *N. galili*). Our previous conclusion that solely eumuroida species as a subset of supermyomorpha display the mentioned gene changes needs now to be revised and to be extended to other supermyomorpha species such as, for instance, *D. spectabilis*, a species from the family Heteromyidae, which is not a member of eumuroida [family classification can be found in D’Elía et al., 2019; eumuroida classification in Steppan and Schenk (2017)]. Further, lagomorpha species, i.e., rabbits and hares that do not belong to the rodent order, show changes to neuroligin-4 reminiscent of those found in supermyomorpha, i.e., (a) a dramatic reduction of gene size, (b) repetitive sequences in the introns, and (c) an increase in the GC- and GC3-content. Since lagomorphs represent an independent mammalian order on equal terms with rodents and primates, this observation marks the first encounter of substantial changes to neuroligin-4 genes outside the rodent order.

The boundary between the PARs and sex chromosome-specific regions differs in mammals of different orders (Raudsepp and Chowdhary, 2015). This prompted us to investigate whether the neuroligin-4 gametologs in different species have a common origin or developed independently upon the shift of the pseudoautosomal boundary. Using a total of seven different sets of neuroligin-4 gametologs we were able to confirm that the diversification into gametologs occurred independently at least five times: twice in rodents (*D. spectabilis* and *M. unguiculatus*), twice in primates (*C. jacchus* and the hominids), and once in odd-toed ungulates (horses).

Integrating our previous observations (Maxeiner et al., 2020) and the present results, we can conclude the following regarding the evolution of neuroligin-4: (a) Neuroligin-4 is either pseudoautosomal (e.g., laboratory mice, Maxeiner et al., 2020) or diverged into gametologs (e.g., gerbils, humans, and horses, this manuscript; Raudsepp and Chowdhary, 2015). (b) Neuroligin-4 is subject to genomic changes that ultimately affect its coding sequence; these changes include general GC-content and GC3-content, insertions or deletions, repetitive sequences in introns, and the reduction of the overall gene size. (c) These genomic changes occurred independently in different rodents and lagomorphs. (d) The changes affecting the number of exons also occurred independently, e.g., in horse *NLGN4Y*, *D. spectabilis NLGN4* gene variants “a” and “b,” and in some hamster species (*Ondatra zibethicus*, *Cricetulus griseus*) exon 3 is absent.

The evolution of neuroligin-4 poses a fascinating puzzle as compared to other genes in its close vicinity that are present in the X-specific region or PAR of the human X-chromosome. Its diversification into an X- and into a Y-specific copy marks a rare incidence that only occurred in very few other genes. The accumulation and potential tolerance of additional sequences in supermyomorpha species appears to fuel an accelerated evolution of the neuroligin-4 gene to counteract a progressive PAR erosion. Human *NLGN4X* and many genes in its vicinity have clinical relevance, and several were lost during rodent evolution, e.g., *MXRA5*, *SHOX*, and *ANOS1* (Maxeiner et al., 2021). This indicates an evolutionary advantage to retain neuroligin-4 in general, as well as a second copy on the Y-chromosome. This might in fact be the very reason why in gerbils only *NLGN4Y* (and perhaps *AKAP17AY*) “survived” on the Y-chromosomes as remnants of an ancestral PAR gene cluster.

Our attempt to identify the *NLGN4X* and *NLGN4Y*-specific copies based on a length polymorphism suggests a possible implementation of the *NLGN4X/Y* loci to establish a sex-typing strategy specific for the Mongolian gerbil as has recently been developed for humans and horses (Maxeiner et al., 2019; Zaffalon et al., 2019). Given that gametologs are only present on the respective sex chromosome, PCR-based fragment analysis of gametologs allows to infer the presence of the X- and Y- chromosomes in samples. Compared to merely determining the presence of the *SRY* gene as a “male marker,” tests for gametologs always incorporate a positive control as the X-specific gametolog is amplified from both, XY and XX sample DNAs. The gametologous gene pair *AMELX/Y*, for instance, has long been employed in forensic genetics for this purpose, e.g., in humans, dogs, horses, and pigs (Mannucci et al., 1994; Hasegaw et al., 2000; Fontanesi et al., 2008; Yan et al., 2013). Employing neuroligin-4 gametologs resembles an alternative method with a new gene pair, thus expanding the test portfolio in forensic genetics or animal breeding when the biological sex is relevant.

The evolutionary demand to select for and maintain a Y-chromosomal copy of neuroligin-4 is still not understood and remains unclear. Earlier studies on the amelogenin gene pair, *AMELX* and *AMELY*, showed that the *AMELY* expression level is just about a tenth of *AMELX* (Salido et al., 1992). It is unclear whether a similar scenario arises with *NLGN4X* and *NLGN4Y*, and thorough comparative analyses of expression levels, promoter activities, expression patterns, and cellular functions are required to answer this question. Specific antibodies need to be generated to study protein expression and its (sub-) cellular localization *in situ*. Foremost, the plethora of neuroligin-4 variants deviating from its conserved form in primates and some rodents (such as within the hystricomorpha), need to be placed into the databases warranting to be found and matched by algorithms identifying sequencing results of RNAseq studies. In this regard, the present data indicate that the gerbil might be an adequate model to address these issues, particularly given that accessibility to human and horse tissue is limited.

The evolution of the neuroligin-4 gene in the superorder euarchontoglires represents a fascinating phenomenon for neuroscientists and geneticists alike. Its resistance to the continuous and relentless process of PAR erosion in the species’ richest mammal order, its diversification into X- and Y-specific copies as a result to adapt to the inevitable telomeric shift of PAR boundaries, and its tolerance to erosion-induced changes in its coding region are setting it apart from essentially all other neuronal cell adhesion proteins. We suggest that gerbils might be a suitable rodent research model to study key functional aspects of neuroligin-4’s striking exceptionality.

## Data Availability Statement

The datasets presented in this study can be found in online repositories. The names of the repository/repositories and accession number(s) can be found in the article/Supplementary Material.

## Ethics Statement

The animal study was reviewed and approved by Ethics Committee of the Medical Faculty of Saarland University.

## Author Contributions

SM conceived the study and wrote the manuscript. SM and FB performed the experiments. NB and GK-C provided the reagents. SM, NB, and GK-C edited the manuscript. All authors contributed to the article and approved the submitted version.

## Conflict of Interest

The authors declare that the research was conducted in the absence of any commercial or financial relationships that could be construed as a potential conflict of interest.

## Publisher’s Note

All claims expressed in this article are solely those of the authors and do not necessarily represent those of their affiliated organizations, or those of the publisher, the editors and the reviewers. Any product that may be evaluated in this article, or claim that may be made by its manufacturer, is not guaranteed or endorsed by the publisher.

## Footnotes

1
https://blast.ncbi.nlm.nih.gov/Blast.cgi

2
https://multalin.toulouse.inra.fr/multalin/

3
https://www.ebi.ac.uk/Tools/msa/clustalo/

4
https://services.healthtech.dtu.dk/service.php?SignalP-5.0

5
https://www.endmemo.com/bio/gc.php

6
https://gcua.schoedl.de

7
https://www.biologicscorp.com/tools/GCContent/index

8
https://web.expasy.org/translate/
