# Supplementary material for: Of Humans and Gerbils— Independent Diversification of Neuroligin-4 Into X- and Y-Specific Genes in Primates and Rodents
Source: Front Mol Neurosci. 2022 Mar 30;15:838262. doi: 10.3389/fnmol.2022.838262 (PMC9005811; doi:10.3389/fnmol.2022.838262)
Supplement: Supplementary file 3 [file Data_Sheet_3.docx]

Supplementary Text File 1

The depicted alignment derives from a comparison of the NLGN4X and NLGN4Y exon 6 sequences from the Mongolian gerbil (Mun). Within exon 6 a length polymorphism was identified that served as a basis for a sex-typing strategy to infer the biological sex. The flanking oligonucleotides that serve as a primer pair are depicted with an underscore.

1 80

NLGN4X_Mun GCCTGTTCCAGAAGGCCATCATCCAGAGCGGCACCGCACTGTCCAGCTGGGCTGTCAACTACCAGCCGGCGGCGTATGCG

NLGN4Y_Mun GCCTGTTCCAGAAGGCCATCATCCAGAGCGGCACCGCACTGTCCAGCTGGGCTGTCAACTACCAGCCGGCGGTGTATGCA

81 160

NLGN4X_Mun CGCATGCTCGGAGCCCGTGTAGGCTGCGGAGGAGACATGACGTCGGCGACCTCGCCCCCGGACACCATGGCGACGCCTCC

NLGN4Y_Mun CGCATGCTCGGGGCCCGTGTGGGCTGCGGGGGAGACGTGATGTCGGCGACCCCGCTCCCGGACGCTGCGGCGACGCCACC

161 240

NLGN4X_Mun CTTGACGTCCTCGGTGCACGACCCGCCCTCCCCGTCAGCTGCGCTAGTGGCCTGCCTCCGCCGCCGAGGCGCCCGCGAGC

NLGN4Y_Mun CCGGATGTCCTCGTCTCACGATCTGCCTTCCGCATCAGCTGCGCTAGTGGCCTGCCTCCGTCGCCGTGGGGCCCGCGAGC

241 320

NLGN4X_Mun TGACCCGGGCTGCGGGTTCGGTACCCGCGTCCGCGCCCTTCCACGTGGCCTTCGGGCCAGTGATCGATGGTGATGTGGTG

NLGN4Y_Mun TGACCCGGGCCGCGGGTTCGGTGCCCGCGTCCTCACCATTCCATGTGGCCTTCGGGCCAGTGATCGATGGAGATGTGGTG

321 400

NLGN4X_Mun CCTGACGACCCGCAGATCCTCATGGAGCAGGGTGAGTTCCTCAACTACGACATCCTTCTGGGCGTCAACCAGGCGGAGGG

NLGN4Y_Mun CCGGACGACCCGCAGATCCTCATGGAGCAGGGTGAGTTCCTCAACTACGACATCCTTCTGGGCGTCAACCAGGCGGAGGG

401 480

NLGN4X_Mun CGTGGCCCTGGCCGACCCCGCCCACCCGGACGGCGGCGGCGACGTCACAGCGGATGGCGAAGAGGAGGAGGAGGTGTCGG

NLGN4Y_Mun CGTGGCCCTGGCAGACCCCGCCCACCCCGACAGCCTCGGGGATATAATGGCTGACGGTGA------GGAGGAGGTGTCGG

481 560

NLGN4X_Mun CTGCAGGCTTCGAACTCGCTGTTGCCGCCTTCGTGGATGCGCTGTACGGCTACCCGGGAGGGGATGTGGGCGTGGCCGGA

NLGN4Y_Mun CTGCCGGCTTCGAACTCGCTGTTGCTGCCTTCGTGGATGCGCTGTACGGCTACCCAGGAGGGGATGTGGGCGTGGCCGG-

561 640

NLGN4X_Mun CTGGGCGGGGGCGTGGCCGGCTGGGGCAGTGGAGCTGGCGGGGACTCGGCCCTTCGCGAGACGGCGCGCTTCATGTACAC

NLGN4Y_Mun --------------------CTGGAGCGGTGGAGCCGGCGGGGACTCCGCCCTTCGCGAGACGGCGCGCTTCATGTACAC

641 720

NLGN4X_Mun GGACTGGGCGGAGCGCGAGGGCGGGGCGGGGTCACGGCGCCGCGCCCTGGCGGCCATGATGACGGACCACCAGTGGGCGG

NLGN4Y_Mun GGACTGGGCGGAGCGCGAGGGCGGGGCGGGGTCACGGCGCCGCGCCTTGGCGGCCATGATGACGGACCACCAGTGGGCGG

721 800

NLGN4X_Mun CGCCCGCCGTGGCCACCGCGGACTTGCACGCCCGGTACGGCTCGGCCACCTACTTCTACGCCTTTGCACACCCGTGTCGG

NLGN4Y_Mun CGCCCGCCGTGGCCACGGCCGACTTGCACGCCCGGTACGGCTCGGCCACCTACTTCTACGCCTTTGCACACCCGTGTCGG

801 880

NLGN4X_Mun GGGGACGCGCACCCCGCCTGGGCGGCCGAAGCGGGCGCGGCTCATGGTGACGAGCTGCCCTTCGTGTTCGGGGTCCCGAT

NLGN4Y_Mun GGGGACGCGCACCCCGCCTGGGCGGCCGAAGCGGGCGCTGCCCATGGCGACGAGCTGCCCTTCGTATTCGGGGTCCCGAT

881 960

NLGN4X_Mun GCTCGTGCTGGCGGCGGCCGGGGGTGGG---GTTGGAGGAGTCGGAAGCGAGGGCGCGGCCGGAAGTGACGTCGCCGTGG

NLGN4Y_Mun GCTCGTGCTGGCGGCGGCCGGTGATGGCAGTGTCGGAGGAGTCGGAGGCGAAGGCGCAACGGGAACTGATGTCGCTGCGG

961 1040

NLGN4X_Mun CCACCGCCGCCAATGCTGCCGCCCTCTTCCCGTGCAACTTCACGCGTAATGACGTAATGCTCAGCGCCGTCGTCATGACG

NLGN4Y_Mun CCACCGCCGCT---------GCCCTCTTTCCATGCAACTTCACGCGCAATGACGTGATGCTCAGCGCCGTCGTCATGACG

1041 1066

NLGN4X_Mun TACTGGACCAACTTCGCCAAGACGGG

NLGN4Y_Mun TACTGGACCAACTTCGCCAAGACCGG
