# Supplementary material for: Of Humans and Gerbils— Independent Diversification of Neuroligin-4 Into X- and Y-Specific Genes in Primates and Rodents
Source: Front Mol Neurosci. 2022 Mar 30;15:838262. doi: 10.3389/fnmol.2022.838262 (PMC9005811; doi:10.3389/fnmol.2022.838262)
Supplement: Supplementary file 4 [file Data_Sheet_4.docx]

Supplementary Text File 2

The protein sequences below (FASTA format) served as the basis for the generation of the phylogenetic tree in Figure 3. All sequence references are additional summarized in Suppl Data Sheet 2.

>Mandrillus_leucophaeus_XP_011827489.1

MSRPQGLLWLPLLFTPVCVMLNSNVLLWITALAIKFTLIDSQAQYPVVNTNYGKIRGLKTPLPSEILGPVEQYLGVPYASPPTGERRFQPPEPPSSWTGIRNTTQFAAVCPQHLDERSLLHDMLPIWFTANLDTLMTYVQDQNEDCLYLNIYVPTEDGANTKKNADDITSNDRGEDEDIHDQNSKKPVMVYIHGGSYMEGTGNMIDGSILASYGNVIVITINYRLGILGFLSTGDQAAKGNYGLLDQIQALRWIEENVGAFGGDPKRVTIFGSGAGASCVSLLTLSHYSEGLFQKAIIQSGTALSSWAVNYQPAKYTRILADKVGCNMLDTTDMVECLRNKNYKELIQQTITPATYHIAFGPVIDGDVIPDDPQILMEQGEFLNYDIMLGVNQGEGLKFVDGIVDNEDGVTPNDFDFSVSNFVDNLYGYPEGKDTLRETIKFMYTDWADKENPETRRKTLVALFTDHQWVAPAVATADLHAQYGSPTYFYAFYHHCQSEMKPSWADSAHGDEVPYVFGIPMVGPTELFSCNFSKNDVMLSAVVMTYWTNFAKTGDPNQPVPQDTKFIHTKPNRFEEVAWSKYNPKDQLYLHIGLKPRVRDHYRATKVAFWLELVPHLHNLNEIFQYVSTTTKVPPPDMTSFPYGTRRSPAKIWPTTKRPAITPANNPKHSKDPHKTGPEDTTVLIETKRDYSTELSVTIAVGASLLFLNILAFAALYYKKDKRRHETHRRPSPQRNTTNDIAHIQNEEIMSLQMKQLEHDHECESLQAHDTLRLTCPPDYTLTLRRSPDDIPLMTPNTITMIPNTLTGMQPLHTFNTFSGGQNSTNLPHGHSTTRV

>Colobus_angolensis_palliates_XP_011812892.1

MSRPQGLLWLPLLFTPVCVMLNSNVLLWITALAIKFTLIDSQAQYPVVNTNYGKIRGLKTPLPSEILGPVEQYLGVPYASPPTGERRFQPPEPPSSWTGIRNTTQFAAVCPQHLDERSLLHDMLPIWFTANLDTLMTYVQDQNEDCLYLNIYVPTEDGANTKKNADDITSNDRGEDEDIHDQNSKKPVMVYIHGGSYMEGTGNMIDGSILASYGNVIVITINYRLGILGFLSTGDQAAKGNYGLLDQIQALRWIEENVGAFGGDPKRVTIFGSGAGASCVSLLTLSHYSEGLFQKAIIQSGTALSSWAVNYQPAKYTRILADKVGCNMLDTTDMVECLRNKNYKELIQQTITPATYHIAFGPVIDGDVIPDDPQILMEQGEFLNYDIMLGVNQGEGLKFVDGIVDNEDGVTPNDFDFSVSNFVDNLYGYPEGKDTLRETIKFMYTDWADKENPETRRKTLVALFTDHQWVAPAVATADLHAQYGSPTYFYAFYHHCQSEMKPSWADSAHGDEVPYVFGIPMVGPTELFSCNFSKNDVMLSAVVMTYWTNFAKTGDPNQPVPQDTKFIHTKPNRFEEVAWSKYNPKDQLYLHIGLKPRVRDHYRATKVAFWLELVPHLHNLNEIFQYVSTTTKVPPPDMTSFPYGTRRSPAKIWPTTKRPAITPANNPKHSKDPHKTGPEDTTVLIETKRDYSTELSVTIAVGASLLFLNILAFAALYYKKDKRRHETHRRPSPQRNTTNDIAHIQNEEIMSLQMKQLEHDHECESLQAHDTLRLTCPPDYTLTLRRSPDDIPLMTPNTITMIPNTLTGMQPLHTFNTFSGGQNSTNLPHGHSTTRV

>Pongo_abelii_XP_024096638.1_plus_insert_A2

MSRPQGLLWLPLLFTPVCVMLNSNVLLWITALAIKFTLIDSQAQYPVVNTNYGKIRGLRTPLPNEILGPVEQYLGVPYASPPTGERRFQPPEPPSSWTGIRNTTQFAAVCPQHLDERSLLHDMLPIWFTANLDTLMTYVQDQNEDCLYLNIYVPTEDGANTKKNADDITSNDRGEDEDIHDQNSKKPVMVYIHGGSYMEGTGNMIDGSILASYGNVIVITINYRLGILGFLSTGDQAAKGNYGLLDQIQALRWIEENVGAFGGDPKRVTIFGSGAGASCVSLLTLSHYSEGLFQKAIIQSGTALSSWAVNYQPAKYTRILADKVGCNMLDTTDMVECLRNKNYKELIQQTITPATYHIAFGPVIDGDVIPDDPQILMEQGEFLNYDIMLGVNQGEGLKFVDGIVDNEDGVTPNDFDFSVSNFVDNLYGYPEGKDTLRETIKFMYTDWADKENPETRRKTLVALFTDHQWVAPAVATADLHAQYGSPTYFYAFYHHCQSEMKPSWADSAHGDEVPYVFGIPMIGPTELFSCNFSKNDVMLSAVVMTYWTNFAKTGDPNQPVPQDTKFIHTKPNRFEEVAWSKYNPKDQLYLHIGLKPRVRDHYRATKVAFWLELVPHLHNLNEIFQYVSTTTKVPPPDMTSFPYGTRRSPAKIWPTTKRPAITPANNPKHSKDPHKTGPEDTTVLIETKRDYSTELSVTIAVGASLLFLNILAFAALYYKKDKRRHETHRRPSPQRNTTNDIAHIQNEEIMSLQMKQLEHDHECESLQAHDTLRLTCPPDYTLTLRRSPDDIPLMTPNTITMIPNTLTGMQPLHTFNTFSGGQNSTNLPHGHSTTRV

>Cercocebus_atys_XP_011891391.1

MSRPQGLLWLPLLFTPVCVMLNSNVLLWITALAIKFTLIDSQAQYPVVNTNYGKIRGLKTPLPSEILGPVEQYLGVPYASPPTGERRFQPPEPPSSWTGIRNTTQFAAVCPQHLDERSLLHDMLPIWFTANLDTLMTYVQDQNEDCLYLNIYVPTEDGANTKKNADDITSNDRGEDEDIHDQNSKKPVMVYIHGGSYMEGTGNMIDGSILASYGNVIVITINYRLGILGFLSTGDQAAKGNYGLLDQIQALRWIEENVGAFGGDPKRVTIFGSGAGASCVSLLTLSHYSEGLFQKAIIQSGTALSSWAVNYQPAKYTRILADKVGCNMLDTTDMVECLRNKNYKELIQQTITPATYHIAFGPVIDGDVIPDDPQILMEQGEFLNYDIMLGVNQGEGLKFVDGIVDNEDGVTPNDFDFSVSNFVDNLYGYPEGKDTLRETIKFMYTDWADKENPETRRKTLVALFTDHQWVAPAVATADLHAQYGSPTYFYAFYHHCQSEMKPSWADSAHGDEVPYVFGIPMVGPTELFSCNFSKNDVMLSAVVMTYWTNFAKTGDPNQPVPQDTKFIHTKPNRFEEVAWSKYNPKDQLYLHIGLKPRVRDHYRATKVAFWLELVPHLHNLNEIFQYVSTTTKVPPPDMTSFPYGTRRSPAKIWPTTKRPAITPANNPKHSKDPHKTGPEDTTVLIETKRDYSTELSVTIAVGASLLFLNILAFAALYYKKDKRRHETHRRPSPQRNTTNDIAHIQNEEIMSLQMKQLEHDHECESLQAHDTLRLTCPPDYTLTLRRSPDDIPLMTPNTITMIPNTLTGMQPLHTFNTFSGGQNSTNLPHGHSTTRV

>Macaca_nemestrina_XP_011733351.1_plus_insert_A2

MSRPQGLLWLPLLFTPVCVMLNSNVLLWITALAIKFTLIDSQAQYPVVNTNYGKIRGLKTPLPSEILGPVEQYLGVPYASPPTGERRFQPPEPPSSWTGIRNTTQFAAVCPQHLDERSLLHDMLPIWFTANLDTLMTYVQDQNEDCLYLNIYVPTEDGANTKKNADDITSNDRGEDEDIHDQNSKKPVMVYIHGGSYMEGTGNMIDGSILASYGNVIVITINYRLGILGFLSTGDQAAKGNYGLLDQIQALRWIEENVGAFGGDPKRVTIFGSGAGASCVSLLTLSHYSEGLFQKAIIQSGTALSSWAVNYQPAKYTRILADKVGCNMLDTTDMVECLRNKNYKELIQQTITPATYHIAFGPVIDGDVIPDDPQILMEQGEFLNYDIMLGVNQGEGLKFVDGIVDNEDGVTPNDFDFSVSNFVDNLYGYPEGKDTLRETIKFMYTDWADKENPETRRKTLVALFTDHQWVAPAVATADLHAQYGSPTYFYAFYHHCQSEMKPSWADSAHGDEVPYVFGIPMVGPTELFSCNFSKNDVMLSAVVMTYWTNFAKTGDPNQPVPQDTKFIHTKPNRFEEVAWSKYNPKDQLYLHIGLKPRVRDHYRATKVAFWLELVPHLHNLNEIFQYVSTTTKVPPPDMTSFPYGTRRSPAKIWPTTKRPAITPANNPKHSKDPHKTGPEDTTVLIETKRDYSTELSVTIAVGASLLFLNILAFAALYYKKDKRRHETHRRPSPQRNTTNDIAHIQNEEIMSLQMKQLEHDHECESLQAHDTLRLTCPPDYTLTLRRSPDDIPLMTPNTITMIPNTLTGMQPLHTFNTFSGGQNSTNLPHGHSTTRV

>Nomascus_leucogenys_XP_012356423.1

MSRPQGLLWLPLLFTPVCVMLNSNVLLWITALAIKFTLIDSQAQYPVVNTNYGKIRGLRTPLPNEILGPVEQYLGVPYASPPTGERRFQPPEPPSSWTGIRNTTQFAAVCPQHLDERSLLHDMLPIWFTANLDTLMTYVQDQNEDCLYLNIYVPTEDGANTKKNADDITSNDRGEDEDIHDQNSKKPVMVYIHGGSYMEGTGNMIDGSILASYGNVIVITINYRLGILGFLSTGDQAAKGNYGLLDQIQALRWIEENVGAFGGDPKRVTIFGSGAGASCVSLLTLSHYSEGLFQKAIIQSGTALSSWAVNYQPAKYTRILADKVGCNMLDTTDMVECLRNKNYKELIQQTITPATYHIAFGPVIDGDVIPDDPQILMEQGEFLNYDIMLGVNQGEGLKFVDGIVDNEDGVTPNDFDFSVSNFVDNLYGYPEGKDTLRETIKFMYTDWADKENPETRRKTLVALFTDHQWVAPAVATADLHAQYGSPTYFYAFYHHCQSEMKPSWADSAHGDEVPYVFGIPMIGPTELFSCNFSKNDVMLSAVVMTYWTNFAKTGDPNQPVPQDTKFIHTKPNRFEEVAWSKYNPKDQLYLHIGLKPRVRDHYRATKVAFWLELVPHLHNLNEIFQYVSTTTKVPPPDMTSFPYGTRRSPAKIWPTTKRPAITPANNPKHSKDPHKTGPEDTTVLIETKRDYSTELSVTIAVGASLLFLNILAFAALYYKKDKRRHETHRRPSPQRNTTNDIAHIQNEEIMSLQMKQLEHDHECESLQAHDTLRLTCPPDYTLTLRRSPDDIPLMTPNTITMIPNTLTGMQPLHTFNTFSGGQNSTNLPHGHSTTRV

>Propithecus_coquereli_XP_012512262.1

MSSPRGLLWLPLFFTPVCIMLNSNVLLWITALAIKFTLIDSQAQYPVVNTNYGKIRGLRTPLPNEILGPVEQYLGVPYASPPTGERRFQPPEPPSSWTGVRNATQFAAVCPQHLDERSLLHDMLPIWFTANLDTLMTYVQDQNEDCLYLNIYVPTEDGGNTKKNADDITSNDRGEDEDIHDQNSKKPVMVYIHGGSYMEGTGNMIDGSILASYGNVIVITINYRLGILGFLSTGDQAAKGNYGLLDQIQALRWIEENVGAFGGDPKRVTIFGSGAGASCVSLLTLSHYSEGLFQKAIIQSGTALSSWAVNYQPAKYTRILADKVGCNMLDTTDMVECLRNKNYKELIQQTITPATYHIAFGPVIDGDVIPDDPQILMEQGEFLNYDIMLGVNQGEGLKFVDGIVDNEDGVTPNDFDFSVSNFVDNLYGYPEGKDTLRETIKFMYTDWADKENPETRRKTLVALFTDHQWVAPAVATADLHAQYGSPTYFYAFYHHCQSEMKPSWADSAHGDEVPYVFGIPMIGPTELFSCNFSKNDVMLSAVVMTYWTNFAKTGDPNQPVPQDTKFIHTKPNRFEEVAWSKYNPKDQLYLHIGLKPRVRDHYRATKVAFWLELVPHLHNLNEIFQYVSTTTKVPPPDMTSFPYGTRRSPAKIWPTTKRPAITPANNPKHSKDPHKTGPEDTTVLIETKRDYSTELSVTIAVGASLLFLNILAFAALYYKKDKRRHETHRRPSPQRNTTNDIAHIQNEEIMSLQMKQLEHDHECESLQAHDTLRLTCPPDYTLTLRRSPDDIPLMTPNTITMIPNTLTGMQPLHTFNTFSAGQNSTNLPHGHSTTRV

>Otolemur_garnettii_XP_003793086.1

MSSPRGLLWLPLFFTPFCVMLNCNVLLWITALAIKFTLIDSQAQYPVVNTNYGKIRGLRTPLPNEILGPVEQYLGVPYASPPTGERRFQPPEPPSSWTGVRNATQFAAVCPQHLDERSLLHDMLPVWFTANLDTLMTYVQDQNEDCLYLNIYVPTEDGANVRKNGDDITSNDRAEDEDIHEQNSKKPVMVYIHGGSYMEGTGNMIDGSILASYGNVIVITINYRLGILGFLSTGDQAAKGNYGLLDQIQALRWIEENVGAFGGDPKRVTIFGSGAGASCVSLLTLSHYSEGLFQKAIIQSGTALSSWAVNYQPAKYTRILADKVGCNMLDTTDMVECLRNKNYKELIQQTITPATYHIAFGPVIDGDVIPDDPQILMEQGEFLNYDIMLGVNQGEGLKFVDGIVDNEDGVTPNDFDFSVSNFVDNLYGYPEGKDTLRETIKFMYTDWADKENPETRRKTLVALFTDHQWVAPAVATADLHAQYGSPTYFYAFYHHCQSEMKPSWADSAHGDEVPYVFGIPMIGPTELFSCNFSKNDVMLSAVVMTYWTNFAKTGDPNQPVPQDTKFIHTKPNRFEEVAWSKYNPKDQLYLHIGLKPRVRDHYRATKVAFWLELVPHLHNLNEIFQYVSTTTKVPPPDMTSFPYGTRRSPTKIWPTTKRPAITPANNPKHAKDPHKTGGPEDTTVLIETKRDYSTELSVTIAVGASLLFLNILAFAALYYKKDKRRHETHRRPSPQRNTTNDIAHIQNEEILSLQMKQLEHDHECESLQAHDTLRLTCPPDYTLTLRRSPDDIPLMTPNTITMIPNTLTGMQPLHTFNTFSAGQNTTNLPHGHSTTRV

>Macaca_fascicularis_XP_015298939.1

MSRPQGLLWLPLLFTPVCVMLNSNVLLWITALAIKFTLIDSQAQYPVVNTNYGKIRGLKTPLPSEILGPVEQYLGVPYASPPTGERRFQPPEPPSSWTGIRNTTQFAAVCPQHLDERSLLHDMLPIWFTANLDTLMTYVQDQNEDCLYLNIYVPTEDGANTKKNADDITSNDRGEDEDIHDQNSKKPVMVYIHGGSYMEGTGNMIDGSILASYGNVIVITINYRLGILGFLSTGDQAAKGNYGLLDQIQALRWIEENVGAFGGDPKRVTIFGSGAGASCVSLLTLSHYSEGLFQKAIIQSGTALSSWAVNYQPAKYTRILADKVGCNMLDTTDMVECLRNKNYKELIQQTITPATYHIAFGPVIDGDVIPDDPQILMEQGEFLNYDIMLGVNQGEGLKFVDGIVDNEDGVTPNDFDFSVSNFVDNLYGYPEGKDTLRETIKFMYTDWADKENPETRRKTLVALFTDHQWVAPAVATADLHAQYGSPTYFYAFYHHCQSEMKPSWADSAHGDEVPYVFGIPMVGPTELFSCNFSKNDVMLSAVVMTYWTNFAKTGDPNQPVPQDTKFIHTKPNRFEEVAWSKYNPKDQLYLHIGLKPRVRDHYRATKVAFWLELVPHLHNLNEIFQYVSTTTKVPPPDMTSFPYGTRRSPAKIWPTTKRPAITPANNPKHSKDPHKTGPEDTTVLIETKRDYSTELSVTIAVGASLLFLNILAFAALYYKKDKRRHETHRRPSPQRNTTNDIAHIQNEEIMSLQMKQLEHDHECESLQAHDTLRLTCPPDYTLTLRRSPDDIPLMTPNTITMIPNTLTGMQPLHTFNTFSGGQNSTNLPHGHSTTRV

>Gorilla_gorilla_XP_004063796.1_plus_insert_A2

MSRPQGLLWLPLLFTPVCVMLNSNVLLWLTALAIKFTLIDSQAQYPVVNTNYGKIRGLRTPLPNEILGPVEQYLGVPYASPPTGERRFQPPEPPSSWTGIRNTTQFAAVCPQHLDERSLLHDMLPIWFTANLDTLMTYVQDQNEDCLYLNIYVPTEDGANTKKNADDITSNDRGEDKDIHDQNSKKPVMVYIHGGSYMEGTGNMIDGSILASYGNVIVITINYRLGILGFLSTGDQAAKGNYGLLDQIQALRWIEENVGAFGGDPKRVTIFGSGAGASCVSLLTLSHYSEGLFQKAIIQSGTALSSWAVNYQPAKYTRILADKVGCNMLDTTDMVECLRNKNYKELIQQTITPATYHIAFGPVIDGDVIPDDPQILMEQGEFLNYDIMLGVNQGEGLKFVDGIVDNEDGVTPNDFDFSVSNFVDNLYGYPEGKDTLRETIKFMYTDWADKENPETRRKTLVALFTDHQWVAPAVATADLHAQYGSPTYFYAFYHHCQSEMKPSWADSAHGDEVPYVFGIPMIGPTELFSCNFSKNDVMLSAVVMTYWTNFAKTGDPNQPVPQDTKFIHTKPNRFEEVAWSKYNPKDQLYLHIGLKPRVRDHYRATKVAFWLELVPHLHNLNEIFQYVSTTTKVPPPDMTSFPYGTRRSPAKIWPTTKRPAITPANNPKHSKDPHKTGPEDTTVLIETKRDYSTELSVTIAVGASLLFLNILAFAALYYKKDKRRHETHRRPSPQRNTTNDIAHIQNEEIMSLQMKQLEHDHECESLQAHDTLRLTCPPDYTLTLRRSPDDIPLMTPNTITMIPNTLTGMQPLHTFNTFSGGQNSTNLPHGHSTTRV

>Microcebus_murinus_XP_020140668.1

MSSPRGLLWLPLFFTPVCIMLNSNVLLWITALAIKFTLIDSHAQYPVVNTNYGKIRGLRTPLPNEILGPVEQYLGVPYASPPTGERRFQPPEPPSSWTGLRNATQFAAVCPQHLDERSLLHDMLPIWFTANLDTLMTYVQDQNEDCLYLNIYVPTEDGGNTKKNADDITSNDRGEDEDIHDQNSKKPVMVYIHGGSYMEGTGNMIDGSILASYGNVIVITINYRLGILGFLSTGDQAAKGNYGLLDQIQALRWIEENVGAFGGDPKRVTIFGSGAGASCVSLLTLSHYSEGLFQKAIIQSGTALSSWAVNYQPAKYTRILADKVGCNMLDTTDMVECLRNKNYKELIQQAITPATYHIAFGPVIDGDVIPDDPQILMEQGEFLNYDIMLGVNQGEGLKFVDGIVDNEDGVTPNDFDFSVSNFVDNLYGYPEGKDTLRETIKFMYTDWADKENPETRRKTLVALFTDHQWVAPAVATADLHAQYGSPTYFYAFYHHCQSEMKPSWADSAHGDEVPYVFGIPMIGPTELFSCNFSKNDVMLSAVVMTYWTNFAKTGDPNQPVPQDTKFIHTKPNRFEEVAWSKYNPKDQLYLHIGLKPRVRDHYRATKVAFWLELVPHLHNLNEIFQYVSTTTKVPPPDMTSFPYGTRRSPAKIWPTTKRPAITPANNPKHSKDPHKTGPEDTTVLIETKRDYSTELSVTIAVGASLLFLNILAFAALYYKKDKRRHETHRRPSPQRNTTNDIAHIQNEEIMSLQMKQLEHDHECESLQAHDTLRLTCPPDYTLTLRRSPDDIPLMTPNTITMIPNTLTGMQPLHTFNTFSGGQNSTNLPHGHSTTRV

>Macaca_mulatta_XP_014982385.1_plus_insert_A2

MSRPQGLLWLPLLFTPVCVMLNSNVLLWITALAIKFTLIDSQAQYPVVNTNYGKIRGLKTPLPSEILGPVEQYLGVPYASPPTGERRFQPPEPPSSWTGIRNTTQFAAVCPQHLDERSLLHDMLPIWFTANLDTLMTYVQDQNEDCLYLNIYVPTEDGANTKKNADDITSNDRGEDEDIHDQNSKKPVMVYIHGGSYMEGTGNMIDGSILASYGNVIVITINYRLGILGFLSTGDQAAKGNYGLLDQIQALRWIEENVGAFGGDPKRVTIFGSGAGASCVSLLTLSHYSEGLFQKAIIQSGTALSSWAVNYQPAKYTRILADKVGCNMLDTTDMVECLRNKNYKELIQQTITPATYHIAFGPVIDGDVIPDDPQILMEQGEFLNYDIMLGVNQGEGLKFVDGIVDNEDGVTPNDFDFSVSNFVDNLYGYPEGKDTLRETIKFMYTDWADKENPETRRKTLVALFTDHQWVAPAVATADLHAQYGSPTYFYAFYHHCQSEMKPSWADSAHGDEVPYVFGIPMVGPTELFSCNFSKNDVMLSAVVMTYWTNFAKTGDPNQPVPQDTKFIHTKPNRFEEVAWSKYNPKDQLYLHIGLKPRVRDHYRATKVAFWLELVPHLHNLNEIFQYVSTTTKVPPPDMTSFPYGTRRSPAKIWPTTKRPAITPANNPKHSKDPHKTGPEDTTVLIETKRDYSTELSVTIAVGASLLFLNILAFAALYYKKDKRRHETHRRPSPQRNTTNDIAHIQNEEIMSLQMKQLEHDHECESLQAHDTLRLTCPPDYTLTLRRSPDDIPLMTPNTITMIPNTLTGMQPLHTFNTFSGGQNSTNLPHGHSTTRV

>Homo_sapiens_XP_016885179.1

MSRPQGLLWLPLLFTPVCVMLNSNVLLWLTALAIKFTLIDSQAQYPVVNTNYGKIRGLRTPLPNEILGPVEQYLGVPYASPPTGERRFQPPEPPSSWTGIRNTTQFAAVCPQHLDERSLLHDMLPIWFTANLDTLMTYVQDQNEDCLYLNIYVPTEDGANTKKNADDITSNDRGEDEDIHDQNSKKPVMVYIHGGSYMEGTGNMIDGSILASYGNVIVITINYRLGILGFLSTGDQAAKGNYGLLDQIQALRWIEENVGAFGGDPKRVTIFGSGAGASCVSLLTLSHYSEGLFQKAIIQSGTALSSWAVNYQPAKYTRILADKVGCNMLDTTDMVECLRNKNYKELIQQTITPATYHIAFGPVIDGDVIPDDPQILMEQGEFLNYDIMLGVNQGEGLKFVDGIVDNEDGVTPNDFDFSVSNFVDNLYGYPEGKDTLRETIKFMYTDWADKENPETRRKTLVALFTDHQWVAPAVATADLHAQYGSPTYFYAFYHHCQSEMKPSWADSAHGDEVPYVFGIPMIGPTELFSCNFSKNDVMLSAVVMTYWTNFAKTGDPNQPVPQDTKFIHTKPNRFEEVAWSKYNPKDQLYLHIGLKPRVRDHYRATKVAFWLELVPHLHNLNEIFQYVSTTTKVPPPDMTSFPYGTRRSPAKIWPTTKRPAITPANNPKHSKDPHKTGPEDTTVLIETKRDYSTELSVTIAVGASLLFLNILAFAALYYKKDKRRHETHRRPSPQRNTTNDIAHIQNEEIMSLQMKQLEHDHECESLQAHDTLRLTCPPDYTLTLRRSPDDIPLMTPNTITMIPNTLTGMQPLHTFNTFSGGQNSTNLPHGHSTTRV

>Rhinopithecus_roxellana_chr7

MSRPQGLLWLPLLFTPVCVMLNSNVLLWITALAIKFTLIDSQAQYPVVNTNYGKIRGLKTPLPSEILGPVEQYLGVPYASPPTGERRFQPPEPPSSWTGIRNTTQFAAVCPQHLDERSLLHDMLPIWFTANLDTLMTYVQDQNEDCLYLNIYVPTEDGANTKKNADDITSNDRGEDEDIHDQNSKKPVMVYIHGGSYMEGTGNMIDGSILASYGNVIVITINYRLGILGFLSTGDQAAKGNYGLLDQIQALRWIEENVGAFGGDPKRVTIFGSGAGASCVSLLTLSHYSEGLFQKAIIQSGTALSSWAVNYQPAKYTRILADKVGCNMLDTTDMVECLRNKNYKELIQQTITPATYHIAFGPVIDGDVIPDDPQILMEQGEFLNYDIMLGVNQGEGLKFVDGIVDNEDGVTPNDFDFSVSNFVDNLYGYPEGKDTLRETIKFMYTDWADKENPETRRKTLVALFTDHQWVAPAVATADLHAQYGSPTYFYAFYHHCQSEMKPSWADSAHGDEVPYVFGIPMVGPTELFSCNFSKNDVMLSAVVMTYWTNFAKTGDPNQPVPQDTKFIHTKPNRFEEVAWSKYNPKDQLYLHIGLKPRVRDHYRATKVAFWLELVPHLHNLNEIFQYVSTTTKVPPPDMTSFPYGTRRSPAKIWPTTKRPAITPANNPKHSKDPHKTGPEDTTVLIETKRDYSTELSVTIAVGASLLFLNILAFAALYYKKDKRRHETHRRSSPQRNTTNDIAHIQNEEIMSLQMKQLEHDHECESLQAHDTLRLTCPPDYTLTLRRSPDDIPLMTPNTITMIPNTLTGMQPLHTFNTFSGGQNSTNLPHGHSTTRV

>Aotus_nancymaae_XP_012289844.1_plus_insert_A2

MSRPQGLLWLPLLFTPVCVMLNSNVLLWITALAIKFTLIDSQAQYPVVNTNYGKVRGLRTPLPNEILGPVEQYLGVPYASPPTGERRFQPPEPPSSWTGIRNATQFAAVCPQHLDERSLLHDMLPIWFTANLDTLMTYVQDQNEDCLYLNIYVPTEDGANTKKNADDITSNDRGEDEDIHDQNSKKPVMVYIHGGSYMEGTGNMIDGSILASYGNVIVITINYRLGILGFLSTGDQAAKGNYGLLDQIQALRWIEENVGAFGGDPKRVTIFGSGAGASCVSLLTLSHYSEGLFQKAIIQSGTALSSWAVNYQPAKYTRILADKVGCNMLDTTDMVECLRNKNYKELIQQTITPATYHIAFGPVIDGDVIPDDPQILMEQGEFLNYDIMLGVNQGEGLKFVDGIVDNEDGVTPNDFDFSVSNFVDNLYGYPEGKDTLRETIKFMYTDWADKENPETRRKTLVALFTDHQWVAPAVATADLHAQYGSPTYFYAFYHHCQSEMKPSWADSAHGDEVPYVFGVPMIGPTELFSCNFSKNDVMLSAVVMTYWTNFAKTGDPNQPVPQDTKFIHTKPNRFEEVAWSKYNPKDQLYLHIGLKPRVRDHYRATKVAFWLELVPHLHNLNEIFQYVSTTTKVPPPDMTSFPYGTRRSPAKIWPTTKRPAITPANNPKHSKDPHKTGPEDTTVLIETKRDYSTELSVTIAVGASLLFLNILAFAALYYKKDKRRHETHRRPSPQRNTTNDIAHIQNEEMMSLQMKQLEHDHECESLQAHDTLRLTCPPDYTLTLRRSPDDIPLMTPNTITMIPNTLTGMQPLHTFNTFSGGQNSTNLPHGHSTTRV

>Pan_troglodytes_XP_016798712.1_plus_insert_A2

MSRPQGLLWLPLLFTPVCVMLNSNVLLWLTALAIKFTLIDSQAQYPVVNTNYGKIRGLRTPLPNEILGPVEQYLGVPYASPPTGERRFQPPEPPSSWTGIRNTTQFAAVCPQHLDERSLLHDMLPIWFTANLDTLMTYVQDQNEDCLYLNIYVPTEDGANTKKNADDITSNDRGEDEDIHDQNSKKPVMVYIHGGSYMEGTGNMIDGSILASYGNVIVITINYRLGILGFLSTGDQAAKGNYGLLDQIQALRWIEENVGAFGGDPKRVTIFGSGAGASCVSLLTLSHYSEGLFQKAIIQSGTALSSWAVNYQPAKYTRILADKVGCNMLDTTDMVECLRNKNYKELIQQTITPATYHIAFGPVIDGDVIPDDPQILMEQGEFLNYDIMLGVNQGEGLKFVDGIVDNEDGVTPNDFDFSVSNFVDNLYGYPEGKDTLRETIKFMYTDWADKENPETRRKTLVALFTDHQWVAPAVATADLHAQYGSPTYFYAFYHHCQSEMKPSWADSAHGDEVPYVFGIPMIGPTELFSCNFSKNDVMLSAVVMTYWTNFAKTGDPNQPVPQDTKFIHTKPNRFEEVAWSKYNPKDQLYLHIGLKPRVRDHYRATKVAFWLELVPHLHNLNEIFQYVSTTTKVPPPDMTSFPYGTRRSPAKIWPTTKRPAITPANNPKHSKDPHKTGPEDTTVLIETKRDYSTELSVTIAVGASLLFLNILAFAALYYKKDKRRHETHRRPSPQRNTTNDIAHIQNEEIMSLQMKQLEHDHECESLQAHDTLRLTCPPDYTLTLRRSPDDIPLMTPNTITMIPNTLTGMQPLHTFNTFSGGQNSTNLPHGHSTTRV

>Y_Macaca_mulatta_XP_014984076.1

MSRPRGLLWLPLFFTSVCVMLNSNVIFWITALAIKFTLIDSQAQYPVVNTNYGKIRGLKTPLPSEILGPVEQYLGVPYASPPTGERRFQPPESPSSWTGIRNATQFAAVCPQHLDERFLLHDMLPIWFTLNLDTLMTYVQDQNEDCLYLNIYVPTEDGTIIKRNDDDITSNDRGEDKDIHEQNSKKPVMVYIHGGSYMEGTGNMIDGSILASYGNVIVITINYRLGILGFLSTGDQAAKGNYGLLDQIQALRWIEENVGAFGGDPKRVTIFGSGAGASCVSLLTLSHYSEGLFQKAIIQSGTALSSWAVNYQPAKYTRILADKVGCNMLDTTDVVECLRNKNYKELIQQTITPATYHIAFGPVIDGDVIPDDPQILMEQGEFLNYDIMLGVNQGEGLKFVDGIVDNEDGVTPNDFDFSVSNFVDNLYGYPEGKDTLRETIKFMYTDWADKENPETRRKTLVALFTDHQWVAPAVATADLHAQYGSPTYFYAFYHHCQSEMKPSWADSAHGDEVPYVFGIPMVGPTELFSCNFSKNDVMLSAVVMTYWTNFAKTGDPNQPVPQDTKFIHTKPNRFEEVAWSKYNPKDQLYLHIGLKPRVRDHYRATKVAFWLELVPHLHNLNEIFQYVSTTTKVPPPDMTSFPYGTRRSPAKIWPTTKRPAITPANNPKHSKDPHKTGPEDTTVLIETKRDYSTELSVTIAVGASLLFLNILAFAALYYKKDKRRHETHRRPSPQRNTTNDIAHIQNEEIMSLQMKQLEHDHECESLQAHDTLRLTCPPDYTLTLRRSPDDIPLMTPNTITMIPNTLTGMQPLHTFNTFSGGQNSTNLPHGHSTTRV

>Y_Pan_troglodytes_XP_009444042.1

MLRPQGLLWLPLLFTSVCVMLNSNVLLWITALAIKFTLIDSQAQYPVVNTNYGKIQGLRTPLPSEILGPVEQYLGVPYASPPTGERRFQPPESPSSWTGIRNATQFAAVCPQHLDERFLLHDMLPIWFTANLDTLMTYVQDQNEDCLYLNIYVPTEDGTNIKRNADDITSNDHGEDEDIHEQNSKKPVMVYIHGGSYMEGTGNMIDGSILASYGNVIVITINYRLGILGFLSTGDQAAKGNYGLLDQIQALRWIEENVGAFGGDPKRVTIFGSGAGASCVSLLTLSHYSEGLFQKAIIQSGTALSSWAVNYQPAKYTRILADKVGCNMLDTTDMVECLKNKNYKELIQQTITPATYHIAFGPVIDGDVIPDDPQILMEQGEFLNYDIMLGVNQGEGLKFVDGIVDNEDGVTPNDFDFSVSNFVDNLYGYPEGKDILRETIKFMYTDWADKENPETRRKTLVALFTDHQWGAPAVATADLHAQYGSPTYFYAFYHHCQSEMKPSWADSAHGDEVPYVFGIPMIGPTELFRCNFSKNDVMLSAVVMTYWTNFAKTGDPNQPVPQDTKFIHTKPNRFQEVAWSKYNPKDQLYLHIGLKPRVRDHYRATKVAFWLELVPHLHNLNEIFQYVSTTTKVPPPDMTSFPYGTRRSPAKIWPTTKRPAITPANNPKHSKDPHKTGPEDTTVLIETKRDYSTDLSVTIAVGASLLFLNILAFAALYYKKDKRRHETHRRPSPQRNTTNDIPHIQNEEIMSLQMKQLEHDHECESLQAHDALRLTCPPDYTLTLRRSPDDIPLMTPNTITMIPNTLTGMQPLHTFNTFSGGQNSTNLPHGHSTTRV

>Y_Homo_sapiens_XP_011529732.1

MLRPQGLLWLPLLFTSVCVMLNSNVLLWITALAIKFTLIDSQAQYPVVNTNYGKIQGLRTPLPSEILGPVEQYLGVPYASPPTGERRFQPPESPSSWTGIRNATQFSAVCPQHLDERFLLHDMLPIWFTTSLDTLMTYVQDQNEDCLYLNIYVPMEDGTNIKRNADDITSNDHGEDKDIHEQNSKKPVMVYIHGGSYMEGTGNMIDGSILASYGNVIVITINYRLGILGFLSTGDQAAKGNYGLLDQIQALRWIEENVGAFGGDPKRVTIFGSGAGASCVSLLTLSHYSEGLFQKAIIQSGTALSSWAVNYQPAKYTRILADKVGCNMLDTTDMVECLKNKNYKELIQQTITPATYHIAFGPVIDGDVIPDDPQILMEQGEFLNYDIMLGVNQGEGLKFVDGIVDNEDGVTPNDFDFSVSNFVDNLYGYPEGKDTLRETIKFMYTDWADKENPETRRKTLVALFTDHQWVAPAVATADLHAQYGSPTYFYAFYHHCQSEMKPSWADSAHGDEVPYVFGIPMIGPTELFSCNFSKNDVMLSAVVMTYWTNFAKTGDPNQPVPQDTKFIHTKPNRFEEVAWSKYNPKDQLYLHIGLKPRVRDHYRATKVAFWLELVPHLHNLNEIFQYVSTTTKVPPPDMTSFPYGTRRSPAKIWPTTKRPAITPANNPKHSKDPHKTGPEDTTVLIETKRDYSTELSVTIAVGASLLFLNILAFAALYYKKDKRRHETHRHPSPQRNTTNDITHIQNEEIMSLQMKQLEHDHECESLQAHDTLRLTCPPDYTLTLRRSPDDIPFMTPNTITMIPNTLMGMQPLHTFKTFSGGQNSTNLPHGHSTTRV

>Cavia_porcellus_XP_023417699.1_plus_insert_A2

MWKRTMSRPKGLLWLPLFCTPVCIMVNSNVLLWITVLAIKFTVIDSQAQYPVVNTNYGKIRGLRTPLPNEILGPVEQYLGVPYASPPTGERRFQPPEPPSSWTGVRNATQFAAVCPQHLDERSLLHDMLPIWFTANLDTLMTYVQDQNEDCLYLNIYVPTEDGANRKRIADDITSNDRGDDEDIHDQNSKKPVMVYIHGGSYMEGTGNMIDGSILASYGNVIVITINYRLGILGFLSTGDQAAKGNYGLLDQIQALRWIEENVAAFGGDPKRVTIFGSGAGASCVSLLTLSHYSEGLFQKAIIQSGTALSSWAVNYQPAKYTRMLADKVGCNMLDTTDMVECLRNKNHKELIQQTITPATYHIAFGPVIDGDVIPDDPQILMEQGEFLNYDIMLGVNQGEGLKFVDGIVDNEDGVTPNDFDFSVSNFVDNLYGYPEGKDTLRETIKFMYTDWADKENPETRRKTLVALFTDHQWVAPAVATADLHAQYGSPTYFYAFYHHCQSEMKPTWADSAHGDEVPYVFGIPMIGPTELFSCNFSKNDVMLSAVVMTYWTNFAKTGDPNQPVPQDTKFIHTKPNRFEEVAWSKYNPKDQLYLHIGLKPRVRDHYRATKVAFWLELVPHLHNLNEIFQYVSTTTKVPPPDMTSFPYGTRRSPAKIWPTTKRPAITPANNPKHSKDLHKTGPEDTTVLIETKRDYSTELSVTIAVGASLLFLNILAFAALYYKKDKRRHETHRRPSPQRNTTNDIAHIQNEEIMSLQMKQLEHDHECESLQAHDTLRLTCPPDYTLTLRRSPDDIPLMTPNTITMIPNTLTGMQPLHTFNTFSGGQNSSNLPHGHSTTRV

>Chinchilla_lanigera_XP_013361076.1

MWRRTMSRPKGLLWLPLFCTPVCVMVNSNVLLWITALAVKFTVIDSQAYYPVVNTNYGKIRGLRTPLPNEILGPVEQYLGVPYASPPTGERRFQPPEPPSSWTGIRNATQFAAVCPQHLDERSLLHDMLPIWFTANLDTLMTYVQDQNEDCLYLNIYVPTEDGANTKKIADDITSNDRGDDEDIHDQNGKKPVMVYIHGGSYMEGTGNMIDGSILASYGNVIVITINYRLGILGFLSTGDQAAKGNYGLLDQIQALRWIEENVAAFGGDPKRVTIFGSGAGASCVSLLTLSHYSEGLFQKAIIQSGTALSSWAVNYQPAKYTRMLADKVGCNMLDTTDMVECLRNKNYKELIQQAITPATYHIAFGPVIDGDVIPDDPQILMEQGEFLNYDIMLGVNQGEGLKFVDGIVDNEDGVTSNDFDFSVSNFVDNLYGYPEGKDTLRETIKFMYTDWADKENPETRRKTLVALFTDHQWVAPAVATADLHAQYGSPTYFYAFYHHCQSEMKPSWADSAHGDEVPYVFGIPMIGPTELFSCNFSKNDVMLSAVVMTYWTNFAKTGDPNQPVPQDTKFIHTKPNRFEEVAWSKYNPKDQLYLHIGLKPRVRDHYRATKVAFWLELVPHLHNLNEIFQYVSTTTKVPPPDMTSFPYGTRRSPAKIWPTTKRPAITPANNPKHSKDPHKTAPEDTTVLIETKRDYSTELSVTIAVGASLLFLNILAFAALYYKKDKRRHETHRRPSPQRNTTNDIAHIQNEEIMSLQMKQLEHEHECESLQAHDTLRLTCPPDYTLTLRRSPDDIPLMTPNTITMIPNTLTGMQPLHTFNTFSGGQNSTNLPHGHSTTRV

>Fukomys_damarensis_XP_010602072.1_plus_insert_A2

MWKKTMSRPRGLLWLPLFCTPVCVMVNSNVLLWVTALAVKFTLIDSQAQYPVVNTNYGKIRGLRTPLPNEILGPVEQYLGVPYASPPTGERRFQPPEPPSSWTGVRNATQFAAVCPQHLDERSLLHDMLPIWFTANLDTLMTYVQDQNEDCLYLNIYVPTEDGANTKKVADDITSNDRGDDEDIHDQNTKKPVMVYVHGGSYMEGTGNMIDGSILASYGNVIVITVNYRLGILGFLSTGDQAAKGNYGLLDQIQALRWIEENVGAFGGDPKRVTIFGSGAGASCVSLLTLSHYSEGLFQKAIIQSGTALSSWAVNYQPAKYTRMLADKVGCNMLDTTDMVECLRNKNYKELIQQAITPATYHIAFGPVIDGDVIPDDPQILMEQGEFLNYDIMLGVNQGEGLKFVDGMVDSEDGVTPNDFDFSVSNFVDNLYGYPEGKDTLRETIKFMYTDWADKENPETRRKTLVALFTDHQWVAPAVATADLHAQYGSPTYFYAFYHHCQSEMKPSWADSAHGDEVPYVFGIPMIGPTELFSCNFSKNDVMLSAVVMTYWTNFAKTGDPNQPVPQDTKFIHTKPNRFEEVAWSKYNPKDQLYLHIGLKPRVRDHYRATKVAFWLELVPHLHNLNEIFQYVSTTTKVPPPDMTSFPYGTRRSPAKIWPTTKRPAITPANSPKQAKDAQKPGPEDTTVLIETKRDYSTELSVTIAVGASLLFLNILAFAALYYKKDKRRHETHRRPSPQRNTTNDIAHIQNEEILSLQMKQLEHDHECESLQAHDTLRLTCPPDYTLTLRRSPDDIPLMTPNTITMIPNALPAMQPLHTFNTFSGGQNSTNLPHGHSTTRV

>Heterocephalus_glaber_XP_004867397.1

MWKRTMSRPRGLLWLPLFCTPVCVMVSSKVLLWVTALSVKFTLTDSQAQYPVVNTNYGKIRGLRTPLPNEILGPVEQYLGVPYASPPTGERRFQPPEPPSSWTGVRNATQFAGVCPQHLDERSLLHDMLPIWFTANLDTLMTYVQDQNEDCLYLNIYVPTEDGANTKKVADDITTNDRGDDEDIHDQNSKKPVMVYIHGGSYMEGTGNMIDGSILSSYGNVIVITINYRLGILGFLSTGDQAAKGNYGLLDQIQALRWIEENVGAFGGDPKRVTIFGSGAGASCVSLLTLSHYSEGLFQKAIIQSGTALSSWAVNYQPAKYTRMLADKVGCNMLDTTDMVECLRNKNYKELIQQTITPATYHIAFGPVIDGDVIPDDPQILMEQGEFLNYDIMLGVNQGEGLKFVDGMVDNEDGVTPNDFDFSVSNFVDNLYGYPEGKDTLRETIKFMYTDWADKENPETRRKTLVALFTDHQWVAPAVATADLHAQYGSPTYFYAFYHHCQSEMKPSWADSAHGDEVPYVFGIPMIGPTELFSCNFSKNDVMLSAVVMTYWTNFAKTGDPNQPVPQDTKFIHTKPNRFEEVAWSKYNPKDQLYLHIGLKPRVRDHYRATKVAFWLELVPHLHNLNEIFQYVSTTTKVPPPDMTSFPYGTRRSPAKIWPTTKRPAITPANNPKQAKEPHKPGPEDTTVLIETKRDYSTELSVTIAVGASLLFLNILAFAALYYKKDKRRHETHRRPSPQRNTTNDIAHIQNEEIMSLQMKQLEHDHECESLQAHDTLRLACPPDYTLTLRRSPDDIPLMTPNTITMIPNTLTGMQPLHTFNTFSGGQNSTNLPHGHSTTRV

>Ictidomys_tridecemlineatus_XP_005335587.3

MSRPKGLLWLPLFFTPVCVMLNSNVLLWITALAIKFTLIDSQAQYPVVNTNYGKIRGLRTPLPNEILGPVEQYLGVPYASPPTGERRFQPPEPPSSWTGVRNATQFAAVCPQHLDERSLLHDMLPIWFTANLDTLMTYVQDQNEDCLYLNIYVPTEDGANTKKIADDITSNDRGDDEDIHDQSSKKPVMVYIHGGSYMEGTGNMIDGSILASYGNVIVITINYRLGVLGFLSTGDQAAKGNYGLLDQIQALRWIEENVGAFGGDPKRVTIFGSGAGASCVSLLTLSHYSEGLFQKAIIQSGTALSSWAVNYQPAKYTRLLADKVGCNMLDTTDLVECLRHKSHKELIQQAITPATYHIAFGPVIDGDVIPDDPQILMEQGEFLNYDIMLGVNQGEGLKFVDGIVDNEDGVTPNDFDFSVSNFVDNLYGYPEGKDTLRETIKFMYTDWADKENPETRRKTLVALFTDHQWVAPAVATADLHAQYGSPTYFYAFYHHCQSEMKPSWADSAHGDEVPYVFGVPMIGPTELFSCNFSKNDVMLSAVVMTYWTNFAKTGDPNQPVPQDTKFIHTKPNRFEEVAWSKYNPKDQLYLHIGLKPRVRDHYRATKVAFWLELVPHLHNLNEIFQYVSTTTKVPPPDMTSFPYGTRRSPAKIWPTTKRPAITPASGPKHSKDSHKTGPEDTTVLIETKRDYSTELSVTIAVGASLLFLNILAFAALYYKKDKRRHETHRRPSPQPRSATNDMAHIQNEEILSLQMKQLDHDHECESLQAHDTLRLTCPPDYTLTLRRSPDDIPLMTPNTITMIPNTLTGMQPLHTFNTFSGGQNSTNLPHGHSTTRV

>Nannospalax_galilil_XP_008821156.1

MSRPKRRLWLPLLCTHVCVMLNSNVLLWITVLAIKFTLIDSQAQYPVVNTNYGKIRGLRTPLPNEILGPVEQYLGVPYASPPTGERRFQPPEPPSSWTGVRNATQFAPVCPQHLDERSLLHDMLPIWFTANLDTLMTYVQDQNEDCLYLNIYVPTEDGANTKKIADDITSNERGDDEDIHDQNSKKPVMVYIHGGSYMEGTGNMIDGSILASYGNVIVITINYRLGILGFLSTGDQASKGNYGLLDQIQALRWIEDNVGAFGGDPKRVTIFGSGAGASCVSLLTLSHYSEGLFQKAIIQSGTALSSWAVNYQPAKYTRILADKVGCNMLDTTDMVECLRNKNYKELIQQAITPATYHIAFGPVIDGDVIPDDPQILMEQGEFLNYDIMLGVNQGEGLKFVDGIVDSEDGVTPNDFDFSVSNFVDNLYGYPEGKDTLRETIKFMYTDWADKENPETRRKTLVALFTDHQWVAPAVATADLHAQYGSPTYFYAFYHHCQSEMKPSWADSAHGDEVPYVFGIPMIGPTELFSCNFSKNDVMLSAVVMTYWTNFAKTGDPNQPVPQDTKFIHTKPNRFEEVAWSKYNPKDQLYLHIGLKPRVRDHYRATKVAFWLELVPHLHNLNEIFQYVSTTTKVPPPDMTSFPYGTRRSPAKIWPTTKRPAITPANSPKHSKDNQKTGPEDTTVLIETKRDYSTELSVTIAVGASLLFLNILAFAALYYKKDKRRHETHRRPSPQRNTTNDIAHIQNEEIMSLQMKQLDHEHECESLQAHDTLRLTCPPDYTLTLRRSPDDIPLMTPNTITMIPNTLTGMHPLHTFNTFSGGQNSTNLPHGHSTTRV

>Octodon_degus_XP_004647418.1

MSRPKGLLWVPLFCTPVCVMVNSNVLLWITALAIKFTLIDSQAQYPVVNTNYGKIRGLRTPLPNEILGPVEQYLGVPYASPPTGERRFQPPEPPSSWTGVRNATQFAGVCPQHLDERSLLHDMLPIWFTANLDTLMTYVQDQNEDCLYLNIYVPTEDGANTKKVADDVTSNDRADDEDIHDQNSKKPVMVYIHGGSYMEGTGNMIDGSILASYGNVIVITINYRLGILGFLSTGDQAAKGNYGLLDQIQALRWVEENVAAFGGDPKRVTIFGSGAGASCVSLLTLSHYSEGLFQKAIIQSGTALSSWAVNYQPAKYTRMLADKVGCNMLDTTDLVECLRNKNYKELIQQAITPATYHIAFGPVIDGDVIPDDPQILMEQGEFLNYDIMLGVNQGEGLKFVDGIVDNEDGVTPNDFDFSVSNFVDNLYGYPEGKDTLRETIKFMYTDWADKENPETRRKTLVALFTDHQWVAPAVATADLHAQYGSPTYFYAFYHHCQSEMKPSWADSAHGDEVPYVFGIPMIGPTELFSCNFSKNDVMLSAVVMTYWTNFAKTGDPNQPVPQDTKFIHTKPNRFEEVAWSKYNPKDQLYLHIGLKPRVRDHYRATKVAFWLELVPHLHNLNEIFQYVSTTTKVPPPDMTSYPYGTRRSPAKIWPTTKRPAITPANNPKHSKDSQKTGPEDTTVLIETKRDYSTELSVTIAVGASLLFLNILAFAALYYKKDKRRHETHRRPSPQRSTTNDIAHMQNEEIMSLQMKQLDHDHECESLQAHDTLRLTCPPDYALTLRRSPDDIPLMTPNTITMIPSTLTGMQPLHTFNTFSGGQNSTNLPHGHSTTRV

>Mus_musculus_CAST/Ei_(Maxeiner et al., 2020)

MPAPAPALLCLALALASAQPSPPPPPFPVVATNYGKLRGVRAALPGDVLGPVTQFLGVPYAAPPTGERRFQPPEPPSSWAGVRDATRFAPVCPQHLDERALLRDRLPAWFAANLDAIAAYVQDQSEDCLYLNLYVPGGANGKKMADDVTGNDHGDDQDSRDPGVGGAAAAAARKPVMVYIHGGSYMEGTANIVDGSVLASYGDVIVVTVNYRLGVLGFLSTGDQAAKGNYGLLDQIQALRWVEENAGAFGGDPDRVTVFGSGAGASCVSLLTLSHYSEGLFQKAIIQSGTALSSWAVNYQPARYARALGERVGCATPDPGSPPGSPPGWDSASLVSCLRGKAAGELARARVTPATYHVAFGPTVDGDVIPDDPQILMEQGEFLNYDIMLGVNQGEGARFVDGLGGGHDGGYGGYGGGYGGGVEDDEVQDGGPDGAAGGVSAGEFDLAVSGFIDDLYGRPEGRGDALRETVKFMYTDWADRDSPEARRKTLVALFTDHQWVAPAVATADLHARYGSPTYFYAFYHRCHGGGGGGGGVDGVAGGVAGGVGGEEARPAWADAAHGDEVPYVFGVPMAGPGDVFGCNFSRNDVMLSAVVMTYWTNFAKTGDPNQPVAQDTRFVHTRPNRFEEVAWAKYDPRGQLYLHIGLRPRVRDHYRAAKVAFWLELVPHLHGLAADPGAYLSAAATRAAPSGDPDRDPGGGVGGRRRPRPATRRPAVMTSSSMASGSGMTSSSGSGMTSSSSSGMTSSSGSSASAVLIETRRDYSTELSVTIAVGASLLFLNVLAFAALYYKKDKRRHETHRRPPPPRPPQAPPSAAAADRNPRPDPGPASRRGGECGAVVTAMAAEASAGGLGHDGVGGVIGGVAGLRLACPPDYALTMRRSPDDVPRAGTGPGAMTLIPGALGGGGGGAVHGFNTFGSGVGIAGVAGVAGVTGVATSQAGPGLPHGHSTTRV

>Mus_musculus_C57BL/6J_(Maxeiner et al., 2020)

MPAPVPALLCLALALASAQPSPPPPPPFPVVATNYGKLRGVRAALPGDVLGPVTQFLGVPYAAPPTGERRFQPPEPPSSWAGVRDATRFAPVCPQHLDERALLRDCLPAWFAANLDAIAAYVQDQSEDCLYLNLYVPGGANGKKMADDVTGNDHGDDQDSRDPGVGGAAAAAARKPVMVYIHGGSYMEGTANIVDGSVLASYGDVIVVTVNYRLGVLGFLSTGDQAAKGNYGLLDQIQALRWVEENAGAFGGDPDRVTVFGSGAGASCVSLLTLSHYSEGLFQKAIIQSGTALSSWAVNYQPARYARALGERVGCATPDPGSPPGSPPGWDSASLVSCLRGKAAGELARARVTPATYHVAFGPTVDGDVIPDDPQILMEQGEFLNYDIMLGVNQGEGARFVDGLGGGHDGGYGGYGGGYGGGVEDDEVQDGGPDGAAGGVSAGEFDLAVSGFINDLYGRPEGRGDALRETVKFMYTDWADRDSPEARRKTLVALFTDHQWVAPAVATADLHARYGSPTYFYAFYHRCHGGGGGGGGVDGVAGGVAGGVGGEEARPAWADAAHGDEVPYVFGVHMAGPGDVFGCNFSRNDVMLSAVVMTYWTNFAKTGDPNQPVAQDTRFVHTRPNRFEEVAWAKYDPRGQLYLHIGLRPRVRDHYRAAKVAFWLELVPHLHGLAADPGAYLSAAATRAAPSGDPDRDPGGGGGGRRRPRPATRRPAVMTSSSMASGSGMTSSSGSGMTSSSGSSASAVLIETRRDYSTELSVTIAVGASLLFLNVLAFAALYYKKDKRRHETHRRPPPPRPPQAPPSAAAADRNPRPDPGPAGRRGGECGAVVTAMAAEASAGGLGHDGVGGVGVGGVIGGVAGLRLACPPDYALTLRRSPDDVPRAGAGPGTMTLIPGALGGGGGGAVHGFNTFGSGVGVAGVAGVATSQAGPGLPHGHSTTRV

>Peromyscus_maniculatus_(Maxeiner et al., 2020)

MSPPLWAAALVTAAAMLVPAAGSAASMAGSQHPVVATHYGRLRGTRVTLPGALLGPVLRFLGVPYAAAPTGPRRFQAPEPPASWPGVRNATGFAPVCPQPLDTRALPRDMLPVWFAAAAPEALGAALGEQSEDCLFLNLYVPAGAPGANARKIADDIASNDRGDDEEARDPGGRKPVMVYIHGGSYMEGTGNIMDGSVLASYGDVIVVTVNYRLGVLGFLSTGDQAAKGNYGLLDQIQALRWVEENVGAFGGDPKRVTVFGSGAGASCVSLLTLSHYSEGLFQKAIIQSGTALSSWAVNYQPARYARALGAQLGCPGNTRSSYMSSYMSDPGSSWDSAALVSCLRRAAWRELSRPRVTPAPYHVAFGPVIDGDVIPDDPQILMEQGEFLNYDVMLGVNQAEGLRFARAAGAPDTYDTYDNMRNTYDNIRDTYDRTYDKYDRENGDYRDDSMENDFRGHHGRRRDHEEDTEHGYQGRRVNTDDNMDDNMDDNMDSNTENDYWANRGSRGRRVDPDNMDDTDDTDTMENTDNMGNQYRDRRVDLDNTYDRTYDAYRDARGRRGRRVEPDDTDTMENTDNMGNQYRDRRGRRVDPDHTDNMDDTDNTYDDYRGNRGRRGRRVDPDNTDDTENDFRDRRADRTHDHTHDDTYDAADTYDADDGVSAAAFDAAIAAFVDRLYGDPGGVADPGSDPGSGRVSSSSALRETVKFMYTDWAAVSGSRAGSGVAGVTGGSAGSSSWRRRALTSLFTDHQWAAPAVATADLHARYGSPTYFYAFAHRCDPGSGDPGSDDPGSDPGSDPRARPAWAEGAAHGDELPYVFGVPLLLLGGAGVTGGDAGVAEGVTGVTGWAEIFACNFTRGDVMLSAGVMTYWTNFAKTGDPNRPVPQDTKFVHTRPNRFEEVAWAKYDPRAQLYLHIGLRPRVRDHYRATKVAFWLELVPHLHGLRGDLLLQYVSTTPTQAPPLPGSTPSTADAAHHPPGPFPKLFHATRRPISPGHAQPGGHDGARRPITPGHAQSGGHDGTKRGFRGPGGPGGGPGGTEDATVLIETKRDYSTELSVTIAVGASLLFLNVLAFAALYYKKDKRRHETHRRLHRGDRGHPRGHHGVTGVTDAATKPPVAAQGPPDYALTLRRSPDDLPPARGAPPSTITRVPDGLAGAGPGIGGGVSAVGVAGAGLQPQPPPPLHTFNAFGAAGVGGHSTTRV

>Microtus_ochrogaster_(Maxeiner et al., 2020)

MPAPRCAWALAACCCVFLACCRATPPPPAPVVSTRYGKLRGVRVPLPGGVLDPVDRFLGVPYAAAPTGERRFLPPEPPPPWPGVRDATRFAPVCPQPLDARALPRDMLPVWHASNPGPVAARVREQSEDCLFLNVYAPAGGNMGKTTDDDIMGHGRGHDEGARDPADPRDPGKPVMVYIHGGSYMDGSGNLVDGGVLASYGDVIVVTVNYRLGVLGFLSTGDQAARGNYGLLDQIQALRWVRENAGAFGGDPGRVTVFGSGAGASCVSLLTLSHYSEGLFQKAIIQSGTALSSWAVNYEPAKSARALAEQLGCGSEVAGSPEVAGSEVAGSPPDTSSSTAALVSCLRGAGWRDLSRARVRAPPYRVAFGPAVDGDVIADDPQVLMEQGEFLNHDVLLGVNQGEGLRFLDGVPGASGDDDADTDAEKNTYDSDDDVGSDAGASASRATTAGNEDEETEDEDAHRGNAGASASRAAFDRAVAAFVDRLYGGGGDPDGDLSDPGGDPDALRETVKFMYTDWASRASAASRRKALVALFTDHQWVAPAVATADLHARYGSATYFYAFGHRCAGGHVGGGHDGGGGHDDSNGGHVGGGHDTGGHDGSDGGGHDSGGHDTGGHDGGNSGGHDGGGHDGGGHDSGGHDGGGGHDDSNGGHDGGGHDDSNGGHDSGGHDSGGHDDSGGHDGADDPAHPARPAWAEAAHGDEVPYVFGVPLAAGAGASGADLFGCNFSRDDVMLSAAVMTYWTNFAKTGDPNRPTPQDTRFAHTRPNRFEAVAWSRYDPRGQLYLHIGPRPRVRDHYRAAKVAFWLELVPRLHGLRERARYAGAGARDATDATDATGASDGAGNRDGRARAAAAATRRPPAVTGAAASGRRGRGGHVGANMAAKMAANDAHGATVLIETKRGHAYAAELSVTLAVGAALLLLNVLAFAALYYKRDRRRRRTAANMAATTSGRGCECRGGGGKPPPLPPPPLLPQRRDGGHVGGGGGDAVDSAPARAAHPLDYSLMLRRAPNDIMAPSTITAAAASGAHGFNGFAGHTATRV

>Cricetulus_griseus_(Maxeiner et al., 2020)

MAPPRSPAPVALALALALACPCSRAQGDPVVSTRYGRLRGLRAPLPGELLGPVEQFLGVPYAAPPTGARRFQPPEPPSSWPGVRNATRFAPVCPQPLDERALLRDMLPVWFSANLDTLAAYVQDQAEDCLYLNVYVPTEDDIHDPSSRKPVMVYIHGGSYMEGTGNMIDGSVLASYGNVIVITVNYRLGILGFLSTGDQAAKGNYGLLDQIQALRWVEENAGAFGGDPKRVTIFGSGAGASCVSLLTLSHYSEGLFQKAIIQSGTALSSWAVNYQPAKYTRALADQVGCNMLDTADLVECLRRVGWRELSQQRVAPATYHVAFGPVIDGDVIPDDPQILMEQGEFLNYDIMLGVNQGEGLRFVDGFVDTGADDGGGGDADPADADGYGGGGGGGAGVTPSDFDLAVSSFVDNLYGYPEGKDALRETIKFMYTDWADRENAETRRKTLVALFTDHQWVAPAVATADLHAQYGSPTYFYAFYHHCQSDAKPPWADAAHGDEVPYVFGVPMLGPTDLFSCNFSKNDVMLSAVVMTYWTNFAKTGDPNRPVPQDTKFIHTKPNRFEEVAWSKYSPREQLYLHIGLKPRVRDHYRATKVAFWLELVPHLHSLHDLFQYVSTTTTRAPPPDTPSSAFPYGGGGRRAPGAAAKPWPATRRPPGRSSFPPPLPGSRPGSGSRPGSGGGSGARDAAAAGRAGAEDATVLIETKRDYSTELSVTIAVGASLLFLNILAFAALYYKKDKRRHETHRRHPHAHHRHHHLHHHHRGGAAGGAGGGDDARDDALAALRAKPPCPPPACSAPPLPAVPGVRDDDALPRPAPSGPPDYALTLRRSPDDVPPNAIAVIPDCGLTPSAAGAGLPALQPPPQLHTFNAFGAGQNGANLPHGHSTTRV

>Castor_canadensis_(Maxeiner et al., 2020)

MSRPKGLLWLPLFFTPVCVMLNSNVLLWITALAIKFTLIDSQAQYPVVNTNYGKIRGLRTPLPNEILGPVEQYLGVPYASPPTGERRFQPPEPPSSWTGVRNATQFAAVCPQHLDERSLLHDMLPIWFTANLDTLMTYVQDQNEDCLYLNIYVPTEDGANTKKIADDISSNDRGDDEDIHEQSSRKPVMVYIHGGSYMEGTGNMIDGSILASYGNVIVITVNYRLGILGFLSTGDQAAKGNYGLLDQIQALRWIEENVGAFGGDPKRVTIFGSGAGASCVSLLTLSHYSEGLFQKAIIQSGTALSSWAVNYQPAKYTRILADKVGCNMLDTTDMVECLRIKNYKELIQQAITPATYHIAFGPVIDGDVIPDDPQILMEQGEFLNYDIMLGVNQGEGLKFVDGIVDNEDGVTPNDFDFSVSNFVDNLYGYPEGKDTLRETIKFMYTDWADKENPETRRKTLVALFTDHQWVAPAVATADLHAQYGSPTYFYAFYHHCQSEMKPSWADSAHGDEVPYVFGIPMIGPTELFSCNFSKNDVMLSAVVMTYWTNFAKTGDPNQPVPQDTKFIHTKPNRFEEVAWSKYNPKDQLYLHIGLKPRVRDHYRATKVAFWLELVPHLHNLNEIFQYVSTTTKVPPPDMTSFPYGTRRSPAKIWPTTKRPAITPANNPKHSKDPHKTGPEDTTVLIETKRDYSTELSVTIAVGASLLFLNILAFAALYYKKDKRRHETHRRPSPQRNTTNDIGHIQNEEIMSLQMKQLEHDHECEPLQAHDTLRLTCPPDYTLTLRRSPDDIPLMTPNTITMIPNTLTGLQPLHTFNTFSGGQNSTNLPHGHSTTRV

>Meriones_unguiculatus_this_paper

MTSGALLVCLAVASLAACVSCSSLPPEDSGEGAGPVVVSTRYGRLRGMRVPLPGGSLGPVARFLGVPYAAPPTGPRRFQPPEPPAPWPGVRGAARFAPVCPQDADTRPDPAAMLPAWLAADPDAVAAHAREQDEDCLYLNLYVPAGVGGHVRSLTEDLSNDERGDDPDTRDPATRKPVMVFIHGDSYMAGTGNMMDGSVLASYGDVIVVTLNYRLGALGFLSTGDPAARGNYGLLDQMQALRWLRENAVAFGGDPARVTVFGSGAGASCVSLLTLSHYSEGLFQKAIIQSGTALSSWAVNYQPAAYARMLGARVGCGGDMTSATSPPDTMATPPLTSSVHDPPSPSAALVACLRRRGARELTRAAGSVPASAPFHVAFGPVIDGDVVPDDPQILMEQGEFLNYDILLGVNQAEGVALADPAHPDGGGDVTADGEEEEEVSAAGFELAVAAFVDALYGYPGGDVGVAGLGGGVAGWGSGAGGDSALRETARFMYTDWAEREGGAGSRRRALAAMMTDHQWAAPAVATADLHARYGSATYFYAFAHPCRGDAHPAWAAEAGAAHGDELPFVFGVPMLVLAAAGGGVGGVGSEGAAGSDVAVATAANAAALFPCNFTRNDVMLSAVVMTYWTNFAKTGDPNRPVPQDTKFAHTRPNRFEAVAWPKYTPRERLYLHVGLRPRVRDHYRATKVAFWLELVPHLHGLREAFPYLTTPTAAPRAQPGPRRAWPPTRRPAPPSSGRPASSSSSSASASSSRDSNPGPGEASVLIETRRDYSTELSVTIAVGASLLFLNILAFAALYYKKDKRRHETHHRRMAASGGTSGFASGLTSASGATSGSTSGFASGPTSGFASTSGPTSGYTAAHRPGNDALKRGREEDPGAAVTSPSSLDALRLPTGPPDYALTLRRAPDDAAPLAVPSAITMVPNALAGLPPLHAFGHSTTRV

>Y_Meriones_unguiculatus_this_paper

MTLGALLVCLAVASLAACVSCSSLPPEDSGEGAGPVVVSTRYGLLRGMRVPLPGGSLGPVARFLGVPYAAPPTGPRRFQPPEPPAPWPGVRGAARFAPVCPQDADTRPDPAAMLPAWLAADPDAVAAHAREQDEDCLYLNLYVPAGVGGHLRNLADDPSSDERGDDPDTRDPATRKPVMVFIHGDSYMAGTGNMMDGSVLASYGDVIVVTLNYRLGALGFLSTGDPAARGNYGLLDQMQALRWLRENAVAFGGDPARVTVFGSGAGASCVSLLTLSHYSEGLFQKAIIQSGTALSSWAVNYQPAVYARMLGARVGCGGDVMSATPLPDAAATPPRMSSSHDLPSASAALVACLRRRGARELTRAAGSVPASSPFHVAFGPVIDGDVVPDDPQILMEQGEFLNYDILLGVNQAEGVALADPAHPDSLGDIMADGEEEVSAAGFELAVAAFVDALYGYPGGDVGVAGWSGGAGGDSALRETARFMYTDWAEREGGAGSRRRALAAMMTDHQWAAPAVATADLHARYGSATYFYAFAHPCRGDAHPAWAAEAGAAHGDELPFVFGVPMLVLAAAGDGSVGGVGGEGATGTDVAAATAAALFPCNFTRNDVMLSAVVMTYWTNFAKTGDPNRPVPQDTKFAHTRPNRFEAVAWPKYTPRERLYLHVGLRPRVRDHYRATKVAFWLELVPHLHGLREAFPYLTTPTAAPHVPTGPRRVWPPTRRPALPSLGRPASSSSASSSSSQDSKVGPGEAVLIETRRDYSTELSVTIAVGASLLFLNILAFAALYYKKDRRRHETHHRRMAASGATSGFASASGTASGPTSDFVSASGHASGPASGFPSTSGPTSGFASTHCSGNDTGKRGREENPGAMVTSSVTSPSSLDAALRLPTGPPDYTLTLRRAPDDAPPLTAPSAITMMPNALAGLPQLHTFGHSTTRV

>Aplodontia_rufa_this_paper

MSRPRGLLWLPLFFTPVCVMLNSNVLLWITALAIKFTLIDSQAQYPVVNTNYGKIRGLRTPLPSEILGPVEQYLGVPYASPPTGERRFQPPEPPSSWTGIRNATQFAAVCPQHLDERSLLHDMLPIWFTANLDTLMTYVQDQNEDCLYLNIYVPTEDGANTKKIADDITSNERGDDEDIHDQSSKKPVMVYIHGGSYMEGTGNMIDGSILASYGNVIVITINYRLGILGFLSTGDQAAKGNYGLLDQIQALRWIEENVGAFGGDPKRVTIFGSGAGASCVSLLTLSHYSEGLFQKAIIQSGTALSSWAVNYQPAKYTRILADKVGCNMLDTTDMVECLRSKNHKELIQQAITPATYHIAFGPVIDGDVIPDDPQILMEQGEFLNYDIMLGVNQGEGLKFVDGIVDNEDGVTPNDFDFSVSNFVDNLYGYPEGKDTLRETIKFMYTDWADKENPETRRKTLVALFTDHQWVAPAVATADLHAQYGSPTYFYAFYHHCQSEMKPSWADSAHGDEVPYVFGIPMIGPTELFSCNFSKNDVMLSAVVMTYWTNFAKTGDPNQPVPQDTKFIHTKPNRFEEVAWSKYNPKDQLYLHIGLKPRVRDHYRATKVAFWLELVPHLHNLNEIFQYVSTTTKVPPPDMTSFPYGTRRSPAKIWPTTKRPAITPANNPKHNKDSHKTGPEDTTVLIETKRDYSTELSVTIAVGASLLFLNILAFAALYYKKDKRRHETHRRPSPQRNTTNDIAHIQNEEIMSLQMKQLEHDHECESLQAHDTLRLTCPPDYTLTLRRSPDDIPLMTPNTITMIPNTLTGMQPLHTFNTFSGGQNSTNLPHGHSTTRV

>Arvicola_amphibius_this_paper

MAPWPLACSVWALACCVLLVCPTWASPPAPVVSTRYGKLRGLRVPLPGGVLNPVDQFLGVPYAAPPTGERRFQPPDPPSAWPGVRDATRFAPVCPQPLDERALPRDMLPVWFTADALAAHVREQSEDCLFLNVYTPAGANVGKIAGDITGRERGDDEDARDPGDPGRKPVMVYIHGGSYMEGTGNLIDGSVLASYGDVIVVTVNYRLGVLGFLSTGDQAAKGNYGLLDQIQALRWVEENAGAFGGDPERVTVFGSGAGASCVSLLTLSHYSEGLFQKAIIQSGTALSSWAVNYQPAKYTRALAEQVGCGPASGDTASGDTAALVSCLRGMGWRELSRPRVSPAAYHVAFGPVVDGDVIPDDPQILMEQGEFLNYDVMLGVNQGEGLRFVDGIVAGDDADDAGDAYDGDAEDTRVEAENTRVGSKNARIGADGADNAYDDGNARVGRAGSTRVDAGNTYDEDEVARAGNRNARADADVARVDPESTRVGGGVSAADFDQAVSGFVDRLYGAPEGGDALRETIKFMYTDWADRESAASRRKALVALFTDHQWVAPAVATADLHARYGSPTYFYAFCHRCRGDADGKPAWADAAHGDEVPYVFGVPMGGPTEAFACNFSRSDVMLSAVVMTYWTNFAKTGDPNRPVPQDTKFIHTRPNRFEEVAWSKYDPREQLYLHIGLRPRVRDHYRATKVAFWLELVPHLHGLRDLLQYVSSTTRAPDDTFRAAKDTPRAAKDSRGGDDSSADARSRTHAKPWPATRRPPAVTRPRSGPRPAAAAEDATVLIETKRDYATELSVTIAVGASLLFLNILAFAALYYKKDKRRHETHRRQHHGAAVHAHTHTQRGGGGGGGAHGRADDFLQAKPPLPPPPLPPQRHDGGGGVGGGDAGLDAATAGGIRVSGPPDYALTLRRSPDDIPLMAPSTITMAPNAAAAGGSSGALHAFGAFAGGGGGVGGQNGSNLPHGHSTTRV

>Glis_glis_this_paper

MSRPRGWLWLPLCVTPVCGTMLNCNVAVWITVLVLQCSLLDGQAQHPVVSTNYGKLRGLRTPLPSEILGPVEQYLGVPYASPPTGDRRFQPPEPPSSWTGVRNATQFAAVCPQHLDERALLHDMLPVWFTANLDTLMTYVQDQNEDCLYLNIYVPTEDGANTKKIADDIASNERGDDEDIHDQSSKKPVMVYIHGGSYMEGTGNMVDGSILASYGNVIVITINYRLGILGFLSTGDQAAKGNYGLLDQIQALRWIEENVGAFGGDPKRVTIFGSGAGASCVSLLTLSHYSEGLFQKAIIQSGTALSSWAVNYQPAKYTRILADKVGCNMLDTTDLVECLRHKPAKELTQQAITPATYHIAFGPVIDGDVIPDDPQILMEQGEFLNYDIMLGVNQGEGLKFVDGIVDGEDGVTPNDFDFSVSNFVDNLYGYPEGKDTLRETIKFMYTDWADKENPETRRKTLVALFTDHQWVAPAVATADLHAQYGSPTYFYAFYHHCQSDMKPSWADSAHGDEVPYVFGIPMIGPTDLFSCNFSKNDVMLSAVVMTYWTNFAKTGDPNQPVPQDTKFIHTKPNRFEEVAWSKYNPKDQLYLHIGLKPRVRDHYRATKVAFWLELVPHLHNLNEIFQYVSTTTKVPPPDMTSFPYGTRRSPAKVWPTTKRPAITPANPKNSKDPHKTGPPEDTTVLIETKRDYSTELSVTIAVGASLLFLNILAFAALYYKKDKRRHETHRRPSPQRNATNDIAHLQGDDIMSLQMKQLEHDHDCESLQAHDALRLTCPPDYALTLRRSPDDIPLMTPSTITMIPSTLSAMQPLHTFNTFSAGQNSTNLPHGHSTTRV

>Sigmodon_hispidus_(Maxeiner et al., 2020)

MYARVPAAPVLLWALSAAASVDPRGVWPVVSTNYGRLRGLRVSLPGDVLGPVEQFLGVPYAAPPTGARRFQPPEPPSSWPGVRNATRFAPVCPQHLDERALLRDMLPVWFTANLDTLAAYVQEQSEDCLFLNVYAPAGGANGRKLADDITSNERGDDEDAHDPGGRKPVMVYIHGGSYMEGTGNMIDGSILASYGNVIVITVNYRLGVLGFLSTGDQAAKGNYGLLDQIQALRWVEENVGAFGGDPKRVTIFGSGAGASCVSLLTLSHYSEGLFQKAIIQSGTALSSWAVNYQPAKYTRALADRVGCNMLDTAELVECLRRQGWRELTREHVMPATFHVAFGPVIDGDVIPDDPQILMEQGEFLNYDIMLGVNQGEGLRFLEAAVGDPAVRDPATAHAEPESALGVSAADAESALGVSAGEFDLAVSSFVDGLYGYPEGKDTLRETIKFMYTDWADRDNPETRRKTLVALFTDHQWVAPAVATADLHAQYGSPTYFYAFYHHCQSDMKPPWADAAHGDEVPYVFGVPMLGPTELFACNFSKNDVMLSAVVMTYWTNFAKTGDPNQPVPQDTKFIHTKPNRFEEVAWSKYSPREQLYLHIGLKPRVRDHYRATKVAFWLELVPHLHGLHDLLQYVSTTTRAPPSSPDAAAAGAAIVPYGSAPHRRAWPATRRPAVSAAGNSGGSRDAHSRNGNAGVSRASGGSGGGGSAHPEEAAVLIETKRDYSTELSVTIAVGASLLFLNILAFAALYYKKDKRRHETHRRHHHQGRAAPEAASGAGDCGRKKPPRGDPARGDDARPATAAVAAPPDYALTLRRSPDDIPLMAAAAAAATPNTITMATGNMAGVSGVTASLLTFAAAQSGANLPHGHGGHSTTRV

>Marmota_flaviventris_(Maxeiner et al., 2020)

MSRPKGLLWLPLFFTPICVMLNSNVLLWITALAIKFTLIDSQAQYPVVNTNYGKIRGLRTPLPNEILGPVEQYLGVPYASPPTGERRFQPPEPPSSWTGVRNATQFAAVCPQHLDERSLLHDMLPIWFTANLDTLMTYVQDQNEDCLYLNIYVPTEDGANTKKIADDITSNDRGDDEDIHDQSSKKPVMVYIHGGSYMEGTGNMIDGSILASYGNVIVITINYRLGILGFLSTGDQAAKGNYGLLDQIQALRWIEENVGAFGGDPKRVTIFGSGAGASCVSLLTLSHYSEGLFQKAIIQSGTALSSWAVNYQPAKYTRLLADKVGCNMLDTTDLVECLRNKNHKELIQQAITPATYHIAFGPVIDGDVIPDDPQILMEQGEFLNYDIMLGVNQGEGLKFVDGIVDNEDGVTPNDFDFSVSNFVDNLYGYPEGKDTLRETIKFMYTDWADKENPETRRKTLVALFTDHQWVAPAVATADLHAQYGSPTYFYAFYHHCQSEMKPSWADSAHGDEVPYVFGIPMIGPTELFSCNFSKNDVMLSAVVMTYWTNFAKTGDPNQPVPQDTKFIHTKPNRFEEVAWSKYNPKDQLYLHIGLKPRVRDHYRATKVAFWLELVPHLHNLNEIFQYVSTTTKVPPPDMTSFPYGTRRSPAKIWPTTKRPAITPANSPKHSKDSHKTGPEDTTVLIETKRDYSTELSVTIAVGASLLFLNILAFAALYYKKDKRRHETHRRPSPQRNTTNDIAHIQNEEILSLQMKQLDHDHECDSLQAHDTLRLTCPPDYTLTLRRSPDDIPLMTPNTITMIPNTLTGMQPLHTFNTFSGGQNSTNLPHGHSTTRV

>Lepus_timidus_this_paper

MMPQPAGLPLPLLPLFSGPAPALWLATLSIHIAVMDGQAQGPVVSTNYGKVRGLRATLPGEVLGPVDQFLGVPYAAPPTGERRFQPPEPPSSWAGVRNATRFPAVCPQHLDEASLLHDMLPVWFTANLDSLAAYLQDQSEDCLYLNLYVPAGSHTRKNTDEINNSERLEEGDAREQGGRKPVMVYIHGGSYLEGSGNMMDGSVLASYGDVIVITLNYRLGVLGFLSTGDQAAKGNYGLLDQIQALRWVEENVGAFGGDPKRVTVFGSGAGASCVSLLTLSHYSEGLFQKAIIQSGTALSSWAVNYQPAKSTRLLAERLGCGAGGDSAALVRCLRAREAEELVRQPGAMAPAPYHVAFGPVIDGDVIPDDPQILMEQGEFLNYDILLGVNQGEGLRFVAVDDVDVADVDFDGSVAAFVDRLYGTPGGRDALRETVKFMYTDWADRHDPEARRKTLVALFTDHQWVAPAVATADLHARYGSPTYFYAFYHRCHTDTRPAWAADAAHGDEVPYVFGVPLLGPADLFACNFSRNDVMLSAVVMTYWTNFAKTGDPNQPVPQDTKFIHTKPNRFEAVAWAKYSPREQLYLHIGLRPRVRDHYRATKVAFWLELVPHLHGLRELLQDASPTPTSIGARRSAGHAWPHFQDGGQDGTPIGARRSANNAWPGIQDGGQDGTPIGARRSASNAWPGIQDGSPIGTRRSMSNAWPGTQEGSQDGSAERPGSFRKAAAVLIETRRDYAAELSVTLAVGASLLFLNVVAFAALYYKRDRRRRVSQRRGGGADGSHAHRRDTSHTPKDVAAAAPCHSQDALRLGCPPDYALALRRAPEDPPPGAPSTITRGGGSGQGVASLPHGHSTTRV

>Oryctolagus_cuniculus_XP_008267490.1

MPQPAGLPLPLLPLLSGPAPVLWLAALSIHVAVMDGQAQGPVVSTNYGKVRGLRATLPGEVLGPVDQFLGVPYAAPPTGERRFQPPEPPSSWAGVRNATRFPAVCPQHLDEASLLHDMLPVWFTANLDSLAAYLQDQSEDCLYLNLYVPAGSHTRKNADEINNSERLEEGDAREQGGRKPVMVYIHGGSYLEGSGNMMDGSVLASYGDVIVITLNYRLGVLGFLSTGDQAAKGNYGLLDQIQALRWVEENVGAFGGDPKRVTVFGSGAGASCVSLLTLSHYSEGLFQKAIIQSGTALSSWAVNYQPAKSTRLLAERLGCGAGGDSAALVRCLRAREAEELVRQPGAVAPAPYHVAFGPVIDGDVIPDDPQILMEQGEFLNYDILLGVNQGEGLRFVAVDDVDVADVDFDGSVAAFVDRLYGAPGGRDALRETVKFMYTDWADRHDPEARRKTLVALFTDHQWVAPAVATADLHARYGSPTYFYAFYHRCHTDTRPAWAADAAHGDEVPYVFGVPLLGPADLFACNFSRNDVMLSAVVMTYWTNFAKTGDPNQPVPQDTKFIHTKPNRFEAVAWAKYSPREQLYLHIGLRPRVRDHYRATKVAFWLELVPHLHGLRELLQDASPTPTSIGARRSAGHAWPHFQDGGQDGTPIGARRSASNAWPGIQDGGQDGTPIGARRSASNAWPGIQDGSPIGTRRSMSNAWPGTQEGSQDGSAERPGSFRKAAAVLIETRRDYAAELSVTLAVGASLLFLNVVAFAALYYKRDRRRRVSQRRGGGADGGHAHRRDASHTPKDVAAAAAPCHSQDALRLGCPPDYALALRRAPEDPPPGAPSTITRGGGSGQGVSSLPHGHSTTRV

>Dipodomys_spectabilis_a_this_paper

MEGRVAWWTWTLCLVVTPSTLGGQGSEDDPVVRTQYGHLRGLRASLPSELLGPVQQFLGIPYAAPPVGPRRFLPPEPPTAWPGIRNATHFAPVCPQRLDERTLPRDMLPSWLSANLETVAGLLREQSEDCLFLNVYVPTEDDIHEPGARRPVMVYIHGGSYMEGTGNMIDGSVLASYGNVIVITLNYRLGILGFLSTGDQAAKGNYGLLDQIQALRWVEENVGAFGGDPKRVTIFGSGAGASCVSLLTLSHYSEGLFQKAIIQSGTALSSWAVNYQPARYARALAAQLGCPSPADTSALVSCLRLKSPQELTRPVVTPATYHVAFGPVIDGDVIPDDPQILMEQGEFLNYDILLGVNQGEGLGFVDGLVDALDDGVSAAAFEASIAAFVDHLYGYPEGKQALRETIKFMYTDWADRDNPETRRKTLVALFTDHQWVAPAVATADLHAQYGSPTYFYAFYHRCQSELKPAWADAAHGDEVPYVFGVPMVGPTELFSCNFSKNDVMLSAVVMTYWTNFAKTGDPNQPVPQDTKFIHTKPNRFEEVAWSKYNPRDQLYLHIGLKPRVRDHYRATKVAFWLELVPHLHNLNDILQYVSTTTTRAPDVTSSSSSSHPRRATKRPTSSSSSILGPKALRPEGKLRQGGTEHSTTTVLIETKRDYSTELSVTIAVGASLLFLNILAFAALYYKKDKRRHQTHRRPSSPPSTSTRPPAPQDAAQRHLLRASAPTELLSVQLNPNVGAHEARDLQDALHLTCPPDYALTLRRSPDDIPLMTPSTITTPGPTLHTFNTFGGAGGGGVGGPGGGSGGQNNPLPHAHSTTRV

>Dipodomys_spectabilis_b_this_paper

MEGRVAWWTWTLCLVVTPSTLGGQGSEDDPVVRTQYGHLRGLRASLPSELLGPVQQFLGIPYAAPPVGPRRFLPPEPPTAWPGIRNATHFAPVCPQRLDERTLPRDMLPSWLSANLETVAGLLREQSEDCLFLNVYVPTEDDIHEPGARRPVMVYIHGGSYMEGTGNMIDGSVLASYGNVIVITLNYRLGILGFLSTGDQAAKGNYGLLDQIQALRWVEENVGAFGGDPKRVTIFGSGAGASCVSLLTLSHYSEGLFQKAIIQSGTALSSWAVNYQPARYARALAAQLGCPSPADTSALVSCLRLKSPQELTRPVVTPATYHVAFGPVIDGDVIPDDPQILMEQGEFLNYDILLGVNQGEGLGFVDGLVDALDDGVSAAAFEASIAAFVDHLYGYPEGKQALRETIKFMYTDWADRDNPETRRKTLVALFTDHQWVAPAVATADLHAQYGSPTYFYAFYHRCQSELKPAWADAAHGDEVPYVFGVPMVGPTELFSCNFSKNDVMLSAVVMTYWTNFAKTGDPNQPVPQDTKFIHTKPNRFEEVAWSKYNPRDQLYLHIGLKPRVRDHYRATKVAFWLELVPHLHNLNDILQYVSTTTTRAPDVTSSSSSSHPRRATKRPTSSSSSILGPKALRPEGKLRQGGTEHSTTTVLIETKRDYSTELSVTIAVGASLLFLNILAFAALYYKKDKRRHQTHRRPSSPPSTSTRTPAPQDAAQRHLLRASAPTELLSVQLNPNVGAHEARDLQDALHLTCPPDYALTLRRSPDDIPLMTPSTITTPGPTLHTFNTFGGAGGGGVGGPGGGSGGQNNPLPHAHSTTRV

>Arvicanthis_niloticus_this_paper

MPRPRAPRPPSVAWAWLWASCLCLAASGEAPPTPHPVVVTNYGKLRGVRTSPPTELLGPVDQFLGVPYAAPPTGERRFQPPEPPSSWAGTRNATRFAPVCPQPLDERALLRDMLPAWFTGNMDALAAYLAEQSEDCLFLNVYAPVDGADSRKSADDVAGRDRGDDEDPHDPGGRKPVMVYIHGGSYMEGTGNIMDGSVLASYGGVIVVTVNYRLGVLGFLSTGDQAAKGNYGLLDQIQALRWVEENAGAFGGDPKRVTVFGSGAGASCVSLLTLSHYSEGLFQKAIIQSGTALSSWAVNYQPARYARALGERVGCGSGADSTALVSCLRGKDAGELARAGVAPATYHVAFGPTVDGDVIPDDPQILMEQGEFLNYDIMLGVNQGEGARFVDGLDLDGGGDGDGDGEDGVDGGGGGVSAVAFDLAVAGFVDDLYGGGGAGGGDGGAALRETVKFMYTDWADRENPEARRKTLVALFTDHQWVAPAVATADLHARYGSPTYFYAFYHRCHGGGGGGGGGGGVGGGAGGGVAEAGGDAAHGDEVPYVFGVPMVGPTDVFGCNFSRNDVMLSAVVMTYWTNFAKTGDPNQPVPQDTKFIHTRPNRFEEVAWSKYDPREQLYLHIGLRPRVRDHYRATKVAFWLELVPHLHGLGDALQYLSTTTRAAPSDPDNMAAAATRRAPPPARARQATRRPAVTSSMTSSSVTSLGAAPPPGRGRGGDPGGASVLIETRRGGDYSTELSVTIAVGASLLFLNVLAFAALYYKKDKRRHESHRRTPGSAAGGSGGVGRPVSGPGSVRGAASGFGRGTSGFAGATSGPRGTSSGPGGSRGPTSGSGGLTSGLGHAPGAGRWPGNEASSDGMGNWRGGDCVKMAEGAGPGGVEGLRLTCPPDYALTLRRSPDDVPLAAATAAAAAPPPGAAITMIPGSLAATFNSFGPGSASSAAGSAPPGSGLPHGHSTTRV

>Muscardinus_avellanarius_this_paper

MSRPQGLARPPLTRPGVLLWLAALALSLSPGHGQAQYPVVSTNYGKIRGLRTPLPNEILGPVEQYLGVPYASPPTGDRRFQPPEPPSSWTGVRNATQFAAVCPQHLDERSLLHDMLPIWFTANLDTLMTYVQDQNEDCLYLNIYVPTEDGANTKKIADDITSNERGDDEDIHEQSSKKPVMVYIHGGSYMEGTGNMVDGSVLASYGNVIVVTVNYRLGVLGFLSTGDQAAKGNYGLLDQIQALRWIEENVGAFGGDPKRVTIFGSGAGASCVSLLTLSHYSEGLFQKAIIQSGTALSSWAVNYQPAKYARLLADRVGCNMLDTAELVDCLRARAHGDLVRPGVAPATYHVAFGPVIDGDVIPDDPQILMEQGEFLNYDIMLGVNQGEGLRFVDGLVDGEDGVAASDFDFSVSSFVDSLYGYPEGKDALRETIKFMYTDWADKDSAETRRKTLVALFTDHQWVAPAVATADLHAQYGSPTYFYAFYHHCQSDMKPAWADAAHGDEVPYVFGVPMLGPTELFSCNFSKNDVMLSAVVMTYWTNFAKTGDPNQPVPQDTKFIHTKPNRFEEVAWSKYNPKDQLYLHIGLKPRVRDHYRATKVAFWLELVPHLHNLHDIFQYVSTTTRAPPPDLTSFPAGSARRPSPAKAWPTTRRPALTPALPKAAPDRGDGGGTVLIETRRDYSTELSVTIAVGASLLFLNILAFAALYYKKDKRRHDSHRRPSPQRAAAAANDLARRHLQADDLVSLQMKPLEPPRAAGGDHDCEALPAHDALRLACPPDYTLTLRRSPDDVPLMAPSTITMIPNSLAAMPPLHTFNTFGGGGQNSSNLPHGHSTTRV

>Graphiurus_murinus_this_paper

MSRPTGLVWLPLFLPPLCGIVLNCKVLLWITALALNFTLADGQAQYPVVNTNYGKIRGLRTPLPNEILGPVEQYLGVPYASPPTGERRFQPPEPPSSWTGVRNATQFAAVCPQHLDERSLLHDMLPIWFTANLDTLMSYVQDQNEDCLYLNIYVPTEDGANTKKIADDITSNERGDDEDIHDQSNKKPVMVYIHGGSYMEGTGNMIDGSILASYGNVIVITINYRLGILGFLSTGDQAAKGNYGLLDQIQALRWIEENVGAFGGDPKRVTIFGSGAGASCVSLLTLSHYSEGLFQKAIIQSGTALSSWAVNYQPAKYTRILADKVGCNMLDTTDLVECLRGKGPRELVQQALTPATYHIAFGPVIDGDVIPDDPQILMEQGEFLNYDIMLGVNQGEGLKFVDGLVDGEDGVTPSDFDFAVSNFVDNLYGYPEGKDTLRETIKFMYTDWADKENPETRRKTLVALFTDHQWVAPAVATADLHAQYGSPTYFYAFYHHCQSDMKPGWADAAHGDEVPYVFGVPMLGPTDLFSCNFSKNDVMLSAVVMTYWTNFAKKGDPNQPVPQDTKFIHTKPNRFEEVAWSKYNPKDQLYLHIGLKPRVRDHYRATKVAFWLELVPHLHNLNEIFQYVSTTTKVPPPDMTSFPYGTRRSPAKTWPTTKRPALTPASHPKSSKEAHKPGPPDDTTVLIETKRDYSTELSVTIAVGASLLFLNILAFAALYYKKDKRRHETHRRPSPPPRSAQANDLAHLQGDELMSLQMKQLDHECESLQAHDTLRLTCPPDYTLTLRRSPDDIPLMTPNTITMIPNTLAAGGGMQPLHTFNTFSGGQNSTNLPHGHSTTRV

>Tupaia_chinensis_XP_006147843_plus_insert_A2

MSRPKGLLWVPLFFTPVCAMFNSNVLLWLAALTLKFMLIDGQAQYPVVNTNYGKIRGLRTPLPNEILGPVEQYLGVPYASPPTGERRFQPPEPPSSWTGVRNATQFAAVCPQHLDERSLLHDMLPVWFTANLDTLMTYAQDQKEDCLYLNIYVPTEDGANTKKNADDITSNDRGEDEDIHDQNSKKPVMVYIHGGSYMEGTGNMIDGSILASYGNVIVITINYRLGILGFLSTGDQAAKGNYGLLDQIQALRWVEENVGAFGGDPKRVTIFGSGAGASCVSLLTLSHYSEGLFQKAIIQSGTALSSWAVNYQPAKYTRILADKVGCNMLDTTDMVECLRNKNHKELIQQSITPATYHIAFGPVIDGDVIPDDPQILMEQGEFLNYDIMLGVNQGEGLKFVDGIVDNEDGVTPNDFDFSVSNFVDNLYGYPEGKDTLRETIKFMYTDWADKENPETRRKTLVALFTDHQWVAPAVATADLHAQYGSPTYFYSFYHHCQSEMKPSWADSAHGDEVPYVFGIPMIGPTELFSCNFSKNDVMLSAVVMTYWTNFAKTGDPNQPVPQDTKFIHTKPNRFEEVAWSKYNPKDQLYLHIGLKPRVRDHYRATKVAFWLELVPHLHTLNEIFQYVSTTTKVPPPDMTSFPYGTRRSPGKIWPTTKRPAITPANNPKHAKDPHKTGPEDTTVLIETKRDYSTELSVTIAVGASLLFLNILAFAALYYKKDKRRHETHRRPSPQRNTTNDIAHIQNEEIMSLQMKQLEQDHECASLQAHDTLRLTCPPDYTLTLRRSPDDIPLMTPNTITMIPNTLTGMQPLHTFNTFSGGQNSTNLPHGHSTTRV

>Galeopterus_variegatus_this_paper

MSRPKGLLWLPLFFTPVCVMLNSNVLLWITALAIKFTLIDSQAQYPVVNTNYGKIRGLRTPLPNEILGPVEQYLGVPYASPPTGERRFQPPEPPSSWTGVRNATQFAAVCPQHLDERSLLHDMLPIWFTANLDTLMTYVQDQNEDCLYLNIYVPTEDGANTKKNADDIASNDRGEDEDIHDQNSKKPVMVYIHGGSYMEGTGNMIDGSILASYGNVIVITINYRLGILGFLSTGDQAAKGNYGLLDQIQALRWIEENVGAFGGDPKRVTIFGSGAGASCVSLLTLSHYSEGLFQKAIIQSGTALSSWAVNYQPAKYTRILADKVGCNMLDTTDMVECLRNKNHKELIQQTITPATYHIAFGPVIDGDVIPDDPQILMEQGEFLNYDIMLGVNQGEGLKFVDGIVDNEDGVTPNDFDFSVSNFVDNLYGYPEGKDTLRETIKFMYTDWADKENPETRRKTLVALFTDHQWVAPAVATADLHAQYGSPTYFYAFYHHCQSEMKPSWADSAHGDEVPYVFGIPMIGPTELFSCNFSKNDVMLSAVVMTYWTNFAKTGDPNQPVPQDTKFIHTKPNRFEEVAWSKYNPKDQLYLHIGLKPRVRDHYRATKVAFWLELVPHLHNLNEIFQYVSTTTKVPPPDMTSFPYGTRRSPAKIWPTTKRPAITPANNPKHSKDPHKTGPEDTTVLIETKRDYSTELSVTIAVGASLLFLNILAFAALYYKKDKRRHETHRRPSPQRNTTNDIAHIQNEEIMSLQMKQLEHDHECESLQAHDTLRLTCPPDYTLTLRRSPDDIPLMTPNTITMIPNTLTGMQPLHTFNTFSGGQNSTNLPHGHSTTRV

>Tupaia_tana_this_paper

MSRPKGLLWVPLFFTPVCAMFNSNVLLWLAALTLKFTLIDGQAQYPVVNTNYGKIRGLRTPLPNEILGPVEQYLGVPYASPPTGERRFQPPEPPSSWTGVRNATQFAAVCPQHLDERSLLHDMLPVWFTANLDTLMTYVQDQNEDCLYLNIYVPTEDGANTKKNADDITSNDRGEDEDIHDQNSKKPVMVYIHGGSYMEGTGNMIDGSILASYGNVIVITINYRLGILGFLSTGDQAAKGNYGLLDQIQALRWIEENVGAFGGDPKRVTIFGSGAGASCVSLLTLSHYSEGLFQKAIIQSGTALSSWAVNYQPAKYTRILADKVGCNMLDTTDMVECLRNKNHKELIQQSITPATYHIAFGPVIDGDVIPDDPQILMEQGEFLNYDIMLGVNQGEGLKFVDGIVDNEDGVTPNDFDFSVSNFVDNLYGYPEGKDTLRETIKFMYTDWADKENPETRRKTLVALFTDHQWVAPAVATADLHAQYGSPTYFYAFYHHCQSEMKPSWADSAHGDEVPYVFGIPMIGPTELFSCNFSKNDVMLSAVVMTYWTNFAKTGDPNQPVPQDTKFIHTKPNRFEEVAWSKYNPKDQLYLHIGLKPRVRDHYRATKVAFWLELVPHLHNLNEIFQYVSTTTKVPPPDMTSFPYGTRRSPGKIWPTTKRPAITPANNPKHSKDPHKTGPEDTTVLIETKRDYSTELSVTIAVGASLLFLNILAFAALYYKKDKRRHETHRRPSPQRNTTNDIAHIQNEEIMSLQMKQLEQDHECASLQAHDTLRLTCPPDYTLTLRRSPDDIPLMTPNTITMIPNTLTGMQPLHTFNTFSGGQNSTNLPHGHSTTRV

>Callithrix_jacchus_XP_035145065.1

MSRPQGLLWLPLLFTPVCVVLNSNVLLWITALAIKFTLIDSQAQYPVVNTNYGKVRGLRTPLPNEILGPV

EQYLGVPYASPPTGERRFQPPEPPSSWTGIRNATQFAAVCPQHLDERSLLHDMLPIWFTANLDTLMTYVQ

DQNEDCLYLNIYVPTEDGANTKKNADDITSNDRGEDEDIHDQNSKKPVMVYIHGGSYMEGTGNMIDGSIL

ASYGNVIVITINYRLGILGFLSTGDQAAKGNYGLLDQIQALRWIEENVGAFGGDPKRVTIFGSGAGASCV

SLLTLSHYSEGLFQKAIIQSGTALSSWAVNYQPAKYTRILADKVGCNMLDTTDMVECLRNKNYKELIQQT

ITPATYHIAFGPVIDGDVIPDDPQILMEQGEFLNYDIMLGVNQGEGLKFVDGIVDNEDGVTPNDFDFSVS

NFVDNLYGYPEGKDTLRETIKFMYTDWADKENPETRRKTLVALFTDHQWVAPAVATADLHAQYGSPTYFY

AFYHHCQSEMKPSWADSAHGDEVPYVFGVPMIGPTELFSCNFSKNDVMLSAVVMTYWTNFAKTGDPNQPV

PQDTKFIHTKPNRFEEVAWSKYNPKDQLYLHIGLKPRVRDHYRATKVAFWLELVPHLHNLNEIFQYVSTT

TKVPPPDMTSFPYGTRRSPAKIWPTTKRPAITPANNPKHSKDPHKTGPEDTTVLIETKRDYSTELSVTIA

VGASLLFLNILAFAALYYKKDKRRHETHRRPSPQRNTTNDIAHIQNEEMMSLQMKQLEHDHECESLQAHD

TLRLTCPPDYTLTLRRSPDDIPLMTPNTITMIPNTLTGMQPLHTFNTFSGGQNSTNLPHGHSTTRV

>Y_Callithrix_jacchus_XP_035145772.1

MSRPKILLWLPLLFTPVCIMLKSNVLLWMTALAIKFTLIDSQAQYPVVNTNYGKVRGLRTPIPNEILGPV

EQYLGVPYASPPTGERRFQSPEPPSSWTGIRNATQFAAVCPQHLDERSLLHDMLPIWFTANLDTLMTYSQ

DQNEDCLYLNIYVPTENDIHDQNSKKPVMVYIHGGSYMEGTANIIDGSILASYGNVIVITINYRLGILGF

LSTGDQAAKGNYGLLDQIQALRWIEENVGAFGGDPKRVTIFGSGAGASCVSLLTLSHYSEGLFQKAIVQS

GTALSSWAVNYQPAKYTRILADKVGCNMLDTMDMVECLRNKNYKELIQQTITPATYHIAFGPVIDGDVIP

DDPQILMEQGEFLNYDIMLGVNQGEGLKFVDGIVDNEDGVTPNDFDFSVSNFVDNLYGYPEGKDTLRETI

KFMYTDWADKENPETRRKTLVALFTDHQWVAPAVATADLHAQYGSPTYFYAFYHHCQSEMKPSWADSAHG

DEVPYVFGIPMIGPTELFSCNFSKNDVMLSALVMTYWTNFAKTGDPNQPVPQDTKFIHMKPNRFEEVAWS

KYNPKDQLYLHIGLKPRVRDHYRATKVAFWLELVPHLHNLNEIFQYVSTTTKVPPPYMTSFPYGTWRSPT

KIWPTTKRPAITPANNPKHSKDPHKTGPEDTTVLIETKRDYSTELSITIAVGASLLFLNILAFAALYYKK

DKRRHETHRRPSPQRNTTNNIAHIQNEEMMSLQMRQLEHDHECASLQAHDTLRLTCPPDYTLTLHQSSDD

ILLMTPNTITMIPNTLTRMQPLHTFNTFSGGQNSTNLPHGHSTTRV

>Carlito_syrichta_this_paper

MSRPKGLLWLPLFFTPVCIMLNSNVLLWITALAIKFTLIDSQAQYPVVNTNYGKIRGLRTPLPNEILGPVEQYLGVPYASPPTGERRFQPPEPPSSWTGVRNATQFAAVCPQHLDERSLLHDMLPIWFTANLDTLMTYVQDQNEDCLYLNIYVPTEDGANTKKNADDITSNDRGEDEDIHDQNSKKPVMVYIHGGSYMEGTGNMIDGSILASYGNVIVITINYRLGILGFLSTGDQAAKGNYGLLDQIQALRWIEENVGAFGGDPKRVTIFGSGAGASCVSLLTLSHYSEGLFQKAIIQSGTALSSWAVNYQPAKYTRILADKVGCNMLDTTDMVECLRNKNYKELIQQTITPATYHIAFGPVIDGDVIPDDPQILMEQGEFLNYDIMLGVNQGEGLKFVDGIVDNEDGVTPNDFDFSVSNFVDNLYGYPEGKDTLRETIKFMYTDWADKENPETRRKTLVALFTDHQWVAPAVATADLHAQYGSPTYFYAFYHHCQSEMKPSWADSAHGDEVPYVFGIPMIGPTELFSCNFSKNDVMLSAVVMTYWTNFAKTGDPNQPVPQDTKFIHTKPNRFEEVAWSKYNPKDQLYLHIGLKPRVRDHYRATKVAFWLELVPHLHNLNEIFQYVSTTTKVPPPDMTSFPYGTRRSPPKIWPTTKRPAITPANNPKHSKDPHKTGPEDTTVLIETKRDYSTELSVTIAVGASLLFLNILAFAALYYKKDKRRHETHRRPSPQRNTTNDIAHIQNEEIMSLQMKQLEHDHECESLQAHDTLRLTCPPDYTLTLRRSPDDIPLMTPNTITMIPNTLTGMQPLHTFNTFSGGQNSTNLPHGHSTTRV

>Lepus_americanus_americanus

MMPQPAGLPLQLLPLFSGPAPALWLATLSIHIAVMDGQAQGPVVSTNYGKVRGLRATLPGEVLGPVDQFLGVPYAAPPTGERRFQPPEPPSSWAGVRNATRFPAVCPQHLDEASLLHDMLPVWFTANLDSLAAYLQDQSEDCLYLNLYVPAGSHTRKNTDEINNSERLEEGDAREQGGRKPVMVYIHGGSYLEGSGNMMDGSVLASYGDVIVITLNYRLGVLGFLSTGDQAAKGNYGLLDQIQALRWVEENVGAFGGDPKRVTVFGSGAGASCVSLLTLSHYSEGLFQKAIIQSGTALSSWAVNYQPAKSTRLLAERLGCGAGGDSAALVRCLRAREAEELVRQPGAMAPAPYHVAFGPVIDGDVIPDDPQILMEQGEFLNYDILLGVNQGEGLRFVAVDDVDVADVDFDGSVAAFVDRLYGAPGGRDALRETVKFMYTDWADRHDPEARRKTLVALFTDHQWVAPAVATADLHARYGSPTYFYAFYHRCHTDTRPAWAADAAHGDEVPYVFGVPLLGPADLFACNFSRNDVMLSAVVMTYWTNFAKTGDPNQPVPQDTKFIHTKPNRFEAVAWAKYSPREQLYLHIGLRPRVRDHYRATKVAFWLELVPHLHGLRELLQDASPTPTSIGARRSAGHAWPHFQDGSQDGTPIGARRSANNAWPGIQDGGQDGTPIGARRSASNAWPGIQDGSPIGTRRSMSNAWPSTQEGSQDGSAERPGSFRKAAAVLIETRRDYAAELSVTLAVGASLLFLNVVAFAALYYKRDRRRRVSQRRGGGADGSHAHRRDTSHTPKDVAAAAPCHSQDALRLGCPPDYALALRRAPEDPPPGAPSTITRGGGSGQGVASLPHGHSTTRV

>Sylvilagus_bachmani_this_paper

MPRPAGLPLLSGPAPALWLAALSIQVAVMDGQAQGPVVSTNYGKVRGLRATLPGEVLGPVDQFLGVPYAAPPTGERRFQPPEPPSSWAGVRNATRFPAVCPQHLDEASLLHDMLPVWFTANLDSLAAYLQDQSEDCLYLNLYVPAGSHTRKNADEINNSERLEEGDAREQGGRKPVMVYIHGGSYLEGSGNMMDGSVLASYGDVIVITLNYRLGVLGFLSTGDQAAKGNYGLLDQIQALRWVEENVGAFGGDPKRVTVFGSGAGASCVSLLTLSHYSEGLFQKAIIQSGTALSSWAVNYQPAKSTRLLAERLGCAAGGDSAALVRCLRAREAEELVRQPGAMAPAPYHVAFGPVIDGDVIPDDPQILMEQGEFLNYDILLGVNQGEGLRFVAVDDVDVADVDFDGSVAAFVDRLYGAPGGRDALRETVKFMYTDWADRHDPEARRKTLVALFTDHQWVAPAVATADLHARYGSPTYFYAFYHRCHTDTRPAWAADAAHGDEVPYVFGVPLLGPADLFACNFSRNDVMLSAVVMTYWTNFAKTGDPNQPVPQDTKFIHTKPNRFEAVAWAKYSPREQLYLHIGLRPRVRDHYRATKVAFWLELVPHLHGLRELLQDASPTPTSIGTRRSGGHAWPHFQDGGQDGTPIGARRSASNAWPGIQDGGQDGTPIGARRSGSNAWPGIQDGSPIGTRRSMSNAWPGTQEGSQDGSAERPGSFRKAAAVLIETRRDYAAELSVTLAVGASLLFLNVVAFAALYYKRDRRRRVSQRRGGGADGGHAHRRDAGHTPKDVAAAAPCHSQDALRLGCPPDYALALRRAPEDPPPGAPSTITRGGGSGQGVANLPHGHSTTRV

>Ondatra_zibethicus_(Maxeiner et al., 2020)

MARPLPAPWPLAAALACCAWAWASPAAPVVTTRYGRLRGLRAPPPGEALGPVARFLGVPYAAPPIGERRFQPPEPPHPWPGVRDATRFAPACPQPLDARMAMPRDMMPAWFAANLDAHVRDQSEDCLFLNVYAPADARGTRPVMVYIHGGSYTEGTANLIDGSVLASYGDVIVVTVNYRLGVLGFLSTGDQAARGNYGLLDQIQALRWVAENAGAFGGDPGRVTVFGSGAGASCVSLLTLSHYAEGLFQKAIIQSGTALSSWAVNYEPARHARALAARVGCARRDAAALVSCLRGAGWRELSRRRSAAAVAAAAAYRVAFGPVVDGDVVPDDPQVLMEQGEFLNYDVMLGVNQAEAMRLADALVGTGDTYDTNDSDPTYDANGADTTYDAKYMDPDASEDDMRVGGGDTDPNDGDMHVGDGDTYVGGSDTHVGGDTDPDDSDMYVGGGDMYVGGSNMHVGGGDTYVGGGDTHVDDDDTHVGGGVSPGAFAHAVSSFVDRLYGAPEGADALRETVKFMYTDWAARGSAWARRRALVALLTDHQWVAPAVATADLHARYGSPTYFYAFARRCPRAHARDDAAADAAAHGDEVPYVFGAPMAAAGDAFASCNFSRDDVMLSAAVMTYWTNFAKTGDPNRPVPQDTTFSHTRPSRFEAVAWAKYDPRGQLYLHLGLRPRVRDHYRAAKVAFWLELVPRLHGLHDLLSSVTVATRDPEHAKPRGAATRVPEHTKPRSNTRVPATRVPAPRATVPTRGHAVLIETRRDYATELSVTLAVGASLLLLNVLAFAALYYKKDRRRRETHRRLRGHGHGVVTAAHAKPPPPPPPPPHRGDYALALRRPPDDDAAAHGHAATRV

>Rhombomys_opimus_this_paper

MPLPWGLSWLTRRAPSDVTVSLMTSVIPAFEHKALRVMKTVTPIVTSSMVKSVVASMTSGALLACLAVASLAACVSCSSLPPEDSGEGAGPVVVSTRYGRLRGMRVPLPGGSLGPVARFLGVPYAAPPTGPRRFQPPEPPAPWPGVRGASQFAPVCPQDADSRPDPAAMLPAWLAADPDAVAAHAREQDEDCLYLNLYVPAGVGGHLRNLADDPSSDERGDDRDTRDPATRKPVMVFIHGDSYMAGTGNMMDGSVLASYGDVIVVTLNYRLGALGFLSTGDPAARGNYGLLDQMQALRWLRENAVAFGGDPARVTVFGSGAGASCVSLLTLSHYSEGLFQKAIIQSGTALSSWAVNYQPATYARMLGARVGCGGDMTSATSPPDTAATPPLTSSVHDPPSPSAALVACLRRRGARELTRAAGSVPASAPFHVAFGPVIDGDVVPDDPQILMEQGEFLNYDILLGVNQAEGVALADPAHPDGGGDITAEGEEEEEVSAAGFELAVAAFVDALYGYPGGEVGVAGLGGGVAGWGGGAGGDSALRETARFMYTDWAEREGGAGSRRRALAAMMTDHQWAAPAVATADLHARYGSATYFYAFAHPCRGDAHPTWAAEAGAAHGDELPFVFGVPMLVLAAAGDGSVGGVGGEGATGTDVAAATAAALFPCNFTRNDVMLSAVVMTYWTNFAKTGDPNRPVPQDTKFAHTRPNRFEAVAWPKYTPRERLYLHVGLRPRVRDHYRATKVAFWLELVPHLHGLREAFPYLTTPTAAPHVPTGPRRVWPPTRRPALPSLGRPASASSSSSQDSKVGPGEAVLIETRRDYSTELSVTIAVGASLLFLNILAFAALYYKKDRRRHETHHRRMAASGATSGFASASGTASGPTSDFVSASGHASGPASGFASTSGPTSGFASTHCPGNDTGKRGREENPGEMVTSSVAPPSSLDASLRLPTGPPDYTLTLRRAPDDAPPLTAPSAITMVPNALAGLPQLHTFGHSTTRV

>Xerus_inauris_this_paper

MSNPKGLLWLPLFFTPVCVMLNSNVLLWLTALAIKFTLIDCQAQYPVVNTNYGKIRGLRTPLPNEILGPVEQYLGVPYASPPTGERRFQPPEPPSSWTGVRNATQFAAVCPQHLDERSLLHDMLPIWFTANLDTLMTYVQDQNEDCLYLNIYVPTEDGANTKKIADDITSNDRGDDEDIHDQSSKKPVMVYIHGGSYMEGTGNMIDGSILASYGNVIVITINYRLGILGFLSTGDQAAKGNYGLLDQIQALRWIEENVGSFGGDPKRVTIFGSGAGASCVSLLTLSHYSEGLFQKAIIQSGTALSSWAVNYQPAKYTRILADKVGCNMLDTTDMVECLRVKNYKELIQQAITPATYHIAFGPVIDGDVIPDDPQILMEQGEFLNYDIMLGVNQGEGLKFVDGIVDNEDGVTPNDFDFSVSNFVDNLYGYPEGKDTLRETIKFMYTDWADKENPETRRKTLVALFTDHQWVAPAVATADLHAQYGSPTYFYAFYHHCQSEMKPSWADSAHGDEVPYVFGIPMIGPTELFSCNFSKNDVMLSAVVMTYWTNFAKTGDPNQPVPQDTKFIHTKPNRFEEVAWSKYNPKDQLYLHIGLKPRVRDHYRATKVAFWLELVPHLHNLNEIFQYVSTTTKVPPPDMTSFPYGTRRSPAKIWPTTKRPAITPANNPKHSKDSHKTGPEDTTVLIETKRDYSTELSVTIAVGASLLFLNILAFAALYYKKDKRRHETHRRPSPQRNTTNDIAHIQNEEIMSLQMKQLEHDHECESLQAHDTLRLTCPPDYTLTLRRSPDDIPLMTPNTITMIPNTLTGMQPLHTFNTFSGGQNSTNLPHGHSTTRV

>Ctenodactylus_gundi_this_paper

MPRAKGLLWLPLFCTPVCVVVNSNVLLWITVLAIKFTLIDSQAQYPMVNTNYGKIRGLRTPLPNEILGPVEQYLGVPYASPPTGERRFQPPEPPSSWTGVRNATQFAAVCPQHLDDRSLLHDMLPVWFTANLDTLMTYVQDQNEDCLYLNIYVPTEDGANSKKLADDITSNDRGADEDIHDQNSKKPVMVYIHGGSYMEGTGNMIDGSILASYGNVIVITINYRLGILGFLSTGDQAAKGNYGLLDQIQALRWIEENVGAFGGDPKRVTIFGSGAGASCVSLLTLSHYSEGLFQKAIIQSGTALSSWAVNYQPAKYTRILADKVGCNMLDTTDMVECLRGKNYKELIQQAITPATYHIAFGPVIDGDVIPDDPQILMEQGEFLNYDIMLGVNQGEGLKFVDGIVDNEDGVTPNDFDFSVSNFVDNLYGYPEGKDTLRETIKFMYTDWADKENPETRRKTLVALFTDHQWVAPAVATADLHAQYGSPTYFYAFYHHCQSEMKPTWADSAHGDEVPYVFGIPMIGPTELFSCNFSKNDVMLSAVVMTYWTNFAKTGDPNQPVPQDTKFIHTKPNRFEEVAWSKYNPKDQLYLHIGLKPRVRDHYRATKVAFWLELVPHLHNLNEIFQYVSTTTKVPPPDMTSFPYGTRRSPAKIWPTTKRPAITPANNPKHSKDPHKTAPEDTTVLIETKRDYSTELSVTIAVGASLLFLNILAFAALYYKKDKRRHETHRRPSPQRNTTNDIAHIQNEEIMSLQMKQLEQDHECESLQAHDTLRLTCPPDYTLTLRRSPDDIPLMTPNTITMIPNTLTGMQPLHTFNTFSGGQNSTNLPHGHSTTRV

>Ctenomys_sociabilis_this_paper

MWKRTMSRPKGLIWVPLFCTPVCVMVNSNVLLWITALAIKFTLIDSQAQYPVVNTNYGKIRGLRTPLPNEILGPVEQYLGVPYASPPTGERRFQPPEPPSSWTGVRNATQFAGVCPQHLDERSLLHDMLPIWFTANLDTLMTYVQDQNEDCLYLNIYVPTEDGANTKKIADDITSNDRADDEDIHDQNSKKPVMVYIHGGSYMEGTGNMIDGSILASYGNVIVITINYRLGILGFLSTGDQAAKGNYGLLDQIQALRWVEENVAAFGGDPKRVTIFGSGAGASCVSLLTLSHYSEGLFQKAIIQSGTALSSWAVNYQPAKYTRMLADKVGCNMLDTTDLVECLRNKNYKELIQQAITPATYHIAFGPVIDGDVIPDDPQILMEQGEFLNYDIMLGVNQGEGLKFVDGIVDNEDGVTPNDFDFSVSNFVDNLYGYPEGKDTLRETIKFMYTDWADKENPETRRKTLVALFTDHQWVAPAVATADLHAQYGSPTYFYAFYHHCQSEMKPSWADSAHGDEVPYVFGIPMIGPTELFSCNFSKNDVMLSAVVMTYWTNFAKTGDPNQPVPQDTKFIHTKPNRFEEVAWSKYNPKDQLYLHIGLKPRVRDHYRATKVAFWLELVPHLHNLNEIFQYVSTTTKVPPPDMTSYPYGTRRSPAKIWPTTKRPAITPANNPKHSKDSQKTGPEDTTVLIETKRDYSTELSVTIAVGASLLFLNILAFAALYYKKDKRRHETHRRPSPQRSTTNDIAHMQNEEIMSLQMKQLEHDHECESLQAHDTLRLTCPPDYALTLRRSPDDIPLMTPNTITMVPSTLTGMQPLHTFNTFSGGQNSTNLPHGHSTTRV

>Dasyprocta_punctata_this_paper

MWKRTMSRPKGLLWLPLFCTPVCVMVNSNVLLWITALAIKFTVIDSQAQYPVVNTNYGKIRGLRTPLPNEILGPVEQYLGVPYASPPTGERRFQPPEPPSSWTGVRNATQFAAVCPQHLDERSLLHDMLPIWFTANLDTLMTYVQDQNEDCLYLNIYVPTEDGANTKKIADDITSNDRGDDEDIHDQNSKKPVMVYIHGGSYMEGTGNMIDGSILASYGNVIVITINYRLGILGFLSTGDQAAKGNYGLLDQIQALRWIEENVAAFGGDPKRVTIFGSGAGASCVSLLTLSHYSEGLFQKAIIQSGTALSSWAVNYQPAKYTRMLADKVGCNMLDTTDMVECLRNKNYKELIQQAITPATYHIAFGPVIDGDVIPDDPQILMEQGEFLNYDIMLGVNQGEGLKFVDGIVDNEDGVTPNDFDFSVSNFVDNLYGYPEGKDTLRETIKFMYTDWADKENPETRRKTLVALFTDHQWVAPAVATADLHAQYGSPTYFYAFYHHCQSEMKPSWADSAHGDEVPYVFGIPMIGPTELFSCNFSKNDVMLSAVVMTYWTNFAKTGDPNQPVPQDTKFIHTKPNRFEEVAWSKYNPKDQLYLHIGLKPRVRDHYRATKVAFWLELVPHLHNLNEIFQYVSTTTKVPPPDMTSFPYGTRRSPAKIWPTTKRPAITPANNPKHAKDLHKTGPEDTTVLIETKRDYSTELSVTIAVGASLLFLNILAFAALYYKKDKRRHETHRRPSPQRNTTNDIAHIQNEEIMSLQMKQLEHDHECESLQAHDTLRLTCPPDYTLTLRRSPDDIPLMTPNTITMIPNTLTGMQPLHTFNTFSGGQNSTNLPHGHSTTRV

>Dinomys_branickii_this_paper

MWNRTMSRPKGLLWLPLFCTPVCIMVNSNVLLWITALAIKFTVIDSQAQYPVVNTNYGKIRGLRTPLPNEILGPVEQYLGVPYASPPTGERRFQPPEPPSSWTGVRNATQFAAVCPQHLDERSLLHDMLPIWFTANLDTLMTYVQDQNEDCLYLNIYVPTEDGANTKKIADDITSNDRGDDEDIHDQNSKKPVMVYIHGGSYMEGTGNMIDGSILASYGNVIVITINYRLGILGFLSTGDQAAKGNYGLLDQIQALRWIEENMPAFGGDPKRVTIFGSGAGASCVSLLTLSHYSEGLFQKAIIQSGTALSSWAVNYQPAKYTRMLADKVGCNMLDTTDMVECLRNKNYKELIQQAITPATYHIAFGPVIDGDVIPDDPQILMEQGEFLNYDIMLGVNQGEGLKFVDGIVDNEDGVTSNDFDFSVSNFVDNLYGYPEGKDTLRETIKFMYTDWADKENPETRRKTLVALFTDHQWVAPAVATADLHAQYGSPTYFYAFYHHCQSEMKPSWADSAHGDEVPYVFGIPMIGPTELFSCNFSKNDVMLSAVVMTYWTNFAKTGDPNQPVPQDTKFIHTKPNRFEEVAWSKYNPKDQLYLHIGLKPRVRDHYRATKVAFWLELVPHLHNLNEIFQYVSTTTKVPPPDMTSFPYGTRRSPAKIWPTTKRPAITPANNPKHSKDTQKTAPDDTTVLIETKRDYSTELSVTIAVGASLLFLNILAFAALYYKKDKRRHETHRRPSPQRNTTNDIAHIQNEEIMSLQMKQLEHDHECESLQAHDTLRLTCPPDYTLTLRRSPDDIPLMTPNTITMIPNTLTGMQPLHTFNTFSGGQNSTNLPHGHSTTRV

>Erethizon_dorsatum_this_paper

MWKRTMPRSKGLLWFPLFCTPVCIMVNSNVLLWITALAIKFTLIDSQAQYPVVNTNYGKIRGLRTPLPNEILGPVEQYLGVPYASPPTGERRFQPPEPPSSWTGVRNATQFAAVCPQHLDERSLLHDMLPIWFTANLDTLMTYVQDQNEDCLYLNIYVPTEDGANTKKIADDITSNDRGDDEDIHDQNSKKPVMVYIHGGSYMEGTGNMIDGSILASYGNVIVITINYRLGILGFLSTGDQAAKGNYGLLDQIQALRWIEENVGAFGGDPKRVTIFGSGAGASCVSLLTLSHYSEGLFQKAIIQSGTALSSWAVNYQPAKYTRMLADKVGCNMLDTTDMVECLRNKNYKELIQQAITPATYHIAFGPVIDGDVIPDDPQILMEQGEFLNYDIMLGVNQGEGLKFVDGIVDNEDGVTPNDFDFSVSNFVDNLYGYPEGKDTLRETIKFMYTDWADKENPETRRKTLVALFTDHQWVAPAVATADLHAQYGSPTYFYAFYHHCQSEMKPSWADSAHGDEVPYVFGIPMIGPTELFSCNFSKNDVMLSAVVMTYWTNFAKTGDPNQPVPQDTKFIHTKPNRFEEVAWSKYNPKDQLYLHIGLKPRVRDHYRATKVAFWLELVPHLHNLNEIFQYVSTTTKVPPPDITSFPYGTRRSPAKIWPTTKRPAITPANNPKHSKDLQKTGPEDTTVLIETKRDYSTELSVTIAVGASLLFLNILAFAALYYKKDKRRHETHRRPSPQRNATNDIAHIQNEEIMSLHMKQLEHDHECESLQAHDTLRLTCPPDYTLTLRRSPDDIPLMTPNTITMIPNTLTGMQPLHTFNTFSGGQNSTNLPHGHSTTRV

>Hystrix_brachyura_this_paper

MWKGAMSRPKGLLWFPLFCTPVCIMVNSNVLLWITALALKFTLTDSQAQYPVVNTNYGKIRGLRTPLPNEILGPVEQYLGVPYASPPTGERRFQPPEPPSSWTGVRNATQFAAVCPQHLDERSLLHDMLPIWFTANLDTLMTYVQDQNEDCLYLNIYVPTEDGANTKKIADDITSIDRDGDEDIHDQNSKKPVMVYIHGGSYMEGTGNMIDGSILASYGNVIVITINYRLGILGFLSTGDQAAKGNYGLLDQIQALRWIEENVGAFGGDPKRVTIFGSGAGASCVSLLTLSHYSEGLFQKAIIQSGTALSSWAVNYQPAKYTRMLADKVGCNMLDTTDMVECLRNKNYKELIQQAITPATYHIAFGPVIDGDVIPDDPQILMEQGEFLNYDIMLGVNQGEGLKFVDGIVDNEDGVTPNDFDFSVSNFVDNLYGYPEGKDTLRETIKFMYTDWADKENPETRRKTLVALFTDHQWVAPAVATADLHAQYGSPTYFYAFYHHCQSEMKPSWADSAHGDEVPYVFGIPMIGPTELFSCNFSKNDVMLSAVIMTYWTNFAKTGDPNQPVPQDTKFIHTKPNRFEEVAWSKYNPKDQLYLHIGLKPRVRDHYRATKVAFWLELVPHLHNLNEIFQYVSTTTKVPPPDMTSFPYGTRRSPAKIWPTTKRPAITPANNPKHSKDPHKTGPEDTTVLIETKRDYSTELSVTIAVGASLLFLNILAFAALYYKKDKRRHETHRRPSPQRNTTNDIAHIQNEEIMSLQMKQLEHDHECESLQAHDTLRLTCPPDYTLTLRRSPDDIPLMTPNTITMIPNTLTGMQPLHTFNTFSGGQNSTNLPHGHSTTRV

>Myocastor_coypus_this_paper

MSRPKGVLWVPLFCTPLGMMVNSNVLLWITALAIKFTLIDSQAQYPVVNTNYGKIRGLRTPLPNEILGPVEQYLGVPYASPPTGERRFQPPEPPSSWTGVRNATQFAGVCPQHLDERSLLHDMLPIWFTANLDTLMTYVQDQNEDCLYLNIYVPTEDGANTKKIADDITNNDRGDDEDIHDHNSKKPVMVYIHGGSYMEGTGNMIDGSILASYGNVIVITINYRLGILGFLSTGDQAAKGNYGLLDQIQALRWVEENVAAFGGDPKRVTIFGSGAGASCVSLLTLSHYSEGLFQKAIIQSGTALSSWAVNYQPAKYTRMLADKVGCNMLDTTDLVECLRAKNYKELIQQAITPATYHIAFGPVIDGDVIPDDPQILMEQGEFLNYDIMLGVNQGEGLKFVDGIVDNDDGVTPNDFDFSVSNFVDNLYGYPEGKDTLRETIKFMYTDWADKENPETRRKTLVALFTDHQWVAPAVATADLHAQYGSPTYFYAFYHHCQSEMKPSWADSAHGDEVPYVFGIPMIGPTELFSCNFSKNDVMLSAVVMTYWTNFAKTGDPNEPVPQDTKFIHTKPNRFEEVAWSKYNPKDQLYLHIGLKPRVRDHYRATKVAFWLELVPHLHNLNEIFQYVSTTTKVPPPDLTSYPYGTRRSPAKIWPTTKRPAITPAHSPKHAKDSHKTGPEDTTVLIETKRDYSTELSVTIAVGASLLFLNILAFAALYYKKDKRRHETHRRPSPQRSATNDIGHLQSEEMMSLQMKQLEHERDCESLQAHDTLRLTCPPDYALTLRRSPDDVPLMTPNTITMIPSTLTGMQPLHTFNTFSGGQNSSNLPHGHSTTRV

>Petromus_typicus_this_paper

MSRRRGLVWFPLFCRPVYVMVSSNALLWITALVVKFTLTDSQAQYPVVNTNYGKIRGLRTPLPNEILGPVEQYLGVPYASPPTGERRFQPPEPPSSWTGVRNATQFAAVCPQHLDERSLLHDMLPIWYTANLDTLMTYVQDQHEDCLYLNIYVPTEDGANTKKIADDITSNDRGDDEDIHDQNSKKPVMVYIHGGSYMEGTGNMIDGSILASYGNVIVITINYRLGILGFLSTGDQAAKGNYGLLDQIQALRWIEENVGAFGGDPKRVTIFGSGAGASCVSLLTLSHYSEGLFQKAIIQSGTALSSWAVNYQPAKYTRMLADKVGCNMLDTSDMVECLRGKSPKELIQQAITPATYHIAFGPVIDGDVIPDDPQILMEQGEFLNYDIMLGVNQGEGLKFVDGIVDNEDGVTPNDFDFSVSNFVDNLYGYPEGKDTLRETIKFMYTDWADKENPETRRKTLVALFTDHQWVAPAVATADLHAQYGSPTYFYAFYHHCQSEMKPSWADSAHGDEVPYVFGIPMIGPTELFSCNFSKNDVMLSAVVMTYWTNFAKTGDPNQPVPQDTKFIHTKPNRFEEVAWSKYNPKDQLYLHIGLKPRVRDHYRATKVAFWLELVPHLHNLNEIFQYVSTTTKVPPPEMTSFPYGTRRSPAKIWPTTKRPAITPANNPKHSKDSSKPGPEDTTVLIETKRDYSTELSVTIAVGASLLFLNILAFAALYYKKDKRRHETHRRPSPQRNAANDIAHIQSEEMMSLQMKQLEHDHECESLQAHDTLRLTCPPDYTLTLRRSPDDIPLMTPNTITMIPNTLTGMQPLHSFNTFSGGQNSTNLPHGHSTTRV

>Thryonomys_swinderianus_this_paper

MSRPRRLLWFPLFCTPVCVMVNSNVLLWITALVVKFTLIDSQAQYPVVNTNYGKIRGLRTPLPNEILGPVEQYLGVPYASPPTGERRFQPPEPPSSWTGVRNATQFAAVCPQHLDERSLLNDMLPVWFTANLDTLMTYVQDQNEDCLYLNIYVPTEDGANTKKIADDITSNDRGDDEDIHDPNGKKPVMVYIHGGSYMEGTGSMTDGSILASYGNVIVITVNYRLGVLGFLSTGDQAAKGNYGLLDQIQALRWIEENVGAFGGDPKRVTIFGSGAGASCVSLLTLSHYSEGLFQKAIICQDGQSGTALSSWAVNYQPAKYTRMLADKVGCNMLDTTDMVECLRGKNYKELIQQAITPATYHIAFGPVIDGDVIPDDPQILMEQGEFLNYDIMLGVNQGEGLKFVDGIVDNEDGVTPNDFDFSVSNFVDNLYGYPEGKDTLRETIKFMYTDWADKENPETRRKTLVALFTDHQWVAPAVATADLHAQYGSPTYFYAFYHHCQSEMKPSWADSAHGDEVPYVFGIPMIGPTELFSCNFSKNDVMLSAVVMTYWTNFAKTGDPNQPVPQDTKFIHTKPNRFEEVAWSKYNPKDQLYLHIGLKPRVRDHYRATKVAFWLELVPHLHNLNEIFQYVSTTTKVPPPDMTSFPYGTRRSPAKIWPTTKRPAITPANSPKHAKDAARPGPEDSTVLIETKRDYSTELSVTIAVGASLLFLNILAFAALYYKKDKRRHETHRRPSPQRSAPSDLAHMQNEELVSLQMKQLEQDHECESLQAHDALRLACPPDYTLTLRRSPDDMPLMTPNTITMIPNALTGMQPLHTFNTFSGAQNSSNLPHGHSTTRV
