# Supplementary material for: Of Humans and Gerbils— Independent Diversification of Neuroligin-4 Into X- and Y-Specific Genes in Primates and Rodents
Source: Front Mol Neurosci. 2022 Mar 30;15:838262. doi: 10.3389/fnmol.2022.838262 (PMC9005811; doi:10.3389/fnmol.2022.838262)
Supplement: Supplementary file 6 [file Data_Sheet_6.docx]

Supplementary Text File 3

The cDNA sequences below (FASTA format) served as the basis for the generation of the phylogenetic tree in Figure 5. All sequence references are additionally summarized in Suppl Data Sheet 2.

>X_Equus_caballus

ATGTCGAGACCCAAGAGACTGTTATGGCTTCCATTGTTCTGCACCCCGGTCTGCGTCATGTTGAACTCCAATGTTCTGCTGTGGATAACTGCTCTTGCCATTAAGTTCACGCTCATTGACAGCCAGGCACAGTATCCAGTTGTCAACACAAATTATGGCAAAATCCGGGGCCTAAGAACACCATTGCCCAATGAGATTTTGGGTCCAGTGGAGCAATACTTGGGAGTCCCTTATGCCTCGCCTCCCACTGGAGAGAGACGGTTTCAGCCCCCAGAACCCCCATCCTCGTGGACTGGGGTTCGAAATGCCACCCAGTTTGCTGCCGTGTGCCCCCAACACCTAGATGAGAGGTCTCTATTGCATGACATGCTGCCCATATGGTTTACTGCTAATTTGGATACTTTAATGACATATGTGCAAGATCAAAATGAAGACTGCCTTTACTTAAACATCTATGTGCCCACAGAGGATGGAGCCAACACAAAGAAAAACGCAGATGATATAACCAGTAATGACCGTGGTGAAGATGAAGACATCCATGATCAGAACAGTAAGAAGCCAGTTATGGTCTATATCCACGGCGGATCTTACATGGAGGGTACCGGCAATATGATCGACGGCAGCATTCTGGCAAGCTACGGGAACGTCATCGTGATAACAATTAACTACCGGCTGGGGATTTTAGGGTTTTTGAGTACCGGTGACCAGGCAGCTAAGGGAAACTATGGGCTTCTGGATCAAATCCAAGCGCTACGGTGGATCGAGGAGAACGTTGGCGCCTTTGGTGGGGATCCCAAGAGGGTGACCATCTTCGGCTCAGGCGCGGGAGCCTCTTGTGTCAGTCTCTTGACCTTGTCCCACTATTCAGAAGGTCTATTCCAGAAGGCGATCATCCAGAGCGGGACTGCCCTGTCCAGCTGGGCAGTGAACTACCAGCCTGCCAAGTACACTCGGATATTGGCAGATAAAGTCGGCTGCAACATGCTGGATACCACAGACATGGTGGAATGCCTTCGGAATAAGAACTATAAAGAGCTCATTCAGCAGACCATCACCCCAGCTACATACCACATTTCCTTTGGCCCTGTCATTGATGGGGATGTCATCCCAGACGACCCTCAGATCCTGATGGAGCAGGGGGAATTCCTTAATTATGACATCATGCTTGGTGTCAACCAGGGGGAAGGCTTGAAGTTTGTTGACGGCATTGTAGACAATGAAGATGGCGTGACGCCCAATGACTTTGACTTCTCTGTGTCCAACTTTGTGGACAACCTTTATGGCTATCCAGAGGGGAAAGACACCTTGCGGGAGACCATCAAATTCATGTACACAGACTGGGCAGATAAGGAAAACCCTGAAACTCGACGGAAAACTCTGGTAGCTCTCTTTACTGATCACCAGTGGGTGGCCCCCGCTGTGGCCACAGCTGACCTGCACGCTCAGTATGGGTCTCCAACATATTTCTATGCCTTCTACCACCACTGCCAAAGCGAAATGAAGCCCAGCTGGGCAGACTCAGCCCATGGCGACGAAGTACCCTATGTCTTTGGGATCCCCATGATTGGCCCTACTGAACTTTTCAGCTGTAACTTCTCTAAGAATGATGTTATGCTCAGTGCAGTGGTCATGACGTACTGGACAAATTTCGCCAAAACCGGTGACCCTAATCAGCCAGTTCCTCAGGATACCAAGTTCATTCACACAAAACCCAATCGCTTTGAGGAAGTGGCCTGGTCCAAGTATAACCCCAAAGACCAGCTATATCTGCATATTGGCCTGAAACCCAGAGTGAGGGATCACTATCGAGCTACAAAAGTGGCTTTCTGGTTGGAACTTGTCCCTCACTTGCACAACTTGAATGAGATATTCCAGTATGTTTCAACAACCACAAAGGTCCCTCCTCCTGACATGACCTCCTTTCCCTATGGAACCCGGCGATCTCCTGCCAAGATATGGCCAACAACCAAACGCCCAGCAATCACTCCTGCTAACAATCCCAAACACTCCAAGGACCCTCATAAAACAGGGCCTGAGGACACAACCGTCCTCATTGAAACCAAACGGGATTATTCCACTGAATTAAGTGTCACCATCGCCGTAGGGGCTTCCTTACTCTTCCTGAACATTTTGGCATTTGCTGCGCTGTACTACAAGAAGGACAAGAGACGCCATGAGACGCACAGGCGCCCCAGTCCTCAGAGAAATACAACGAATGACATTGCTCACATCCAGAATGAAGAGATCATGTCACTGCAGATGAAACAGCTGGAGCACGATCATGAGTGTGAATCCCTCCAAGCTCATGACACACTGAGGCTCACCTGTCCACCAGACTACACTCTCACGCTGCGCAGGTCCCCAGATGACATCCCACTTATGACACCAAACACCATCACCATGATTCCAAACACACTGACGGGGATGCAGCCTTTGCACACCTTCAATACCTTCAGTGGAGGACAAAACAGTACAAATTTACCCCACGGACATTCAACCACTAGAGTATAG

>Y_Equus_caballus

ATGCCAAGAGCCAAGGGACTGTTATGGTTTCCATTGCTCTGCACCCCAGTGTGCGTCATGTTGAACTCCAATGTTCTTCTGTGGATAACTGCTCTTGCCATTAAGTTCACACTCATTGACAGCCAGGCAAAGTATCCAGTCATCAACACAAATTATGGCAAAATCCGGGGCCTAAGAATACCATTGCCCAATGAGATTTTGGGTCCAGTTGAGCAATACTTGGGGGTCCCTTATGCCTCACCTCCCACTGGAGAGAGACGGTTTCAGCCTCCAGAACCCCCATCTTCATGGACTGGGGTTCGAAATGCCACCCAGTTTGCTGCTGTGTGCCCCCAAAACCTGGATGAGAGGTCTCTATTACATGATATGCTGCCCATATGGTTTACTGCTAATTTGGATACTTTAATGAATTATGTGCAAGATCAAAATGAAGATTGCCTTTACTTAAACATCTATGTGCCCACAGAGGATGACATCCATGATCAGAACAGTAAGAAGCCGGTTATGGTCTATATCCACGGCGGATCTTATATGGAGGGTACCGGCAATATGATTGACGGCAGCATTCTGGCAAGCTATGGGAACGTCATCGTGATAACTATTAACTACCGGCTGGGGATTTTAGGGTTTTTGAGTACTGGTGACCAGGCAGCTAAAGGCAACTATGGACTCCTGGATCAAATCCAAGCGCTACGGTGGATCGAGGAAAACGTTGGAGCCTTTGGTGGGGATCCCAAGAGGGTGACCATCTTTGGCTCTGGGGCGGGAGCCTCTTGTGTCAGTCTCTTGACCTTGTCCCACTATTCAGAAGGTCTGTTCCAGAAGGCAATCATCCAGAGTGGGACTGCCTTGTCCAGCTGGGCAGTGAACTATCAGCCTGCCAAGTACACTAGGATATTGGCAGATAAAGTTGGCTGCAACATGCTGGATACCACGGACATGGTAGAATGCCTTCAGAATAAGAACTACAAAGAGCTTATTCAGCAGACCATCACCCCAGCTACGTACCACATTTCCTTTGGTCCTGTTATCGATGGGGATGTCATTCCAGACGACCCTCAGATCCTGATGGAGCAGGGGGAATTCCTTAATTATGACATCATGCTTGGCATCAACCAGGGGGAAGGCTTGAAGTTTGTTGACGGCATTGTAGACAATGAAGATGGCGTGACGCCCAATGACTTTGACTTCTCTGTGTCCAACTTTGTGGACAACCTTTATGGTTATCCAGAGGGGAAAGACACCTTGAGGGAGACTATCAAATTCATGTACACAGACTGGGCAGATAAGGAAAATCCCGAAACTCGACGGAAAACTCTGGTAGCTCTCTTTACTGATCACCAGTGGGTGGCCCCTGCTGTGGCCACAGCTGACCTGCACGCTCAGTATGGGTCTCCAACATATTTCTATGCCTTCTACCACCACTGCCAAAGCGAAATGAAGCCCAGCTGGGCAGACTCAGCCCACGGTGATGAAGTACCCTATGTCTTTGGGATCCCCATGATTGGCCCTACTGAACTTTTCAGCTGTAACTTCTCTAAGAATGATGTCATGCTCAGTGCAGTGGTCATGACATACTGGACAAATTTTGCCAAAACTGGTGACCCTAATCAGCCAGTTCCTCAGGATACCAAGTTCATTCACACAAAACCCAATCGCTTTGAGGAAGTGGCCTGGTCCAAGTATAACCCCAAAGACCAGCTATATCTGCATATTGGCCTGAAACCGAGAGTGAGGGATCACTATCGAGCTACAAAAGTGGCTTTCTGGTTAGAACTTGTCCCTCACTTGCACAACTTGAATGAGATATTCCAATATGTTTCAACAACCACAAAGGTCCCTCCTCCTGACATGACCTCCTTTCCCTATGGAAACCGGAGATCTCCTGCCAAGATATGGCCAACAACCAAACGCCCAGCAATCACTCCTGCTAACAATCCCAAACACTCCAAGGACCCTCATAAAACAGGACCGGAAGACACAACTGTCCTCATCGAAACCAAAAGGGATTATTCCACTGAATTAAGTGTCACCATCGCCGTAGGGGCATCCTTACTCTTCCTCAACATTTTGGCATTTGCTGCGCTGTACTACAAGAAGGACAAGAGACGCCATGAGACTCACAGGCGCCCCAGTCCCCAGAGAAATACAACGAATGACCTTGCTCACATCCAGAATGAAGAGATCATGTCACTGCAGATGAAACAGCTGGAGCACGATCAAGAGTGTGAATCCATCCAAGCTCATGACACACTGAGGCTCACCTGTCCACCAGACTACACTCTCACGCTGCGCAGGTCCCCAGATGACATCCCACTTATGACACCAAACACCATCACCATGATTCCAAACACACTGACGGGGATGCAGCCTTTGAACGCTTTCAATACCTTCAGTGGAGGGCAAAACAGTACAAATTTACCCCATGGACATTCAACCACTAGAGTATAG

>a_Dipodomys_spectabilis_(this paper)

ATGGAGGGCCGCGTGGCCTGGTGGACCTGGACCCTGTGCCTGGTGGTGACCCCGTCCACCCTGGGGGGTCAGGGGTCGGAGGACGACCCCGTGGTGCGCACCCAGTACGGCCACCTGCGCGGCCTGAGGGCCTCGCTGCCCAGCGAGCTGCTGGGCCCCGTCCAGCAGTTCCTGGGGATCCCCTACGCCGCGCCCCCCGTGGGCCCACGGCGGTTTCTACCCCCCGAGCCCCCCACGGCCTGGCCGGGCATCCGTAACGCCACCCACTTCGCCCCCGTGTGTCCCCAACGCCTGGACGAACGGACGCTGCCCAGGGACATGCTGCCCAGCTGGCTCAGCGCCAACCTGGAGACCGTGGCCGGACTCCTGAGGGAGCAGAGCGAAGACTGTCTCTTCCTGAACGTCTACGTGCCCACCGAGGACGACATCCACGAGCCGGGCGCGCGCAGGCCGGTCATGGTGTACATCCACGGCGGCTCCTACATGGAGGGGACGGGGAACATGATCGACGGGAGCGTGCTGGCCAGCTACGGCAACGTCATCGTCATCACCCTCAACTACCGCCTGGGCATCCTGGGCTTCCTCAGCACCGGGGACCAGGCCGCGAAGGGCAACTACGGTCTCCTGGACCAGATCCAGGCGCTGCGCTGGGTGGAGGAGAACGTGGGCGCCTTCGGCGGGGACCCCAAGCGCGTCACCATCTTCGGCTCCGGGGCGGGCGCCTCGTGCGTCAGCCTGCTCACGCTCTCGCACTACTCGGAAGGACTGTTCCAGAAGGCCATCATCCAGAGCGGCACGGCGCTGTCCAGCTGGGCCGTCAACTACCAGCCGGCCCGGTACGCGCGCGCGCTGGCGGCGCAGCTGGGCTGCCCGTCCCCGGCGGACACCTCGGCGCTCGTGTCCTGCCTGCGCCTCAAATCCCCGCAGGAGCTCACCCGCCCGGTGGTCACCCCGGCCACCTACCACGTCGCCTTCGGCCCTGTGATCGACGGCGACGTGATCCCGGACGACCCGCAGATCCTGATGGAGCAGGGCGAGTTCCTCAACTACGACATCCTGCTGGGCGTGAACCAGGGCGAGGGCCTGGGCTTCGTGGACGGGCTGGTGGATGCGCTGGACGACGGAGTCAGCGCGGCGGCCTTCGAGGCCTCGATCGCCGCCTTCGTGGATCACCTCTACGGCTACCCCGAGGGCAAGCAGGCCCTGCGCGAGACCATCAAGTTCATGTACACGGACTGGGCCGACCGAGACAACCCCGAGACCCGCCGCAAGACCTTGGTGGCGCTGTTCACCGACCACCAGTGGGTGGCGCCCGCCGTGGCGACGGCCGACCTGCACGCGCAGTACGGCTCGCCCACCTACTTCTACGCCTTCTACCACCGCTGCCAGAGCGAGCTGAAGCCGGCCTGGGCGGACGCGGCGCACGGCGACGAGGTGCCCTACGTGTTCGGGGTGCCCATGGTGGGCCCCACGGAGCTGTTCAGCTGCAACTTCTCCAAGAACGACGTGATGCTGAGCGCCGTGGTGATGACGTACTGGACCAACTTCGCCAAGACGGGGGACCCCAACCAGCCCGTCCCCCAGGACACCAAGTTCATCCACACGAAGCCCAACCGTTTCGAGGAGGTGGCGTGGTCCAAGTACAACCCCAGGGACCAGCTCTACCTGCACATCGGCCTCAAGCCACGGGTTCGAGACCACTATCGAGCCACCAAGGTGGCCTTCTGGCTCGAACTCGTACCCCATCTGCACAACCTCAACGACATTCTGCAGTACGTCTCCACCACCACCACGCGCGCCCCTGATGTCACGTCCTCCTCTTCCTCCTCCCACCCCAGGCGAGCCACCAAGAGGCCCACTTCCTCCTCCTCGTCCATCCTGGGCCCCAAGGCCTTGCGCCCAGAAGGTAAACTGAGGCAGGGGGGGACCGAGCACAGCACCACCACGGTGCTGATCGAGACCAAGCGCGACTACTCCACCGAGCTCAGCGTCACCATCGCCGTGGGGGCCTCCCTCCTCTTCCTCAACATCCTGGCCTTCGCTGCCCTCTACTACAAGAAGGACAAGAGGCGACACCAGACCCACAGACGCCCCTCCAGCCCCCCCTCCACGTCCACTCGGCCCCCAGCGCCCCAGGACGCCGCCCAGCGCCACCTGCTGCGCGCCAGCGCGCCCACCGAACTCCTATCCGTGCAGCTGAACCCCAACGTCGGCGCCCACGAGGCCAGGGACCTCCAGGACGCGCTCCACCTGACCTGTCCCCCCGACTACGCGCTGACCCTGCGCAGATCCCCGGATGACATCCCCCTGATGACCCCCAGCACCATCACCACCCCGGGACCGACTTTGCACACGTTCAACACCTTTGGGGGGGCGGGTGGGGGAGGGGTGGGGGGGCCAGGAGGGGGGTCAGGGGGTCAGAACAACCCCCTCCCCCACGCCCACTCCACCACGCGCGTCTAG

>b_Dipodomys_spectabilis_this paper)

ATGGAGGGCCGCGTGGCCTGGTGGACCTGGACCCTGTGCCTGGTGGTGACCCCGTCCACCCTGGGGGGTCAGGGGTCGGAGGACGACCCCGTGGTGCGCACCCAGTACGGCCACCTGCGCGGCCTGAGGGCCTCGCTGCCCAGCGAGCTGCTGGGCCCCGTCCAGCAGTTCCTGGGGATCCCCTACGCCGCGCCCCCCGTGGGCCCACGGCGGTTTCTACCCCCCGAGCCCCCCACGGCCTGGCCGGGCATCCGTAACGCCACCCACTTCGCCCCCGTGTGTCCCCAACGCCTGGACGAACGGACGCTGCCCAGGGACATGCTGCCCAGCTGGCTCAGCGCCAACCTGGAGACCGTGGCCGGACTCCTGAGGGAGCAGAGCGAAGACTGTCTCTTCCTGAACGTCTACGTGCCCACCGAGGACGACATCCACGAGCCGGGCGCGCGCAGGCCGGTCATGGTGTACATCCACGGCGGCTCCTACATGGAGGGGACGGGGAACATGATCGACGGGAGCGTGCTGGCCAGCTACGGCAACGTCATCGTCATCACCCTCAACTACCGCCTGGGCATCCTGGGCTTCCTCAGCACCGGGGACCAGGCCGCGAAGGGCAACTACGGGCTCCTGGACCAGATCCAGGCGCTGCGCTGGGTGGAGGAGAACGTGGGCGCCTTCGGCGGGGACCCCAAGCGCGTCACCATCTTCGGCTCCGGGGCGGGCGCCTCGTGCGTCAGCCTGCTCACGCTCTCGCACTACTCGGAAGGCCTGTTCCAGAAGGCCATCATCCAGAGCGGCACGGCGCTGTCCAGCTGGGCCGTCAACTACCAGCCGGCGAGGTACGCTCGCGCGCTGGCGGCGCAGCTGGGCTGCCCGTCCCCGGCGGACACCTCGGCGCTCGTGTCCTGCCTGCGCCTCAAATCCCCGCAGGAGCTCACCCGCCCGGTGGTCACCCCGGCCACCTACCACGTCGCCTTCGGCCCTGTGATCGACGGCGACGTGATCCCGGACGACCCGCAGATCCTCATGGAGCAGGGCGAGTTCCTCAACTACGACATCCTGCTGGGCGTGAACCAGGGCGAGGGCCTGGGCTTCGTGGACGGACTCGTCGACGCGCTGGACGACGGAGTCAGCGCGGCGGCCTTCGAGGCCTCGATCGCCGCCTTCGTGGATCACCTGTACGGCTACCCCGAGGGCAAGCAGGCCCTGCGCGAGACCATCAAGTTCATGTACACGGACTGGGCCGACAGGGACAATCCCGAGACCCGCCGCAAGACCCTGGTGGCGCTGTTCACCGACCACCAGTGGGTGGCGCCCGCCGTGGCGACGGCCGACCTGCACGCGCAGTACGGCTCGCCCACCTACTTCTACGCCTTCTACCACCGCTGCCAGAGCGAGCTGAAGCCGGCCTGGGCGGACGCGGCGCACGGCGACGAGGTGCCCTACGTGTTCGGGGTGCCCATGGTGGGCCCCACGGAGCTGTTCAGCTGCAACTTCTCCAAGAACGACGTGATGCTGAGCGCCGTGGTGATGACGTACTGGACCAACTTCGCCAAGACGGGGGACCCCAACCAGCCCGTCCCCCAGGACACCAAGTTCATCCACACGAAGCCCAACCGTTTCGAGGAGGTGGCGTGGTCCAAGTACAACCCCAGGGACCAGCTCTACCTGCACATCGGCCTCAAGCCACGGGTTCGAGACCACTATCGAGCCACCAAGGTGGCCTTCTGGCTCGAACTCGTACCCCATCTGCACAACCTCAACGACATTCTGCAGTACGTCTCCACCACCACCACGCGCGCCCCTGATGTCACGTCCTCCTCTTCCTCCTCCCACCCCAGGCGAGCCACCAAGAGGCCCACTTCCTCCTCCTCGTCCATCCTGGGCCCCAAGGCCTTGCGCCCAGAAGGTAAACTGAGGCAGGGGGGGACTGAGCACAGCACCACCACGGTGCTGATCGAGACCAAGCGCGACTACTCCACCGAGCTCAGCGTCACCATCGCCGTGGGGGCCTCCCTCCTCTTCCTCAACATCCTGGCCTTCGCTGCCCTCTACTACAAGAAGGACAAGAGGCGACACCAGACCCACAGACGCCCCTCCAGCCCCCCCTCCACGTCCACTCGGACCCCAGCGCCCCAGGACGCCGCCCAGCGCCACCTGCTGCGCGCCAGCGCGCCCACCGAACTCCTATCCGTGCAGCTGAACCCCAACGTCGGCGCCCACGAGGCCAGGGACCTCCAGGACGCGCTCCACCTGACCTGTCCCCCCGACTACGCGCTGACCCTGCGCAGATCCCCGGATGACATCCCCCTGATGACCCCCAGCACCATCACCACCCCGGGACCGACTTTGCACACGTTCAACACCTTTGGGGGGGCGGGTGGGGGAGGGGTGGGGGGGCCAGGAGGGGGGTCAGGGGGTCAGAACAACCCCCTCCCCCACGCCCACTCCACCACGCGCGTCTAG

>X_Callithrix_jacchus

ATGTCGCGGCCCCAGGGACTGCTATGGCTTCCTTTGTTATTCACCCCGGTCTGCGTCGTGTTAAACTCCAATGTCCTCCTGTGGATAACTGCTCTTGCCATCAAGTTCACTCTCATCGACAGCCAAGCACAGTATCCAGTTGTGAACACAAACTATGGCAAAGTACGAGGCCTAAGAACACCGTTACCCAATGAGATCTTGGGTCCAGTGGAGCAGTACTTAGGGGTCCCCTATGCCTCACCCCCCACTGGAGAGAGGAGGTTTCAGCCCCCAGAGCCCCCATCCTCCTGGACTGGCATCCGAAATGCAACCCAATTTGCTGCTGTGTGCCCCCAGCACCTGGATGAGAGATCTTTACTGCATGACATGCTGCCCATCTGGTTTACGGCCAACTTGGATACACTGATGACCTATGTTCAAGATCAAAATGAAGACTGCCTTTACTTAAACATCTATGTGCCCACAGAAGATGGAGCCAACACAAAGAAAAACGCAGATGATATAACCAGTAATGACCGTGGTGAAGATGAAGATATTCATGATCAGAACAGTAAGAAGCCGGTTATGGTCTATATCCATGGGGGATCTTACATGGAGGGCACCGGCAACATGATTGACGGCAGCATTTTGGCAAGCTATGGGAACGTCATTGTGATCACCATTAACTACCGCCTGGGAATACTAGGGTTTTTAAGTACCGGTGACCAAGCAGCAAAAGGCAACTATGGGCTCCTGGATCAGATTCAAGCACTGCGCTGGATTGAGGAGAATGTGGGAGCCTTCGGTGGGGACCCCAAGCGCGTGACCATCTTTGGCTCGGGGGCTGGGGCCTCCTGTGTCAGCCTGTTGACCCTATCCCACTACTCAGAAGGTCTCTTCCAGAAGGCCATCATTCAGAGTGGCACTGCCCTGTCCAGCTGGGCAGTGAACTACCAGCCGGCCAAGTACACTCGGATATTGGCAGACAAGGTCGGCTGCAACATGCTGGACACCACGGACATGGTAGAATGCCTTCGGAACAAGAACTACAAGGAGCTCATCCAGCAGACCATCACCCCAGCCACCTACCACATAGCCTTTGGGCCCGTGATTGACGGCGACGTGATCCCAGACGACCCCCAGATCCTGATGGAGCAAGGCGAGTTCCTCAACTACGACATCATGCTGGGTGTCAACCAAGGAGAAGGCTTGAAGTTCGTGGACGGCATCGTGGACAATGAGGACGGTGTGACGCCCAATGACTTTGACTTCTCCGTGTCCAACTTCGTGGACAACCTTTACGGCTACCCTGAAGGGAAAGACACTTTGCGGGAGACTATCAAGTTCATGTACACAGACTGGGCCGATAAGGAAAACCCGGAGACGCGGCGGAAGACTCTGGTGGCTCTCTTTACTGACCACCAGTGGGTGGCCCCCGCTGTGGCCACCGCCGACCTGCACGCGCAGTATGGCTCCCCGACCTATTTCTATGCCTTCTATCATCACTGCCAAAGCGAAATGAAGCCCAGCTGGGCAGACTCGGCCCATGGCGACGAGGTCCCCTATGTCTTCGGCGTCCCCATGATCGGCCCAACCGAGCTCTTCAGTTGTAACTTCTCTAAGAACGACGTCATGCTCAGCGCCGTGGTCATGACCTACTGGACGAACTTCGCCAAAACTGGTGATCCAAATCAACCAGTTCCACAGGATACCAAGTTCATTCACACGAAACCCAACCGCTTTGAGGAAGTGGCCTGGTCCAAATATAATCCCAAAGACCAGCTCTATCTGCATATTGGGTTGAAACCCAGAGTGAGAGATCATTACCGGGCAACGAAAGTGGCTTTCTGGTTGGAACTCGTTCCTCATTTGCACAACTTGAACGAGATATTCCAGTATGTTTCAACAACCACAAAGGTCCCTCCTCCAGACATGACCTCATTTCCCTATGGTACCCGGCGATCTCCCGCCAAGATATGGCCGACCACCAAACGCCCAGCTATCACTCCTGCCAACAATCCCAAGCACTCTAAGGACCCTCACAAAACGGGGCCCGAGGACACAACTGTCCTCATTGAAACCAAACGGGATTATTCCACCGAATTAAGCGTCACCATTGCCGTCGGGGCGTCGCTCCTCTTCCTCAACATTTTGGCCTTCGCAGCGCTGTACTACAAAAAGGACAAGAGGCGCCATGAGACTCACAGGCGCCCCAGTCCCCAGAGAAACACCACAAATGATATCGCTCACATCCAGAATGAAGAGATGATGTCTCTGCAGATGAAGCAGCTGGAGCATGATCACGAGTGTGAGTCGCTGCAGGCGCACGACACACTGAGGCTCACCTGCCCACCAGACTACACCCTCACGCTGCGCCGGTCGCCAGATGACATCCCGCTGATGACGCCAAACACCATCACCATGATTCCAAACACACTGACCGGGATGCAGCCTTTGCACACCTTCAACACCTTCAGCGGAGGGCAGAACAGTACCAACTTACCCCACGGACATTCCACCACTAGAGTATAG

>Y_Callithrix_jacchus

ATGTCACGGCCCAAGATACTGCTATGGCTTCCTTTGCTATTTACCCCGGTCTGCATCATGTTAAAGTCCAATGTCCTCCTGTGGATGACTGCTCTTGCCATCAAGTTCACTCTCATTGACAGCCAAGCACAGTATCCAGTTGTCAACACAAATTATGGCAAAGTACGAGGCCTAAGAACACCAATACCCAATGAGATCTTGGGTCCAGTGGAGCAGTACTTAGGGGTTCCCTATGCCTCACCCCCAACTGGAGAGAGGCGATTTCAATCCCCTGAGCCCCCCTCCTCCTGGACTGGCATCCGAAATGCGACTCAGTTTGCTGCTGTATGTCCCCAGCACCTGGATGAGAGATCTTTACTGCATGACATGCTGCCCATCTGGTTTACCGCCAATTTGGATACATTGATGACCTATTCTCAAGATCAAAATGAAGACTGCCTTTACTTAAACATCTATGTGCCCACAGAAAATGGAGACAACAGAAAGAAAAACGCAGATGATATAACCGGTAATGACCATGGGGAAGATGAAGATATTCATGATCAGAACAGTAAGAAGCCGGTTATGGTCTATATCCATGGGGGATCTTACATGGAGGGCACGGCCAACATAATTGATGGCAGCATTTTGGCAAGCTATGGGAACGTCATTGTGATCACCATTAACTACCGCCTGGGAATACTAGGGTTTTTAAGTACCGGTGACCAGGCAGCAAAAGGCAACTATGGGCTCCTGGACCAAATTCAAGCACTACGGTGGATTGAGGAGAATGTGGGAGCCTTTGGCGGGGACCCCAAGCGCGTGACCATCTTTGGCTCAGGGGCTGGGGCTTCCTGTGTCAGCCTATTGACCCTATCCCACTACTCAGAAGGTCTCTTCCAGAAGGCCATTGTTCAGAGCGGCACCGCCCTGTCCAGCTGGGCAGTGAACTACCAGCCAGCCAAGTACACTCGGATTTTGGCAGACAAGGTCGGCTGCAACATGCTGGACACCATGGACATGGTAGAATGCCTGCGGAACAAGAACTACAAGGAGCTCATCCAGCAGACCATCACCCCAGCCACCTACCACATAGCCTTTGGGCCCGTGATTGACGGCGACGTGATCCCAGACGACCCCCAGATCCTGATGGAACAAGGCGAGTTCCTCAATTATGACATCATGCTGGGCGTCAACCAAGGAGAAGGCTTGAAGTTCGTGGATGGCATCGTGGACAATGAGGACGGTGTGACACCCAATGACTTTGATTTCTCCGTGTCCAACTTTGTGGACAACCTTTACGGCTACCCTGAAGGGAAAGACACTTTGCGGGAGACTATCAAGTTTATGTACACAGACTGGGCCGATAAGGAAAACCCTGAGACGCGGCGGAAGACTCTGGTGGCTCTCTTTACTGACCACCAGTGGGTGGCTCCCGCCGTGGCCACCGCTGACCTGCACGCGCAATATGGCTCCCCCACCTATTTCTATGCCTTCTATCATCACTGTCAAAGCGAAATGAAGCCCAGCTGGGCAGACTCTGCCCATGGCGACGAGGTCCCCTATGTCTTTGGCATCCCCATGATCGGCCCAACTGAGCTCTTCAGTTGTAACTTCTCCAAGAACGACGTCATGCTCAGTGCCCTGGTCATGACCTACTGGACAAACTTCGCCAAAACTGGTGATCCAAATCAACCAGTTCCACAGGATACCAAGTTCATTCACATGAAACCCAACCGCTTTGAGGAAGTGGCCTGGTCCAAGTATAATCCCAAAGACCAGCTCTATCTACATATTGGGTTGAAACCAAGAGTGAGAGATCATTACCGGGCAACAAAAGTGGCTTTCTGGTTGGAACTCGTTCCTCATTTGCACAACTTGAACGAGATATTCCAGTATGTTTCAACAACCACAAAGGTCCCTCCTCCATACATGACCTCATTTCCCTATGGTACCTGGCGATCTCCCACCAAGATATGGCCGACCACCAAACGCCCAGCTATCACTCCTGCCAACAATCCCAAACACTCGAAGGACCCTCACAAAACGGGTCCCGAGGACACAACTGTCCTCATTGAAACCAAACGGGATTATTCCACCGAATTAAGCATCACCATTGCCGTCGGGGCGTCGCTCCTCTTCCTCAACATTTTGGCCTTTGCAGCACTGTACTACAAAAAGGACAAGAGGCGCCATGAGACTCACAGGCGCCCCAGTCCCCAGAGAAACACCACAAATAATATCGCTCACATCCAGAATGAAGAGATGATGTCTCTGCAGATGAGGCAGCTGGAGCACGATCACGAGTGTGCGTCGCTCCAGGCACACGACACACTGAGGCTCACCTGCCCACCAGACTACACCCTCACTCTGCACCAGTCGTCAGATGACATCCTGCTGATGACGCCAAACACCATCACCATGATTCCAAACACACTGACAAGGATGCAGCCTTTGCACACCTTCAACACCTTCAGCGGAGGGCAGAACAGTACCAACTTACCCCATGGACATTCCACCACTAGAGTATAG

>X_Homo_sapiens

ATGTCACGGCCCCAGGGACTGCTATGGCTTCCTTTGTTGTTCACCCCGGTCTGCGTCATGTTAAACTCCAATGTCCTCCTGTGGTTAACTGCTCTTGCCATCAAGTTCACCCTCATTGACAGCCAAGCACAGTATCCAGTTGTCAACACAAATTATGGCAAAATCCGGGGCCTAAGAACACCGTTACCCAATGAGATCTTGGGTCCAGTGGAGCAGTACTTAGGGGTCCCCTATGCCTCACCCCCCACTGGAGAGAGGCGGTTTCAGCCCCCAGAACCCCCGTCCTCCTGGACTGGCATCCGAAATACTACTCAGTTTGCTGCTGTGTGCCCCCAGCACCTGGATGAGAGATCCTTACTGCATGACATGCTGCCCATCTGGTTTACCGCCAATTTGGATACTTTGATGACCTATGTTCAAGATCAAAATGAAGACTGCCTTTACTTAAACATCTACGTGCCCACGGAAGATGGAGCCAACACAAAGAAAAACGCAGATGATATAACGAGTAATGACCGTGGTGAAGACGAAGATATTCATGATCAGAACAGTAAGAAGCCCGTCATGGTCTATATCCATGGGGGATCTTACATGGAGGGCACCGGCAACATGATTGACGGCAGCATTTTGGCAAGCTACGGAAACGTCATCGTGATCACCATTAACTACCGTCTGGGAATACTAGGGTTTTTAAGTACCGGTGACCAGGCAGCAAAAGGCAACTATGGGCTCCTGGATCAGATTCAAGCACTGCGGTGGATTGAGGAGAATGTGGGAGCCTTTGGCGGGGACCCCAAGAGAGTGACCATCTTTGGCTCGGGGGCTGGGGCCTCCTGTGTCAGCCTGTTGACCCTGTCCCACTACTCAGAAGGTCTCTTCCAGAAGGCCATCATTCAGAGCGGCACCGCCCTGTCCAGCTGGGCAGTGAACTACCAGCCGGCCAAGTACACTCGGATATTGGCAGACAAGGTCGGCTGCAACATGCTGGACACCACGGACATGGTAGAATGCCTGCGGAACAAGAACTACAAGGAGCTCATCCAGCAGACCATCACCCCGGCCACCTACCACATAGCCTTCGGGCCGGTGATCGACGGCGACGTCATCCCAGACGACCCCCAGATCCTGATGGAGCAAGGCGAGTTCCTCAACTACGACATCATGCTGGGCGTCAACCAAGGGGAAGGCCTGAAGTTCGTGGACGGCATCGTGGATAACGAGGACGGTGTGACGCCCAACGACTTTGACTTCTCCGTGTCCAACTTCGTGGACAACCTTTACGGCTACCCTGAAGGGAAAGACACTTTGCGGGAGACTATCAAGTTCATGTACACAGACTGGGCCGATAAGGAAAACCCGGAGACGCGGCGGAAAACCCTGGTGGCTCTCTTTACTGACCACCAGTGGGTGGCCCCCGCCGTGGCCACCGCCGACCTGCACGCGCAGTACGGCTCCCCCACCTACTTCTATGCCTTCTATCATCACTGCCAAAGCGAAATGAAGCCCAGCTGGGCAGATTCGGCCCATGGTGATGAGGTCCCCTATGTCTTCGGCATCCCCATGATCGGTCCCACCGAGCTCTTCAGTTGTAACTTTTCCAAGAACGACGTCATGCTCAGCGCCGTGGTCATGACCTACTGGACGAACTTCGCCAAAACTGGTGATCCAAATCAACCAGTTCCTCAGGATACCAAGTTCATTCACACAAAACCCAACCGCTTTGAAGAAGTGGCCTGGTCCAAGTATAATCCCAAAGACCAGCTCTATCTGCATATTGGCTTGAAACCCAGAGTGAGAGATCACTACCGGGCAACGAAAGTGGCTTTCTGGTTGGAACTCGTTCCTCATTTGCACAACTTGAACGAGATATTCCAGTATGTTTCAACAACCACAAAGGTTCCTCCACCAGACATGACATCATTTCCCTATGGCACCCGGCGATCTCCCGCCAAGATATGGCCAACCACCAAACGCCCAGCAATCACTCCTGCCAACAATCCCAAACACTCTAAGGACCCTCACAAAACAGGGCCTGAGGACACAACTGTCCTCATTGAAACCAAACGAGATTATTCCACCGAATTAAGTGTCACCATTGCCGTCGGGGCGTCGCTCCTCTTCCTCAACATCTTAGCTTTTGCGGCGCTGTACTACAAAAAGGACAAGAGGCGCCATGAGACTCACAGGCGCCCCAGTCCCCAGAGAAACACCACAAATGATATCGCTCACATCCAGAACGAAGAGATCATGTCTCTGCAGATGAAGCAGCTGGAACACGATCACGAGTGTGAGTCGCTGCAGGCACACGACACACTGAGGCTCACCTGCCCGCCAGACTACACCCTCACGCTGCGCCGGTCGCCAGATGACATCCCACTTATGACGCCAAACACCATCACCATGATTCCAAACACACTGACGGGGATGCAGCCTTTGCACACTTTTAACACCTTCAGTGGAGGACAAAACAGTACAAATTTACCCCACGGACATTCCACCACTAGAGTATAG

>Y_Homo_sapiens

ATGTTGCGTCCCCAGGGACTGCTATGGCTCCCTTTGTTGTTCACCTCTGTCTGTGTCATGTTAAACTCCAATGTTCTTCTGTGGATAACTGCTCTTGCCATCAAGTTCACCCTCATTGACAGCCAAGCACAGTATCCAGTTGTCAACACAAATTATGGTAAAATCCAGGGCCTAAGAACACCATTACCCAGTGAGATCTTGGGTCCAGTGGAGCAGTACTTAGGGGTCCCCTATGCCTCACCCCCAACTGGAGAGAGGCGGTTTCAGCCACCAGAATCCCCATCCTCCTGGACTGGCATCCGAAATGCTACTCAGTTTTCTGCTGTGTGCCCCCAGCACCTGGATGAAAGATTCTTATTGCATGACATGCTGCCCATCTGGTTTACCACCAGTTTGGATACTTTGATGACCTATGTTCAAGATCAAAATGAAGACTGCCTTTACTTAAACATCTATGTGCCCATGGAAGATGGAACCAACATAAAGAGAAATGCAGACGATATAACCAGTAATGACCATGGTGAAGATAAAGATATTCATGAACAGAACAGTAAGAAGCCTGTTATGGTCTATATCCATGGGGGATCTTACATGGAGGGAACCGGTAACATGATTGATGGCAGCATTTTGGCCAGCTATGGGAACGTCATCGTTATCACCATTAACTACCGTCTGGGAATACTAGGGTTTTTAAGTACCGGTGACCAGGCAGCAAAAGGCAACTATGGGCTCCTGGATCAGATTCAAGCACTGAGGTGGATTGAGGAGAATGTCGGAGCCTTTGGCGGGGACCCCAAGAGAGTGACTATCTTTGGCTCGGGGGCTGGGGCCTCCTGTGTCAGCCTGTTGACCCTGTCCCACTACTCAGAAGGTCTCTTCCAGAAGGCCATCATTCAGAGCGGCACTGCCCTGTCCAGCTGGGCAGTGAACTACCAGCCGGCCAAGTACACTCGGATATTGGCAGACAAGGTCGGCTGCAACATGCTGGACACCACGGACATGGTAGAATGTCTGAAGAACAAGAACTACAAGGAGCTCATCCAGCAGACCATCACCCCGGCCACCTACCACATAGCCTTTGGGCCGGTGATCGACGGCGACGTCATCCCAGACGACCCCCAGATCCTGATGGAGCAAGGCGAGTTCCTCAACTACGACATCATGCTGGGCGTCAACCAAGGGGAAGGCCTGAAGTTCGTGGACGGCATCGTGGATAACGAGGACGGTGTGACGCCCAACGACTTTGACTTCTCCGTGTCCAACTTCGTGGACAACCTTTACGGCTACCCTGAAGGGAAAGACACTTTGCGGGAGACTATCAAGTTCATGTACACAGACTGGGCCGATAAGGAAAACCCGGAGACGCGGCGGAAAACCCTGGTGGCTCTCTTTACTGACCATCAGTGGGTGGCCCCCGCCGTGGCCACCGCCGACCTGCACGCGCAGTACGGCTCCCCCACCTACTTCTATGCCTTCTATCATCACTGCCAAAGCGAAATGAAGCCCAGCTGGGCAGATTCGGCCCATGGCGATGAAGTCCCCTATGTCTTCGGCATCCCCATGATCGGTCCCACAGAGCTCTTCAGTTGTAATTTCTCCAAGAACGACGTCATGCTCAGTGCCGTGGTGATGACCTACTGGACGAACTTCGCCAAAACTGGTGATCCAAACCAACCAGTTCCTCAGGATACCAAGTTCATTCATACAAAACCCAATCGCTTTGAAGAAGTGGCCTGGTCCAAGTATAATCCCAAAGACCAGCTCTATCTGCATATTGGCTTGAAACCCAGAGTGAGAGATCACTACCGGGCAACGAAAGTGGCTTTCTGGTTGGAATTGGTTCCTCATTTGCACAACTTGAACGAGATATTCCAGTATGTTTCAACAACCACAAAGGTTCCTCCACCAGACATGACATCATTTCCCTATGGCACCCGGCGATCTCCCGCCAAGATATGGCCAACCACCAAACGCCCAGCAATCACTCCTGCCAACAATCCCAAACACTCTAAGGACCCTCACAAAACAGGGCCCGAGGACACAACTGTCCTCATTGAAACCAAACGAGATTATTCCACCGAATTAAGTGTCACCATTGCCGTCGGGGCGTCGCTCCTCTTCCTCAACATCTTAGCCTTTGCGGCGCTGTACTACAAAAAGGACAAGAGGCGCCATGAGACTCACAGGCACCCCAGTCCCCAGAGAAACACCACAAATGATATCACTCACATCCAGAACGAAGAGATCATGTCTCTGCAGATGAAGCAGCTGGAACACGATCACGAGTGTGAGTCGCTGCAGGCACACGACACGCTGAGGCTCACCTGCCCTCCAGACTACACCCTCACGCTGCGCCGGTCGCCGGATGACATCCCATTTATGACGCCAAACACCATCACCATGATTCCAAACACATTGATGGGGATGCAGCCTTTACACACTTTTAAAACCTTCAGTGGAGGACAAAACAGTACAAATTTACCCCACGGACATTCCACCACTAGAGTATAG

>Y_Pan_troglodytes

ATGTTGCGTCCCCAGGGACTGCTATGGCTCCCTTTGTTATTCACCTCTGTCTGTGTCATGTTAAACTCCAATGTTCTTCTGTGGATAACTGCTCTTGCCATCAAGTTCACCCTCATTGACAGCCAAGCACAGTATCCAGTTGTCAACACAAATTATGGTAAAATCCAGGGCCTAAGAACACCATTACCCAGTGAGATCTTGGGTCCAGTGGAGCAGTACTTAGGGGTCCCCTATGCCTCACCCCCAACTGGAGAGAGGCGGTTTCAGCCCCCAGAATCCCCATCCTCCTGGACTGGCATCCGAAATGCTACTCAGTTTGCTGCTGTGTGCCCCCAGCACCTGGATGAAAGATTCTTATTGCATGACATGCTGCCCATCTGGTTTACCGCCAATTTGGATACTTTGATGACCTATGTTCAAGATCAAAATGAAGACTGCCTTTACTTAAACATCTATGTGCCCACGGAAGATGGAACCAACATAAAGAGAAACGCAGACGATATAACCAGTAATGACCATGGTGAAGATGAAGATATTCATGAACAGAACAGTAAGAAGCCAGTTATGGTCTATATCCATGGGGGATCCTACATGGAGGGAACCGGTAACATGATTGATGGCAGCATTTTGGCCAGCTATGGGAACGTCATCGTTATCACCATTAACTACCGTCTGGGAATACTAGGGTTTTTAAGTACCGGTGACCAGGCAGCAAAAGGCAACTATGGGCTCCTGGATCAGATTCAAGCACTGCGGTGGATTGAGGAGAATGTCGGAGCCTTTGGCGGGGACCCCAAGAGAGTGACTATCTTTGGCTCAGGGGCTGGGGCCTCCTGTGTCAGCCTGTTGACCCTGTCCCACTACTCAGAAGGTCTCTTCCAGAAGGCCATCATTCAGAGCGGCACCGCCCTGTCCAGCTGGGCAGTGAACTACCAGCCGGCCAAGTACACTCGGATATTGGCAGACAAGGTCGGCTGCAACATGCTGGACACTACGGACATGGTAGAATGTCTGAAGAACAAGAACTACAAGGAGCTCATCCAGCAGACCATCACCCCGGCCACCTACCACATAGCCTTCGGGCCGGTGATCGACGGTGACGTCATCCCAGACGACCCCCAGATCCTGATGGAGCAAGGCGAGTTCCTCAATTACGACATCATGCTGGGTGTCAACCAAGGGGAAGGCCTGAAGTTCGTGGACGGCATCGTGGATAACGAGGACGGTGTGACGCCCAACGACTTTGACTTCTCCGTGTCCAACTTCGTGGACAACCTTTACGGCTACCCTGAAGGGAAAGACATTTTGCGGGAGACTATCAAGTTCATGTACACAGACTGGGCCGATAAGGAAAACCCGGAGACGCGGCGGAAAACCCTGGTGGCTCTCTTTACTGACCATCAGTGGGGGGCCCCCGCCGTGGCCACCGCCGACCTGCACGCGCAGTACGGCTCCCCCACCTACTTCTATGCCTTCTATCATCACTGCCAAAGCGAAATGAAGCCCAGCTGGGCAGATTCGGCCCATGGCGATGAAGTCCCCTATGTCTTCGGCATCCCCATGATCGGTCCCACCGAGCTCTTCCGTTGTAATTTCTCCAAGAACGACGTCATGCTCAGCGCCGTGGTCATGACCTACTGGACGAACTTCGCCAAAACTGGTGATCCAAACCAACCAGTTCCTCAGGATACCAAGTTCATTCATACAAAACCCAATCGCTTTCAAGAAGTGGCCTGGTCCAAGTATAATCCCAAAGACCAACTCTATCTGCATATTGGGTTGAAACCCAGAGTGAGAGATCACTACCGGGCAACGAAAGTGGCTTTCTGGTTGGAACTCGTTCCTCATTTGCACAACTTGAACGAGATATTCCAGTATGTTTCAACAACCACAAAGGTTCCTCCACCAGACATGACATCATTTCCCTATGGCACCCGGCGATCTCCCGCCAAGATATGGCCAACCACCAAACGCCCAGCAATCACTCCTGCCAACAATCCCAAACACTCTAAGGACCCTCACAAAACAGGGCCCGAGGACACAACTGTCCTCATTGAAACCAAACGAGATTATTCCACCGACTTAAGTGTCACCATTGCCGTCGGGGCGTCGCTCCTCTTCCTCAACATCTTAGCCTTTGCGGCGCTGTACTACAAAAAGGACAAGAGGCGCCATGAGACTCACAGGCGCCCCAGTCCCCAGAGAAACACCACAAATGATATCCCTCACATCCAGAACGAAGAGATCATGTCTCTGCAGATGAAGCAGCTGGAACACGATCACGAGTGTGAGTCGCTGCAGGCACACGACGCGCTGAGGCTCACCTGCCCTCCAGACTACACCCTCACGCTGCGCCGGTCGCCAGATGACATCCCACTTATGACGCCAAACACCATCACCATGATTCCAAACACACTGACGGGGATGCAGCCTTTGCACACTTTTAACACCTTCAGTGGAGGACAAAACAGTACAAATTTACCCCACGGACATTCCACCACTAGAGTATAG

>X_Pan_troglodytes

ATGTCACGGCCCCAGGGACTGCTATGGCTTCCTTTGTTGTTCACCCCGGTCTGCGTCATGTTAAACTCCAATGTCCTCCTGTGGTTAACTGCTCTTGCCATCAAGTTCACCCTCATTGACAGCCAAGCACAGTATCCAGTTGTCAACACAAATTATGGCAAAATCCGGGGCCTAAGAACACCGTTACCCAATGAGATCTTGGGTCCAGTGGAGCAGTACTTAGGGGTCCCCTATGCCTCACCCCCAACTGGAGAGAGGCGGTTTCAGCCCCCAGAACCCCCGTCCTCCTGGACTGGCATCCGAAATACTACTCAGTTTGCTGCTGTGTGCCCCCAGCACCTGGATGAGAGATCCTTACTGCATGACATGCTGCCCATCTGGTTTACCGCCAATTTGGATACTTTGATGACCTATGTTCAAGATCAAAATGAAGACTGCCTTTACTTAAACATCTACGTGCCCACGGAAGATGGAGCCAACACAAAGAAAAACGCAGATGATATAACGAGTAATGACCGTGGTGAAGACGAAGATATTCATGATCAGAACAGTAAGAAGCCCGTCATGGTCTATATCCATGGGGGATCTTACATGGAGGGCACCGGCAACATGATTGACGGCAGCATTTTGGCCAGCTACGGAAACGTCATCGTGATCACCATTAACTACCGTCTGGGAATACTAGGGTTTTTAAGTACCGGTGACCAGGCAGCAAAAGGCAACTATGGGCTCCTGGATCAGATTCAAGCACTGCGGTGGATTGAGGAGAATGTGGGAGCCTTTGGCGGGGACCCCAAGAGAGTGACCATCTTTGGCTCCGGGGCTGGGGCCTCCTGTGTCAGCCTGTTGACCCTGTCCCACTACTCAGAAGGTCTCTTCCAGAAGGCCATCATTCAGAGCGGCACCGCCCTGTCCAGCTGGGCAGTGAACTACCAGCCGGCCAAGTACACTCGGATATTGGCAGACAAGGTCGGCTGCAACATGCTGGACACTACGGACATGGTAGAATGCCTGCGGAACAAGAACTACAAGGAGCTCATCCAGCAGACCATCACCCCGGCCACCTACCACATAGCCTTCGGGCCGGTGATCGACGGTGACGTCATCCCAGACGACCCCCAGATCCTGATGGAGCAAGGCGAGTTCCTCAACTATGACATCATGCTGGGCGTCAACCAAGGGGAAGGCCTGAAGTTCGTGGACGGCATCGTGGATAACGAGGACGGTGTGACGCCCAACGACTTTGACTTCTCCGTGTCCAACTTCGTGGACAACCTTTACGGCTACCCTGAAGGGAAAGACACTTTGCGCGAGACTATCAAGTTCATGTACACAGACTGGGCCGATAAGGAAAACCCGGAGACGCGGCGGAAAACCCTGGTGGCTCTCTTTACTGACCACCAGTGGGTGGCCCCCGCCGTGGCCACTGCCGACCTGCACGCGCAGTACGGCTCCCCCACCTACTTCTATGCCTTCTATCATCACTGCCAAAGCGAAATGAAGCCCAGCTGGGCAGATTCGGCCCATGGTGATGAGGTCCCCTATGTCTTCGGCATCCCCATGATCGGTCCCACCGAGCTCTTCAGTTGTAACTTTTCCAAGAACGACGTCATGCTCAGCGCCGTGGTCATGACCTACTGGACGAACTTCGCCAAAACTGGTGATCCAAATCAACCAGTTCCTCAGGATACCAAGTTCATTCACACAAAACCCAACCGCTTTGAAGAAGTGGCCTGGTCCAAGTATAATCCCAAAGACCAGCTCTATCTGCATATTGGCTTGAAACCCAGAGTGAGAGATCACTACCGGGCAACGAAAGTGGCTTTCTGGTTGGAACTCGTTCCTCATTTGCACAACTTGAACGAGATATTCCAGTATGTTTCAACAACCACAAAGGTTCCTCCACCAGACATGACATCATTTCCCTATGGCACCCGGCGATCTCCCGCCAAGATATGGCCAACCACCAAACGCCCAGCAATCACTCCTGCCAACAATCCCAAACACTCTAAGGACCCTCACAAAACAGGGCCCGAGGATACAACTGTCCTCATTGAAACCAAACGAGATTATTCCACCGAATTAAGTGTCACCATTGCCGTCGGGGCGTCGCTCCTCTTCCTCAACATCTTAGCCTTTGCGGCGCTGTACTACAAAAAGGACAAGAGGCGCCATGAGACTCACAGGCGCCCCAGTCCCCAGAGAAACACCACAAATGATATCGCTCACATCCAGAACGAAGAGATCATGTCTCTGCAGATGAAGCAGCTGGAACACGATCACGAGTGTGAGTCGCTGCAGGCACACGACACGCTGAGGCTTACCTGCCCGCCAGACTACACCCTCACGCTGCGCCGGTCGCCAGATGACATCCCACTTATGACGCCAAACACCATCACCATGATTCCAAACACACTGACGGGGATGCAGCCTTTGCACACTTTTAACACCTTCAGTGGAGGACAAAACAGTACAAATTTACCCCACGGACATTCCACCACTAGAGTATAG

>X_Macaca_mulatta

ATGTCGCGGCCCCAGGGACTGTTATGGCTTCCTCTGTTGTTCACCCCAGTCTGCGTCATGTTAAACTCCAATGTCCTCCTGTGGATAACTGCTCTTGCCATCAAATTCACCCTCATTGACAGCCAAGCACAGTATCCAGTTGTCAACACAAATTATGGCAAAATCCGGGGCCTAAAAACACCGTTACCCAGTGAGATCTTGGGTCCAGTGGAACAGTACTTAGGGGTCCCCTATGCCTCACCCCCCACTGGAGAGAGGCGGTTTCAGCCCCCAGAACCCCCATCCTCCTGGACTGGCATCCGAAATACTACTCAGTTTGCTGCTGTGTGCCCTCAGCACCTGGATGAGAGATCCTTATTGCATGACATGCTGCCCATCTGGTTTACTGCCAATTTGGATACTTTGATGACCTATGTTCAAGATCAAAATGAAGACTGCCTTTACTTAAACATCTACGTGCCCACGGAAGATGGAGCCAACACAAAGAAAAACGCAGATGATATAACCAGTAATGACCGTGGTGAAGATGAAGATATTCATGATCAGAACAGTAAGAAGCCGGTTATGGTCTATATCCATGGGGGATCTTACATGGAGGGCACCGGCAACATGATTGACGGCAGCATTTTGGCCAGCTACGGAAATGTCATCGTGATCACTATTAACTACCGTCTGGGAATACTAGGGTTTTTAAGTACCGGTGACCAGGCAGCAAAAGGCAACTATGGGCTCCTGGATCAGATTCAAGCACTGAGGTGGATTGAGGAGAATGTGGGAGCCTTTGGCGGGGACCCCAAGCGAGTGACCATCTTTGGCTCGGGGGCCGGGGCCTCCTGTGTCAGCCTGTTGACCCTGTCCCACTACTCAGAAGGTCTCTTCCAGAAGGCCATCATTCAGAGCGGCACCGCCCTGTCCAGCTGGGCAGTGAACTACCAGCCGGCCAAGTACACTCGGATATTGGCAGACAAGGTCGGCTGCAACATGCTGGACACCACGGACATGGTAGAATGCCTGCGGAACAAGAACTACAAGGAGCTCATCCAGCAGACCATCACCCCAGCCACCTACCACATAGCCTTCGGGCCGGTGATTGACGGCGACGTCATCCCAGACGACCCTCAGATCCTGATGGAGCAAGGCGAGTTCCTCAACTACGACATCATGCTGGGCGTCAACCAAGGGGAAGGCTTGAAGTTCGTGGACGGCATCGTGGATAACGAGGACGGTGTGACGCCCAATGACTTTGACTTCTCCGTGTCCAACTTCGTGGACAACCTTTACGGCTATCCTGAAGGGAAAGACACTTTGCGCGAGACTATCAAGTTCATGTACACAGACTGGGCCGATAAGGAAAACCCGGAGACACGGCGGAAAACGCTGGTGGCTCTCTTCACTGACCACCAGTGGGTGGCCCCCGCCGTGGCCACAGCCGACCTGCACGCGCAGTACGGCTCCCCCACCTACTTCTATGCCTTCTATCATCACTGCCAAAGCGAAATGAAGCCCAGCTGGGCGGATTCGGCCCATGGCGATGAGGTCCCCTACGTCTTCGGCATCCCCATGGTCGGTCCCACCGAGCTCTTCAGTTGTAATTTCTCCAAGAACGACGTCATGCTCAGCGCCGTGGTCATGACCTACTGGACGAACTTCGCCAAAACTGGTGATCCAAATCAACCAGTTCCTCAGGATACCAAGTTCATTCACACAAAACCCAACCGCTTTGAAGAAGTGGCCTGGTCCAAGTATAATCCCAAAGACCAGCTCTACCTGCATATAGGCTTGAAACCCAGAGTGAGAGATCACTACCGGGCAACGAAAGTGGCTTTCTGGTTGGAACTCGTTCCTCATTTGCACAACTTGAACGAGATATTCCAGTATGTTTCAACAACCACAAAGGTTCCTCCTCCAGACATGACATCATTTCCCTATGGTACCCGCCGATCTCCTGCCAAGATATGGCCAACGACCAAACGCCCAGCAATCACTCCTGCCAACAATCCCAAACACTCTAAGGACCCTCACAAAACGGGGCCCGAGGACACAACTGTCCTCATTGAAACCAAACGGGATTATTCCACTGAATTAAGTGTCACCATTGCCGTCGGGGCATCGCTCCTCTTCCTCAACATTTTAGCCTTTGCGGCTCTGTACTACAAAAAGGACAAGAGGCGCCATGAGACTCACAGGCGCCCGAGTCCCCAGAGAAACACCACAAATGATATCGCTCACATCCAGAACGAAGAGATCATGTCTTTGCAGATGAAGCAGCTGGAGCACGATCACGAGTGTGAGTCGCTGCAGGCACACGACACGCTGAGGCTCACCTGCCCACCAGACTACACCCTCACGCTGCGCCGGTCGCCAGATGATATCCCACTTATGACGCCAAACACCATCACCATGATTCCAAACACACTGACGGGGATGCAGCCTTTGCACACTTTTAACACCTTCAGTGGAGGACAAAACAGTACAAATTTACCCCACGGACATTCCACCACTAGAGTATAG

>Y_Macaca_mulatta

ATGTCGCGGCCCCGGGGACTGCTATGGCTCCCTTTGTTTTTCACCTCCGTCTGTGTCATGTTAAACTCCAATGTCATCTTTTGGATAACTGCTCTTGCCATCAAGTTCACCCTCATTGACAGCCAAGCACAGTATCCAGTTGTCAACACAAATTATGGCAAAATCAGGGGCCTGAAAACACCGTTACCCAGTGAGATCTTGGGTCCAGTGGAGCAGTACTTAGGGGTCCCCTATGCCTCACCCCCCACTGGGGAGAGGCGGTTTCAGCCCCCAGAATCCCCATCCTCCTGGACTGGCATCCGAAATGCTACTCAGTTTGCTGCTGTGTGCCCTCAGCACCTGGATGAGAGATTCTTATTGCATGACATGCTGCCCATCTGGTTTACCCTCAATTTGGATACTTTGATGACCTATGTTCAAGATCAAAATGAAGACTGCCTTTACTTAAACATTTACGTGCCCACGGAAGATGGAACCATCATAAAGAGAAACGATGATGATATAACCAGTAATGACCGTGGTGAAGATAAAGATATTCATGAACAGAATAGTAAGAAGCCAGTTATGGTCTATATCCATGGGGGATCTTACATGGAGGGCACTGGTAACATGATTGATGGCAGCATTTTGGCCAGCTATGGGAACGTCATCGTTATCACCATTAACTACCGTCTGGGAATATTAGGATTTTTAAGTACTGGTGACCAGGCAGCAAAAGGCAACTATGGGCTCCTGGATCAGATTCAAGCACTACGGTGGATTGAGGAGAATGTGGGAGCCTTTGGCGGGGACCCCAAGAGAGTGACCATCTTTGGCTCGGGGGCTGGAGCCTCCTGTGTCAGCCTGTTGACACTGTCCCACTACTCAGAAGGTCTCTTCCAGAAGGCCATCATTCAGAGCGGTACCGCCCTGTCCAGCTGGGCAGTGAACTACCAGCCGGCCAAGTACACTCGGATATTGGCAGACAAGGTCGGCTGCAACATGCTGGACACCACGGACGTGGTAGAATGCCTGCGGAACAAGAACTACAAGGAGCTCATCCAGCAGACCATCACCCCAGCCACCTACCACATAGCCTTCGGGCCCGTGATTGACGGCGACGTCATCCCAGACGACCCTCAGATCCTGATGGAGCAAGGCGAGTTCCTCAACTACGACATCATGCTGGGCGTCAACCAAGGGGAAGGCTTGAAGTTCGTGGACGGCATCGTGGATAACGAGGACGGTGTGACGCCCAATGACTTTGACTTCTCCGTGTCCAACTTCGTGGACAACCTTTACGGCTATCCTGAAGGGAAAGACACTTTGCGCGAGACTATCAAGTTCATGTACACAGACTGGGCCGATAAGGAAAACCCGGAGACGCGGCGGAAAACGCTGGTGGCTCTCTTCACTGACCACCAGTGGGTGGCCCCCGCCGTGGCCACCGCCGACCTGCACGCGCAGTACGGCTCCCCCACCTACTTCTATGCCTTCTATCATCACTGCCAAAGCGAAATGAAGCCCAGCTGGGCGGATTCGGCCCATGGCGATGAGGTCCCCTACGTCTTCGGCATCCCCATGGTCGGTCCCACCGAGCTCTTCAGTTGTAATTTCTCCAAGAACGACGTCATGCTCAGCGCCGTGGTCATGACCTACTGGACGAACTTCGCCAAAACTGGTGATCCAAATCAACCAGTTCCTCAGGATACCAAGTTCATTCACACAAAACCCAACCGCTTTGAAGAAGTGGCCTGGTCCAAGTATAATCCCAAAGACCAGCTCTACCTGCATATAGGCTTGAAACCCAGAGTGAGAGATCACTACCGGGCAACGAAAGTGGCTTTCTGGTTGGAACTCGTTCCTCATTTGCACAACTTGAACGAGATATTCCAGTATGTTTCAACAACCACAAAGGTTCCTCCTCCAGACATGACATCATTTCCCTATGGTACCCGCCGATCTCCTGCCAAGATATGGCCAACGACCAAACGCCCAGCAATCACTCCTGCCAACAATCCCAAACACTCTAAGGACCCTCACAAAACGGGGCCCGAGGACACAACTGTCCTCATTGAAACCAAACGGGATTATTCCACTGAATTAAGTGTCACCATTGCCGTCGGGGCATCGCTCCTCTTCCTCAACATTTTAGCCTTTGCGGCTCTGTACTACAAAAAGGACAAGAGGCGCCATGAGACTCACAGGCGCCCGAGTCCCCAGAGAAACACCACAAATGATATCGCTCACATCCAGAACGAAGAGATCATGTCTTTGCAGATGAAGCAGCTGGAGCACGATCACGAGTGTGAGTCGCTGCAGGCACACGACACGCTGAGGCTCACCTGCCCACCAGACTACACCCTCACGCTGCGCCGGTCGCCGGATGATATCCCACTTATGACGCCAAACACCATCACCATGATTCCAAACACACTGACGGGGATGCAGCCTTTGCACACTTTTAACACCTTCAGTGGAGGACAAAACAGTACAAATTTACCCCACGGACATTCCACCACTAGAGTATAG

>X_Meriones_unguiculatus_(this paper)

ATGACATCGGGCGCCCTGCTGGTGTGCCTAGCGGTGGCCTCCCTGGCCGCATGTGTGTCGTGCTCCTCGCTGCCGCCCGAGGACTCTGGGGAGGGGGCGGGGCCCGTGGTGGTGAGCACGCGATACGGGCGGCTGCGGGGCATGCGCGTGCCCCTGCCGGGCGGCTCTCTGGGCCCCGTGGCACGCTTCCTGGGCGTGCCCTACGCAGCCCCGCCCACCGGCCCTCGCCGCTTCCAGCCGCCTGAGCCTCCCGCCCCTTGGCCCGGCGTGCGAGGGGCCGCGCGCTTCGCCCCGGTGTGCCCCCAGGACGCCGACACACGCCCCGACCCCGCCGCCATGCTGCCCGCCTGGCTCGCCGCTGACCCCGATGCCGTGGCTGCGCACGCGCGCGAGCAAGACGAGGACTGCCTCTACCTCAACCTGTACGTGCCGGCGGGCGTGGGAGGCCATGTGCGGAGCCTCACCGAGGACCTGAGCAACGACGAGCGCGGGGATGACCCAGACACCCGCGACCCAGCCACACGCAAGCCGGTCATGGTGTTCATCCACGGCGACTCATACATGGCAGGCACGGGGAACATGATGGACGGCAGTGTGCTGGCGAGCTATGGCGATGTCATTGTGGTCACGCTCAACTACCGGCTGGGCGCGCTCGGCTTCCTGAGTACGGGCGACCCTGCAGCGCGTGGGAACTACGGGCTGCTGGACCAGATGCAGGCGCTTCGCTGGCTGCGTGAGAACGCGGTCGCCTTCGGCGGGGATCCCGCCCGTGTCACGGTCTTCGGCTCAGGCGCCGGAGCCTCGTGCGTGAGCCTGCTCACGCTGTCGCACTACTCAGAAGGCCTGTTCCAGAAGGCCATCATCCAGAGCGGCACCGCACTGTCCAGCTGGGCTGTCAACTACCAGCCGGCGGCGTATGCGCGCATGCTCGGAGCCCGTGTAGGCTGCGGAGGAGACATGACGTCGGCGACCTCGCCCCCGGACACCATGGCGACGCCTCCCTTGACGTCCTCGGTGCACGACCCGCCCTCCCCGTCAGCTGCGCTAGTGGCCTGCCTCCGCCGCCGAGGCGCCCGCGAGCTGACCCGGGCTGCGGGTTCGGTACCCGCGTCCGCGCCCTTCCACGTGGCCTTCGGGCCAGTGATCGATGGTGATGTGGTGCCTGACGACCCGCAGATCCTCATGGAGCAGGGTGAGTTCCTCAACTACGACATCCTTCTGGGCGTCAACCAGGCGGAGGGCGTGGCCCTGGCCGACCCCGCCCACCCGGACGGCGGCGGCGACGTCACAGCGGATGGCGAAGAGGAGGAGGAGGTGTCGGCTGCAGGCTTCGAACTCGCTGTTGCCGCCTTCGTGGATGCGCTGTACGGCTACCCGGGAGGGGATGTGGGCGTGGCCGGACTGGGCGGGGGCGTGGCCGGCTGGGGCAGTGGAGCTGGCGGGGACTCGGCCCTTCGCGAGACGGCGCGCTTCATGTACACGGACTGGGCGGAGCGCGAGGGCGGGGCGGGGTCACGGCGCCGCGCCCTGGCGGCCATGATGACGGACCACCAGTGGGCGGCGCCCGCCGTGGCCACCGCGGACTTGCACGCCCGGTACGGCTCGGCCACCTACTTCTACGCCTTTGCACACCCGTGTCGGGGGGACGCGCACCCCGCCTGGGCGGCCGAAGCGGGCGCGGCTCATGGTGACGAGCTGCCCTTCGTGTTCGGGGTCCCGATGCTCGTGCTGGCGGCGGCCGGGGGTGGGGTTGGAGGAGTCGGAAGCGAGGGCGCGGCCGGAAGTGACGTCGCCGTGGCCACCGCCGCCAATGCTGCCGCCCTCTTCCCGTGCAACTTCACGCGTAATGACGTAATGCTCAGCGCCGTCGTCATGACGTACTGGACCAACTTCGCCAAGACGGGGGACCCCAACCGTCCGGTGCCACAGGACACCAAGTTTGCGCACACACGTCCCAACCGCTTCGAGGCAGTGGCCTGGCCAAAGTACACACCACGGGAGCGCCTCTACCTGCACGTCGGGCTGCGGCCGCGTGTGCGTGACCACTACAGAGCCACCAAGGTGGCCTTCTGGCTGGAGCTGGTGCCGCATCTACACGGGCTGCGCGAGGCATTTCCCTACCTGACCACACCTACCGCCGCCCCACGTGCCCAGCCTGGCCCCCGCAGGGCGTGGCCGCCCACCCGCCGGCCTGCCCCACCCTCCTCAGGGAGGCCCGCCTCTTCCTCCTCCTCCTCTGCGTCAGCTTCATCCTCGCGGGACTCAAACCCAGGGCCAGGCGAGGCCTCGGTGCTCATTGAGACGCGCCGTGACTACTCCACGGAGCTCAGCGTCACCATTGCCGTGGGCGCCTCCCTTCTCTTCCTCAACATCCTGGCCTTCGCTGCCCTCTACTACAAGAAGGACAAGCGGCGCCATGAGACCCACCACCGAAGGATGGCTGCTTCCGGTGGCACTTCAGGGTTCGCGTCCGGGCTCACTTCCGCTTCTGGTGCAACTTCCGGTTCGACTTCCGGGTTTGCCTCTGGTCCCACTTCTGGTTTTGCTTCTACTTCTGGTCCCACTTCCGGATATACTGCTGCCCACCGCCCTGGAAACGATGCCCTGAAACGTGGGCGGGAGGAGGACCCCGGGGCAGCAGTGACATCGCCCTCATCCCTCGATGCCCTGCGGCTGCCCACTGGCCCGCCAGACTACGCACTTACGCTGCGTCGTGCCCCCGACGACGCTGCACCCCTTGCTGTGCCCAGTGCCATCACCATGGTGCCCAATGCGCTGGCCGGGCTCCCACCACTGCACGCCTTTGGCCACTCCACCACTCGGGTATAG

>Y_Meriones_unguiculatus_(this paper)

ATGACATTGGGTGCGCTGCTGGTGTGCCTAGCTGTGGCCTCCCTGGCCGCATGTGTGTCCTGCTCCTCGCTGCCGCCCGAGGACTCTGGGGAGGGGGCAGGGCCCGTGGTGGTGAGCACGCGATACGGGCTGCTGCGGGGCATGCGCGTGCCCCTGCCGGGCGGCTCTCTGGGCCCCGTAGCACGCTTCCTGGGCGTGCCCTACGCCGCCCCGCCCACCGGCCCTCGCCGCTTCCAGCCGCCTGAGCCTCCGGCCCCGTGGCCCGGCGTGCGAGGGGCCGCGCGCTTCGCCCCGGTGTGCCCCCAGGACGCCGACACGCGCCCCGACCCCGCCGCCATGCTGCCCGCCTGGCTCGCCGCCGACCCCGATGCCGTGGCTGCGCACGCGCGCGAGCAAGACGAGGACTGCCTCTACCTCAACCTGTACGTGCCGGCGGGCGTGGGAGGGCACCTGCGGAACCTCGCGGATGACCCAAGCAGCGACGAGCGTGGGGACGACCCAGACACCCGCGACCCAGCCACACGCAAGCCAGTCATGGTGTTCATCCACGGCGACTCATACATGGCAGGCACGGGGAACATGATGGACGGCAGTGTGCTGGCGAGCTATGGCGATGTCATTGTGGTCACGCTCAACTACCGGCTGGGCGCGCTCGGCTTCCTGAGCACGGGCGACCCTGCAGCGCGTGGGAACTACGGGCTGCTGGACCAGATGCAGGCGCTTCGCTGGCTGCGTGAGAACGCGGTCGCCTTCGGCGGGGATCCCGCCCGTGTCACGGTCTTCGGCTCGGGCGCCGGAGCCTCGTGCGTGAGCCTGCTCACGCTGTCGCACTACTCGGAAGGCCTGTTCCAGAAGGCCATCATCCAGAGCGGCACCGCACTGTCCAGCTGGGCTGTCAACTACCAGCCGGCGGTGTATGCACGCATGCTCGGGGCCCGTGTGGGCTGCGGGGGAGACGTGATGTCGGCGACCCCGCTCCCGGACGCTGCGGCGACGCCACCCCGGATGTCCTCGTCTCACGATCTGCCTTCCGCATCAGCTGCGCTAGTGGCCTGCCTCCGTCGCCGTGGGGCCCGCGAGCTGACCCGGGCCGCGGGTTCGGTGCCCGCGTCCTCACCATTCCATGTGGCCTTCGGGCCAGTGATCGATGGAGATGTGGTGCCGGACGACCCGCAGATCCTCATGGAGCAGGGTGAGTTCCTCAACTACGACATCCTTCTGGGCGTCAACCAGGCGGAGGGCGTGGCCCTGGCAGACCCCGCCCACCCCGACAGCCTCGGGGATATAATGGCTGACGGTGAGGAGGAGGTGTCGGCTGCCGGCTTCGAACTCGCTGTTGCTGCCTTCGTGGATGCGCTGTACGGCTACCCAGGAGGGGATGTGGGCGTGGCCGGCTGGAGCGGTGGAGCCGGCGGGGACTCCGCCCTTCGCGAGACGGCGCGCTTCATGTACACGGACTGGGCGGAGCGCGAGGGCGGGGCGGGGTCACGGCGCCGCGCCTTGGCGGCCATGATGACGGACCACCAGTGGGCGGCGCCCGCCGTGGCCACGGCCGACTTGCACGCCCGGTACGGCTCGGCCACCTACTTCTACGCCTTTGCACACCCGTGTCGGGGGGACGCGCACCCCGCCTGGGCGGCCGAAGCGGGCGCTGCCCATGGCGACGAGCTGCCCTTCGTATTCGGGGTCCCGATGCTCGTGCTGGCGGCGGCCGGTGATGGCAGTGTCGGAGGAGTCGGAGGCGAAGGCGCAACGGGAACTGATGTCGCTGCGGCCACCGCCGCTGCCCTCTTTCCATGCAACTTCACGCGCAATGACGTGATGCTCAGCGCCGTCGTCATGACGTACTGGACCAACTTCGCCAAGACCGGGGACCCCAACCGTCCGGTACCCCAGGACACCAAGTTTGCGCACACACGCCCCAACCGCTTCGAGGCAGTAGCCTGGCCCAAGTACACTCCCCGAGAGCGCCTCTACTTGCACGTGGGACTACGGCCACGCGTGCGTGACCACTACCGTGCTACCAAGGTGGCTTTCTGGCTAGAGTTGGTGCCACATCTTCATGGGCTTCGTGAGGCCTTCCCCTACCTGACCACGCCGACAGCTGCTCCACACGTCCCGACTGGACCGCGCAGGGTGTGGCCGCCCACGCGCCGCCCAGCTCTGCCCTCCTTGGGGAGGCCAGCCTCCTCCTCCTCCGCATCATCCTCGTCCTCACAGGACTCGAAAGTGGGTCCAGGCGAGGCTGTGCTCATCGAAACTCGCCGGGACTACTCTACGGAGCTCAGCGTCACCATCGCTGTAGGTGCTTCTCTCCTCTTCCTCAACATCCTGGCCTTCGCTGCCCTCTACTACAAGAAGGACAGGCGGCGCCACGAGACCCACCACCGAAGGATGGCTGCTTCTGGTGCAACTTCAGGGTTCGCTTCGGCTTCCGGGACAGCTTCTGGCCCAACTTCCGATTTTGTGTCAGCTTCTGGGCACGCTTCTGGTCCCGCTTCGGGTTTTCCTTCTACTTCTGGTCCCACTTCCGGATTTGCTTCCACCCACTGTTCCGGAAATGACACCGGTAAGCGAGGCCGAGAGGAGAACCCCGGGGCGATGGTGACATCATCGGTGACATCGCCCTCCTCCCTTGATGCCGCCCTTCGGCTGCCCACCGGCCCACCTGACTACACGCTCACGCTGCGCCGTGCCCCAGATGATGCCCCACCCCTCACTGCGCCTAGTGCTATCACCATGATGCCCAACGCACTGGCTGGGCTCCCACAGCTTCACACCTTTGGTCACTCCACCACACGGGTATAG
