# Supplementary material for: Of Humans and Gerbils— Independent Diversification of Neuroligin-4 Into X- and Y-Specific Genes in Primates and Rodents
Source: Front Mol Neurosci. 2022 Mar 30;15:838262. doi: 10.3389/fnmol.2022.838262 (PMC9005811; doi:10.3389/fnmol.2022.838262)
Supplement: Supplementary file 7 [file Image_1.pdf]

```
NLGN4X_Mun     MTSGALLVCLAVASLAACVSCSSLPPEDSGEGAGPVVVSTRYGRLRGMRVPLPGGSLGPVARFLGVPYAAPPTGPRRFQP
NLGN4Y_Mun     MTLGALLVCLAVASLAACVSCSSLPPEDSGEGAGPVVVSTRYGLLRGMRVPLPGGSLGPVARFLGVPYAAPPTGPRRFQP
                  signal peptide                      exon 1
               81                                                                           160
NLGN4X_Mun     PEPPAPWPGVRGAARFAPVCPQDADTRPDPAAMLPAWLAADPDAVAAHAREQDEDCLYLNLYVPAGVGGHVRSLTEDLSN
NLGN4Y_Mun     PEPPAPWPGVRGAARFAPVCPQDADTRPDPAAMLPAWLAADPDAVAAHAREQDEDCLYLNLYVPAGVGGHLRNLADDPSS
                                                                                       exon 3
               161                                                                          240
NLGN4X_Mun     DERGDDPDTRDPATRKPVMVFIHGDSYMAGTGNMMDGSVLASYGDVIVVTLNYRLGALGFLSTGDPAARGNYGLLDQMQA
NLGN4Y_Mun     DERGDDPDTRDPATRKPVMVFIHGDSYMAGTGNMMDGSVLASYGDVIVVTLNYRLGALGFLSTGDPAARGNYGLLDQMQA
                                          exon 4                                exon 5
               241                                                                          320
NLGN4X_Mun     LRWLRENAVAFGGDPARVTVFGSGAGASCVSLLTLSHYSEGLFQKAIIQSGTALSSWAVNYQPAAYARMLGARVGCGGDM
NLGN4Y_Mun     LRWLRENAVAFGGDPARVTVFGSGAGASCVSLLTLSHYSEGLFQKAIIQSGTALSSWAVNYQPAVYARMLGARVGCGGDV
                                                                             exon 6
               321                                                                          400
NLGN4X_Mun     TSATSPPDTMATPPLTSSVHDPPSPSAALVACLRRRGARELTRAAGSVPASAPFHVAFGPVIDGDVVPDDPQILMEQGEF
NLGN4Y_Mun     MSATPLPDAAATPPRMSSSHDLPSASAALVACLRRRGARELTRAAGSVPASSPFHVAFGPVIDGDVVPDDPQILMEQGEF
                                                                                           Nrxn-
               401                                                                          480
NLGN4X_Mun     LNYDILLGVNQAEGVALADPAHPDGGGDVTADGEEEEEVSAAGFELAVAAFVDALYGYPGGDVGVAGLGGGVAGWGSGAG
NLGN4Y_Mun     LNYDILLGVNQAEGVALADPAHPDSLGDIMADGEEE--VSAAGFELAVAAFVDALYGYPGGDVGVAG-------WSGGAG
               binding site
               481                                                                          560
NLGN4X_Mun     GDSALRETARFMYTDWAEREGGAGSRRRALAAMMTDHQWAAPAVATADLHARYGSATYFYAFAHPCRGDAHPAWAAEAGA
NLGN4Y_Mun     GDSALRETARFMYTDWAEREGGAGSRRRALAAMMTDHQWAAPAVATADLHARYGSATYFYAFAHPCRGDAHPAWAAEAGA

               561                                                                          640
NLGN4X_Mun     AHGDELPFVFGVPMLVLAAAG-GGVGGVGSEGAAGSDVAVATAANAAALFPCNFTRNDVMLSAVVMTYWTNFAKTGDPNR
NLGN4Y_Mun     AHGDELPFVFGVPMLVLAAAGDGSVGGVGGEGATGTDVAAATAA---ALFPCNFTRNDVMLSAVVMTYWTNFAKTGDPNR

               641                                                                          720
NLGN4X_Mun     PVPQDTKFAHTRPNRFEAVAWPKYTPRERLYLHVGLRPRVRDHYRATKVAFWLELVPHLHGLREAFPYLTTPTAAPRAQP
NLGN4Y_Mun     PVPQDTKFAHTRPNRFEAVAWPKYTPRERLYLHVGLRPRVRDHYRATKVAFWLELVPHLHGLREAFPYLTTPTAAPHVPT
                                           exon 7
               721                                                                          800
NLGN4X_Mun     GPRRAWPPTRRPAPPSSGRPASSSSSSASASSSRDSNPGPGEASVLIETRRDYSTELSVTIAVGASLLFLNILAFAALYY
NLGN4Y_Mun     GPRRVWPPTRRPALPSLGRPASSSS--ASSSSSQDSKVGPGEA-VLIETRRDYSTELSVTIAVGASLLFLNILAFAALYY
                                                                         transmembrane region
               801                                                                          880
NLGN4X_Mun     KKDKRRHETHHRRMAASGGTSGFASGLTSASGATSG--STSGFASGPTSGFASTSGPTSGYTAAHRPGNDALKRGREEDP
NLGN4Y_Mun     KKDRRRHETHHRRMAASGATSGFASASGTASGPTSDFVSASGHASGPASGFPSTSGPTSGFASTHCSGNDTGKRGREENP

               881                                                              950
NLGN4X_Mun     GA----AVTSPSSLDA-LRLPTGPPDYALTLRRAPDDAAPLAVPSAITMVPNALAGLPPLHAFGHSTTRV
NLGN4Y_Mun     GAMVTSSVTSPSSLDAALRLPTGPPDYTLTLRRAPDDAPPLTAPSAITMMPNALAGLPQLHTFGHSTTRV
                                                                                PDZ BM
```

Suppl. Figure 1. Alignment of gerbil NLGN4X and NLGN4Y protein sequences.
Several key features of the Mongolian gerbil, Meriones unguiculatus (Mun), neuroligin-4 protein sequences are depicted: exon/exon junctions, indigo; signal peptide, yellow; critical neurexin (Nrxn) binding site (Araç et al., 2007), green; PDZ binding motif (PDZ BM), purple; transmembrane region, grey. The respective name and number of each encoding exon is displayed below the protein sequence.
