# Supplementary material for: Of Humans and Gerbils— Independent Diversification of Neuroligin-4 Into X- and Y-Specific Genes in Primates and Rodents
Source: Front Mol Neurosci. 2022 Mar 30;15:838262. doi: 10.3389/fnmol.2022.838262 (PMC9005811; doi:10.3389/fnmol.2022.838262)
Supplement: Supplementary file 8 [file Image_2.pdf]

```
             1                                                                              80
NLGN4a_Dsp   MEGRVAWWTWTLCLVVTPSTLGGQGSEDDPVVRTQYGHLRGLRASLPSELLGPVQQFLGIPYAAPPVGPRRFLPPEPPTA
NLGN4b_Dsp   MEGRVAWWTWTLCLVVTPSTLGGQGSEDDPVVRTQYGHLRGLRASLPSELLGPVQQFLGIPYAAPPVGPRRFLPPEPPTA
                 signal peptide                          exon 1

             81                                                                            160
NLGN4a_Dsp   WPGIRNATHFAPVCPQRLDERTLPRDMLPSWLSANLETVAGLLREQSEDCLFLNVYVPTEDDIHEPGARRPVMVYIHGGS
NLGN4b_Dsp   WPGIRNATHFAPVCPQRLDERTLPRDMLPSWLSANLETVAGLLREQSEDCLFLNVYVPTEDDIHEPGARRPVMVYIHGGS
                                                                                     exon 4

             161                                                                           240
NLGN4a_Dsp   YMEGTGNMIDGSVLASYGNVIVITLNYRLGILGFLSTGDQAAKGNYGLLDQIQALRWVEENVGAFGGDPKRVTIFGSGAG
NLGN4b_Dsp   YMEGTGNMIDGSVLASYGNVIVITLNYRLGILGFLSTGDQAAKGNYGLLDQIQALRWVEENVGAFGGDPKRVTIFGSGAG
                                                                       exon 5

             241                                                                           320
NLGN4a_Dsp   ASCVSLLTLSHYSEGLFQKAIIQSGTALSSWAVNYQPARYARALAAQLGCPSPADTSALVSCLRLKSPQELTRPVVTPAT
NLGN4b_Dsp   ASCVSLLTLSHYSEGLFQKAIIQSGTALSSWAVNYQPARYARALAAQLGCPSPADTSALVSCLRLKSPQELTRPVVTPAT
                                         exon 6

             321                                                                           400
NLGN4a_Dsp   YHVAFGPVIDGDVIPDDPQILMEQGEFLNYDILLGVNQGEGLGFVDGLVDALDDGVSAAAFEASIAAFVDHLYGYPEGKQ
NLGN4b_Dsp   YHVAFGPVIDGDVIPDDPQILMEQGEFLNYDILLGVNQGEGLGFVDGLVDALDDGVSAAAFEASIAAFVDHLYGYPEGKQ
                                    Nrxn-binding site

             401                                                                           480
NLGN4a_Dsp   ALRETIKFMYTDWADRDNPETRRKTLVALFTDHQWVAPAVATADLHAQYGSPTYFYAFYHRCQSELKPAWADAAHGDEVP
NLGN4b_Dsp   ALRETIKFMYTDWADRDNPETRRKTLVALFTDHQWVAPAVATADLHAQYGSPTYFYAFYHRCQSELKPAWADAAHGDEVP

             481                                                                           560
NLGN4a_Dsp   YVFGVPMVGPTELFSCNFSKNDVMLSAVVMTYWTNFAKTGDPNQPVPQDTKFIHTKPNRFEEVAWSKYNPRDQLYLHIGL
NLGN4b_Dsp   YVFGVPMVGPTELFSCNFSKNDVMLSAVVMTYWTNFAKTGDPNQPVPQDTKFIHTKPNRFEEVAWSKYNPRDQLYLHIGL
                                                                       exon 7

             561                                                                           640
NLGN4a_Dsp   KPRVRDHYRATKVAFWLELVPHLHNLNDILQYVSTTTTRAPDVTSSSSSSHPRRATKRPTSSSSSILGPKALRPEGKLRQ
NLGN4b_Dsp   KPRVRDHYRATKVAFWLELVPHLHNLNDILQYVSTTTTRAPDVTSSSSSSHPRRATKRPTSSSSSILGPKALRPEGKLRQ

             641                                                                           720
NLGN4a_Dsp   GGTEHSTTTVLIETKRDYSTELSVTIAVGASLLFLNILAFAALYYKKDKRRHQTHRRPSSPPSTSTRPPAPQDAAQRHLL
NLGN4b_Dsp   GGTEHSTTTVLIETKRDYSTELSVTIAVGASLLFLNILAFAALYYKKDKRRHQTHRRPSSPPSTSTRTPAPQDAAQRHLL
                                     transmembrane region

             721                                                                           800
NLGN4a_Dsp   RASAPTELLSVQLNPNVGAHEARDLQDALHLTCPPDYALTLRRSPDDIPLMTPSTITTPGPTLHTFNTFGGAGGGGVGGP
NLGN4b_Dsp   RASAPTELLSVQLNPNVGAHEARDLQDALHLTCPPDYALTLRRSPDDIPLMTPSTITTPGPTLHTFNTFGGAGGGGVGGP

             801                 820
NLGN4a_Dsp   GGGSGGQNNPLPHAHSTTRV
NLGN4b_Dsp   GGGSGGQNNPLPHAHSTTRV
                            PDZ BM
```

Suppl. Figure 2. Alignment of kangaroo rat NLGN4X and NLGN4Y protein sequences. Key features of the Banner-tailed kangaroo rat, Dipodomys spectabilis (Dsp), neuroligin-4 protein sequences are depicted: exon/exon junctions, indigo; signal peptide, yellow; critical neurexin (Nrxn) binding site (Araç et al., 2007), green; PDZ binding motif (PDZ BM), purple; transmembrane region, grey. The respective name and number of each encoding exon is displayed below the protein sequence. Both NLGN4 proteins are depicted as variants “a” and “b”. A lack of genomic sequence information does not allow any further distinction.
