# Supplementary material for: Of Humans and Gerbils— Independent Diversification of Neuroligin-4 Into X- and Y-Specific Genes in Primates and Rodents
Source: Front Mol Neurosci. 2022 Mar 30;15:838262. doi: 10.3389/fnmol.2022.838262 (PMC9005811; doi:10.3389/fnmol.2022.838262)
Supplement: Supplementary file 10 [file Image_4.pdf]

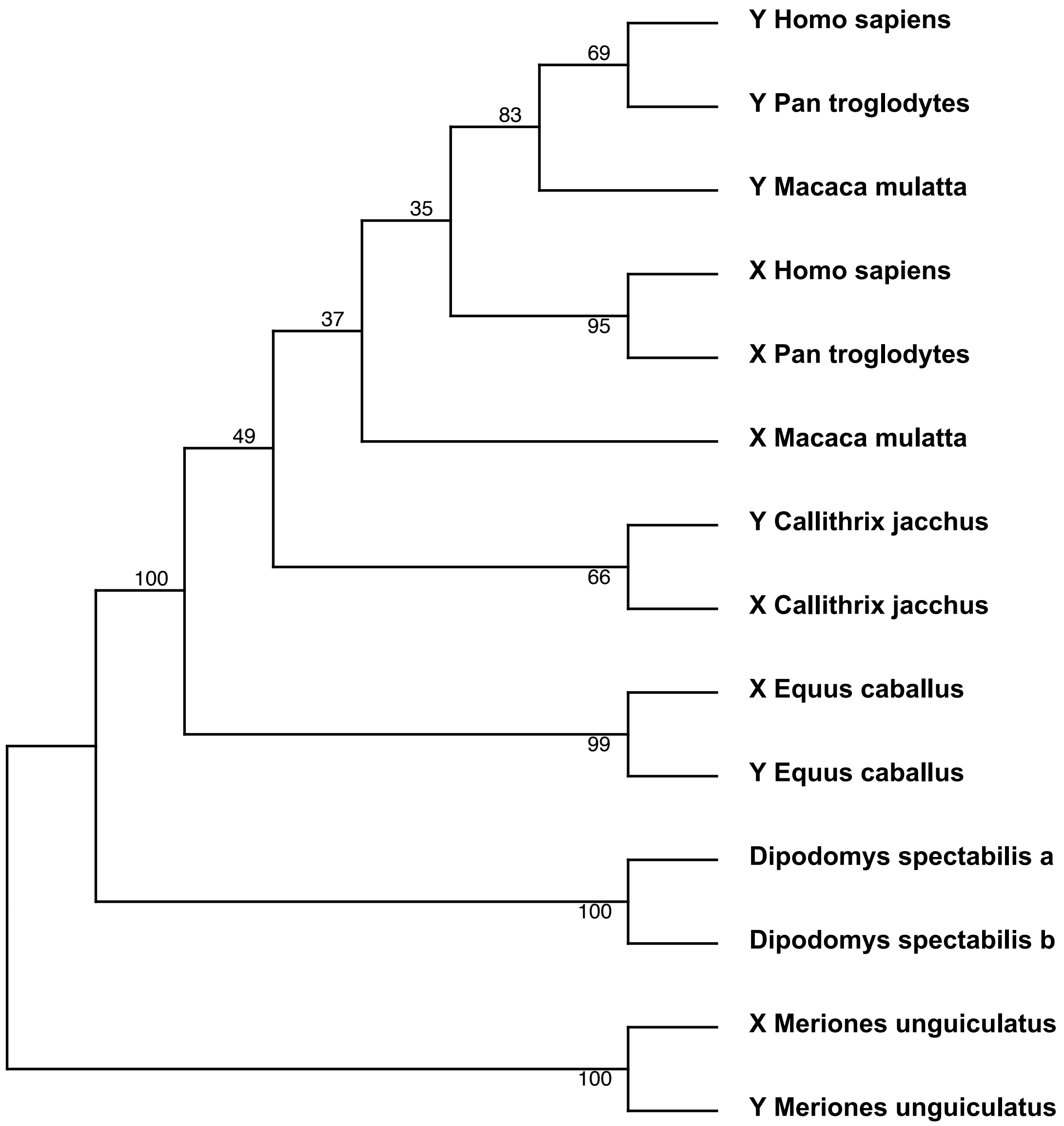

Suppl. Figure 4. Node statistics accompanying Figure 5
Displayed are the branches of the evolutionary tree depicted in Figure 5. the branches are not drawn to scale allowing the documentation of the node statistics for each branching point.
